# Iron status and sarcopenia-related traits: a bi-directional Mendelian randomization study

**DOI:** 10.1038/s41598-024-60059-w

**Published:** 2024-04-22

**Authors:** Honggu Chen, Ziyi Zhang, Yizhe Wang, Anpei Ma, Lingbo Li, Guoyang Zhao

**Affiliations:** 1 Department of Orthopedics, the Affiliated Hospital of Jiangsu University, Zhenjiang, 212000 Jiangsu Province People’s Republic of China; 2 School of Medicine of Jiangsu University, Zhenjiang, 212000 Jiangsu China; 3 Department of Orthopedics, Yancheng First People’s Hospital, Yancheng, 224000 Jiangsu Province People’s Republic of China; 4 Department of Internal Medicine, Peking Union Medical College Hospital, Beijing, 100730 Beijing, People’s Republic of China

**Keywords:** Iron status, Ferritin, Sarcopenia, Multivariable mendelian randomization, Causality, Clinical genetics, Genomics, Nutrition, Predictive markers

## Abstract

Although serum iron status and sarcopenia are closely linked, the presence of comprehensive evidence to establish a causal relationship between them remains insufficient. The objective of this study is to employ Mendelian randomization techniques to clarify the association between serum iron status and sarcopenia. We conducted a bi-directional Mendelian randomization (MR) analysis to investigate the potential causal relationship between iron status and sarcopenia. MR analyses were performed using inverse variance weighted (IVW), MR-Egger, and weighted median methods. Additionally, sensitivity analyses were conducted to verify the reliability of the causal association results. Then, we harvested a combination of SNPs as an integrated proxy for iron status to perform a MVMR analysis based on IVW MVMR model. UVMR analyses based on IVW method identified causal effect of ferritin on appendicular lean mass (ALM, β = − 0.051, 95% CI − 0.072, − 0.031, p = 7.325 × 10^–07^). Sensitivity analyses did not detect pleiotropic effects or result fluctuation by outlying SNPs in the effect estimates of four iron status on sarcopenia-related traits. After adjusting for PA, the analysis still revealed that each standard deviation higher genetically predicted ferritin was associated with lower ALM (β = − 0.054, 95% CI − 0.092, − 0.015, *p* = 0.006). Further, MVMR analyses determined a predominant role of ferritin (β = − 0.068, 95% CI − 0.12, − 0.017, *p* = 9.658 × 10^–03^) in the associations of iron status with ALM. Our study revealed a causal association between serum iron status and sarcopenia, with ferritin playing a key role in this relationship. These findings contribute to our understanding of the complex interplay between iron metabolism and muscle health.

## Introduction

Sarcopenia is a progressive syndrome which is characterized by a decline in muscle mass and strength function of the whole body, accompanied by a decline in quality of life and an increase in mortality^1^. It occurs commonly as an age-related process in older people. Sarcopenia significantly impacts daily activities, functional status, contributes to increased disability, and affects quality of life in older populations^2^. It also elevates healthcare costs^3^, and imposes a substantial burden on both individual health and the social economy^4^. It is estimated that there are currently about 50 million people with sarcopenia in the world, and this number is expected to reach 500 million by 2050 as the world population ages rapidly^5^. With growing life expectancy, the prevalence of sarcopenia will continuously increase during the next decades^6^. The incidence of sarcopenia is predicted to increase to > 200 million affected older adults worldwide over the next 40 years^3^, highlighting the urgency for understanding risk factor.

Iron as an essential trace element has very important biological functions in the body^7^, is an essential micronutrient for many biochemical processes such as oxidative energy metabolism, electron transfer reactions, gene regulation, binding and transport of oxygen^8^, it plays a pivotal role in cell survival and proliferation^9^. Iron in the body is in a dynamic balance of constant absorption, utilization, storage, recycling, namely iron homeostasis^10^. Iron dyshomeostasis can lead to diseases related to iron metabolism, resulting in damage of organism, including iron deficiency and iron overload^11^. Several studies reported that iron deficient caused anemia, neurocognitive dysfunction, and impaired functional capacity energy metabolism abnormality^12–14^, whereas iron overload resulted in osteoporosis, neurodegeneration, cardiovascular diseases and hepatic disease^15–18^. Iron overload is prone to occur with age^19^. Ferritin, the primary protein responsible for iron storage^20^, often exhibits increased levels as one ages^21^. While the majority of iron is stored in the liver and spleen^22^, skeletal muscle also contains iron, albeit in smaller amounts. Excessive iron in the body, or iron overload, could potentially have adverse effects on skeletal muscle health^23^.

Iron status and its relationship with sarcopenia have been investigated in previous studies, yielding inconsistent findings. For instance, an observational study reported lower serum iron levels in individuals with sarcopenia compared to those without sarcopenia^24^. Conversely, other studies have demonstrated a significant association between serum ferritin and transferrin saturation with reduced grip strength, while serum iron did not show such an association^25,26^. These discrepancies in previous findings may be attributed to limitations inherent in observational studies^27^, including susceptibility to potential confounding factors and the challenge of establishing causal relationships between iron status and sarcopenia.

Mendelian randomization (MR) is a method that can overcome problems of unmeasured confounding and reverse causation typical of conventional observational epidemiology^28^, it assess causal inference of an exposure on an outcome by using genetic variants as instrumental variables for the exposure^29^. genetic variants are randomly allocated at conception, so they can be exploited to simulate randomization^30^.

The aim of this study is to employ a bi-directional two sample Mendelian randomization (MR) analysis, utilizing four iron-related biomarkers as clinical indicators of iron status, to comprehensively assess the causal association between iron status and sarcopenia. Additionally, a multivariable Mendelian randomization (MVMR) analysis will be conducted to determine the potential dominant role of any specific trait in the causation process. To our current knowledge, a paucity of Mendelian randomization (MR) investigations exists to scrutinize the causal effects pertaining to iron status and sarcopenia.

## Materials and methods

### Overall study design

**Figure 1:**
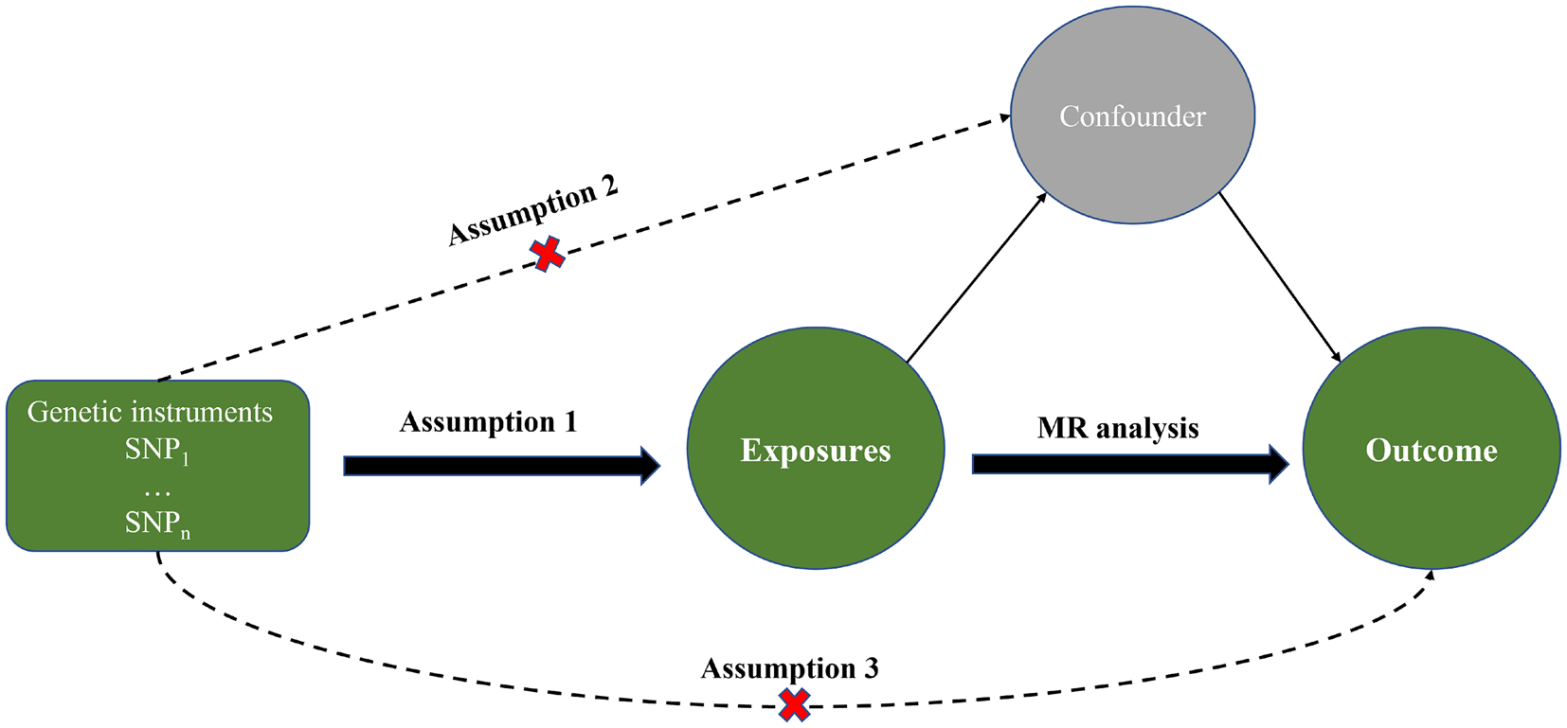
An overview of the study design includes three key assumptions.MR satisfies the following three conditional assumptions^32^: (1) there is a strong association between instrumental variables and exposure factors; (2) no confounding factors exist in the association between exposure and outcome, in other words, there is no genetic pleiotropy; and (3) the instrumental variables do not have a direct effect on outcome and can only influence outcome through exposure factors.

In this MR study, we utilized a series of analyses approach to investigate the association between iron status and sarcopenia. Univariable Mendelian randomization (UVMR) analysis and Multivariable Mendelian randomization (MVMR) analysis were employed. The analysis was conducted using summary-level data from published genome-wide association studies (GWASs), and all studies included in cited GWASs had been approved by relevant review boards and obtained informed consent from participants. The present MR analyses were conducted in accordance with the STROBE-MR guidelines^31^. The study design is illustrated in Fig. 1.

### Data sources

The source of exposure data for our study on iron status-related indicators was obtained from a meta-analysis of three genome-wide association studies from Iceland, the UK, and Denmark^33^. The GWAS summary data included blood levels of ferritin (N = 246,139), total iron binding capacity (N = 135,430), iron (N = 163,511), and transferrin saturation (N = 131,471). These four iron-related biomarkers were each rank-based inverse normal transformed to a standard normal distribution, separately for each sex, and adjusted for age using a generalized additive model. Furthermore, the UK cohort was adjusted for menopausal status, ABO blood group, BMI, smoking levels, alcohol levels, and iron supplementation status.

Summary genetic association estimates for sarcopenia were obtained from MRCIEU GWAS database. We used appendicular lean mass (N = 450,243), right hand grip strength (N = 461,089), left hand grip strength (N = 461,026), low hand grip strength (60 years and older) (N = 256,523) and walking pace (N = 459,915) as genetically predicted sarcopenia-related traits. In particular, low hand grip strength, which is defined by the European Working Group on Sarcopenia in Older People (EWGSOP) as grip strength < 30 kg in men and < 20 kg in women, was used as a measure of sarcopenia in a meta-analysis comprising 256,523 individuals aged 60 years or older from 22 independent cohorts of European descent, including the UK Biobank, the US Health and Retirement Study, and the Framingham Heart Study, among others, with 18.9% of participants (46,596 individuals) exhibiting muscle weakness. Details of the included traits are displayed in Table 1.

**Table 1:** Details of studies and datasets used in the study.

Trait	PMID	Sample size	Unit	Datatype	Ancestry	Consortium/ study	Year of publication
Ferritin	33,536,631	246,139	SD (1.08 µg/L)	Continuous	European	GIS	2021
serum iron	33,536,631	163,511	SD (7.76 μmol/L)	Continuous	European	GIS	2021
TIBC	33,536,631	135,430	SD (14.14 μmol/L)	Continuous	European	GIS	2021
TSAT	33,536,631	131,471	SD (13.25%)	Continuous	European	GIS	2021
Low hand grip strength	33,510,174	256,523 (48,596 cases and 207,927 controls)	/	Binary	European	UKB	2021
Walking pace	25,826,379	459,915	SD	Continuous	European	UKB	2018
Appendicular lean mass	33,097,823	450,243	kg	Continuous	European	UKB	2020
Hand grip strength (right)	25,826,379	461,089	SD	Continuous	European	UKB	2018
Hand grip strength (left)	25,826,379	461,026	SD	Continuous	European	UKB	2018
Physical activity	35,534,559	78,007	risk difference	Continuous	European	Within family GWAS consortium	2022

*TIBC* total iron binding capacity, *TSAT* transferrin saturation, *GIS* genetics of iron status, *UKB* the UK Biobank, *SD* standard deviation.

As both exposure and outcome data were partially obtained from UKBs, we assessed sample overlap rate and type 1 error rate using a web-based application (https://sb452.shinyapps.io/overlap/^34^ to ensure the validity of our results. The results showed that the overlap rate in this study ranged from 8.7 to 15.6%, and all the type 1 error rates were less than 0.05, which suggested that our subsequent analyses were reliable and robust.

### Instrumental variable selection

To meet the relevance assumption, the first of the three key assumptions, instrumental variants should be associated with the exposure factors^28^. The single nucleotide polymorphisms (SNPs) associated with exposures were extracted at a genome-wide significance level (*p* < 5 × 10^–8^) from the GWAS datasets^35^. Afterwards, independent SNPs for exposures were obtained by linkage disequilibrium (LD) clumping with a threshold r^2^ < 0.001 and an allele distance > 10,000 kb^36^. We then extracted the SNPs and corresponding statistics from the GWAS dataset of outcomes, removing the SNPs with a minor allele frequency (MAF) < 0.01^37^. We employed proxy SNPs with a high correlation coefficient (R^2^ > 0.8) as a substitute for the missing SNPs. Further, we harmonized the data by removing all palindromic SNPs^38^. To fulfill the second MR assumption, we inquired for each IV and its proxy traits referring to PhenoScannerV2 database (http://www.phenoscanner.medschl.cam.ac.uk/) and discarded the SNPs surrogating for these confounding traits at a threshold of r^2^ > 0.80^39,40^. Accordingly, these rigorously selected SNPs were used as IVs for the following analyses.

### Instrumental strength

We computed the proportion of phenotypic variation that is explained by all SNPs (*i.e.*, R^2^-values) in our MR analysis using the formula R^2^ = 2 × β^2^ × EAF × (1 − EAF)/[2 × β^2^ × EAF × (1 − EAF) + SE^2^ × 2 × N × EAF × (1-EAF)] where β represents the effect estimate of the genetic variant in the exposure GWAS and EAF is the Allele 1 frequency, SE is the standard error and N is the sample size^41,42^. Then we calculated F-statistic to evaluate the instrumental strength of our SNPs for each trait in explaining phenotypic variation using the formula F = [(N − k − 1)/k] × [R^2^/(1 − R^2^)]^43^ where N is the sample size, k is the total number of SNPs that are selected for MR analysis, and R2 is the total proportion of phenotypic variation that is explained by all the SNPs in our MR analysis. An F-statistic > 10 suggests that the combined SNPs is a sufficiently strong instrument to explain phenotypic variation, while a F-statistic ≤ 10 implies a weak instrument^43^.

### Statistical analysis

#### Univariable Mendelian randomization analyses

We undertook a bi-directional MR study to estimate the causal associations between four iron status and sarcopenia-related traits using three MR methods, inverse variance weighted (IVW), MR-Egger, weighted median (WM)^44^. The IVW method uses a meta-analysis approach to combine the Wald ratios of the genetically causal effects of each SNP, relying on the assumption that all SNPs are valid IVs with no evidence of directional pleiotropy^37^. So, it is considered to provide an estimate with the highest power and the best precision and is used as major analysis^45,46^. Considering the acknowledged variances in iron homeostasis across genders^47^, we applied UVMR to extend our inquiry into the correlation between the four iron status indicators and appendicular lean mass (ALM), leveraging available gender-stratified datasets. We calculated a Bonferroni-corrected *p* threshold, by dividing 0.05 by the number of tests, which assumes each test is independent^48–50^. In this study, the Bonferroni-corrected p threshold for both forward and reverse Mendelian randomization analyses are 0.0025(0.05/20). We considered a *p* value less than Bonferroni-corrected *p* threshold as being statistically significant^51^, and that larger than Bonferroni-corrected *p* threshold but less than 0.05 was suggestive of statistical significance in the univariable MR analysis^52^. Odd ratios (ORs) and corresponding 95% confidence intervals (CIs) were calculated for estimating causal effects of iron status on low hand grip strength.

#### Heterogeneity, pleiotropy, and sensitivity analysis

We applied the Pleiotropy RESidual Sum and Outlier (MR-PRESSO) analysis^53^ to provide outlier-adjusted estimates of causal associations. This involved removing one or more pleiotropic outlying SNPs and re-conducting the MR analyses. To detect whether the observed causal estimates were biased by reverse causality, we applied Steiger filtering. Furthermore, in order to assess potential heterogeneity and pleiotropy biases, we conducted heterogeneity, pleiotropy, and sensitivity analyses. The Cochran's Q test was used to evaluate heterogeneity between instrumental variables in the MR, with random-effect models used if the p value of the Cochran's Q test was less than 0.05^54^. We also performed leave-one-out sensitivity analyses to assess the influence of each SNP on the overall MR estimates^55^. If one or more SNPs were found to significantly alter the overall MR estimates, it would be removed and the MR analyses were re-performed. Lastly, we used the MR-Egger intercept method, specifically the *mr_pleiotropy_test* function in R *TwoSampleMR* package, to evaluate the pleiotropy of our effect estimates.

#### MVMR analysis

Given the interrelated nature of the four iron biomarkers established in prior research^33^, it was imperative to conduct MVMR analysis to elucidate the primary drivers behind the causal associations observed between iron-related biomarkers and sarcopenia-related traits. Unlike UVMR analysis, MVMR analysis assumes that the IVs are strongly associated with at least one exposure, although not necessarily with each. In the forward analysis, we excluded TIBC from the subsequent MVMR analysis due to collinearity with other iron-related biomarkers. Instead, we employed a combination of SNPs as an integrated proxy for the three iron-related biomarkers, ensuring convergence in our analysis.

To address potential confounding factors, particularly the reduction in physical activity (PA) associated with anemia in individuals with iron deficiency, we conducted additional analyses utilizing MVMR. Specifically, each of the four iron status indicators was adjusted for physical activity. Details of the physical activity data source are provided in Table 1.

#### Statistic power

Moreover, we used a webpage-based application, the online sample size and power calculator (https://sb452.shinyapps.io/power/), to estimate the statistical power for detecting causal effects of iron status on sarcopenia-related traits^56^. The power calculator uses simulations to estimate the power for a given set of parameters, providing researchers with valuable information for designing MR studies with sufficient statistical power.

#### Statistical tools

All statistical analyses and visualization for results were performed using R statistical software (version 4.2.2, R Foundation for Statistical Computing, Vienna, Austria; https://www.R-project.org) with the *TwoSampleMR, LDlinkR, presso, and forestplot* Packages.

### Ethics statement

Ethical approval and consent to participate in the original genome-wide association studies (GWASs) were obtained from relevant review boards.

## Results

### Instrumental variables for iron status

We obtained 48 sets of SNPs serving as IVs when performing the UVMR analysis (Supplementary Table S1). We calculated the F-values of 48 sets of SNPs and found that they ranged from 38.1 to 521.5 (Tables 2, 3, Supplementary Table S2), which suggests that there is no bias caused by weak instrumental variables in this study.

**Table 2:** Univariable Mendelian randomization estimates of iron status on sarcopenia-related traits.

Exposure	Outcome	IVs	R^2^ (%)	F-statistics	Method	Beta/OR	95% CI	P-value
Ferritin	Appendicular lean mass	33	1.146	86.458	IVW	− 0.051	− 0.072, − 0.031	7.325 × 10^–07^
					MR Egger	− 0.066	− 0.106, − 0.027	0.002
					WM	− 0.053	− 0.082, − 0.024	4.132 × 10^–04^
	Hand grip strength (left)	45	1.343	74.451	IVW	− 0.010	− 0.026, 0.006	0.214
					MR Egger	0.003	− 0.027, 0.032	0.859
					WM	− 0.004	− 0.027, 0.019	0.707
	Hand grip strength (right)	45	1.343	74.451	IVW	− 0.013	− 0.029, 0.002	0.084
					MR Egger	− 0.001	− 0.029, 0.028	0.962
					WM	− 0.007	− 0.028, 0.015	0.541
	Low hand grip strength	45	1.380	76.520	IVW	0.977	0.899, 1.061	0.575
					MR Egger	0.964	0.824, 1.128	0.651
					WM	0.992	0.885, 1.112	0.892
	Walking pace	45	1.343	74.451	IVW	0.002	− 0.011, 0.016	0.727
					MR Egger	− 0.004	− 0.03, 0.022	0.765
					WM	− 2.668 × 10^–04^	− 0.02, 0.02	0.979
Serum iron	Appendicular lean mass	19	0.795	68.963	IVW	0.028	− 0.003, 0.06	0.072
					MR Egger	0.008	− 0.051, 0.068	0.787
					WM	0.044	0.005, 0.084	0.029
	Hand grip strength (left)	24	1.617	111.98	IVW	− 0.015	− 0.03, 0.001	0.061
					MR Egger	− 0.016	− 0.039, 0.008	0.209
					WM	− 0.022	− 0.042, − 0.002	0.028
	Hand grip strength (right)	23	1.596	115.307	IVW	− 0.012	− 0.026, 0.002	0.101
					MR Egger	− 0.023	− 0.044, − 0.001	0.053
					WM	− 0.029	− 0.049, − 0.009	0.004
	Low hand grip strength	23	0.941	67.528	IVW	0.918	0.836, 1.008	0.072
					MR Egger	0.984	0.811, 1.194	0.874
					WM	0.961	0.837, 1.102	0.566
	Walking pace	23	1.596	115.307	IVW	− 0.008	− 0.022, 0.005	0.232
					MR Egger	− 0.002	− 0.022, 0.019	0.883
					WM	− 0.012	− 0.03, 0.006	0.197
TIBC	Appendicular lean mass	16	1.987	171.619	IVW	− 0.020	− 0.037, − 0.002	0.028
					MR Egger	− 0.029	− 0.055, − 0.004	0.038
					WM	− 0.026	− 0.047, − 0.006	0.012
	Hand grip strength (left)	20	2.293	158.877	IVW	− 0.005	− 0.017, 0.007	0.451
					MR Egger	0.009	− 0.009, 0.027	0.339
					WM	− 0.002	− 0.02, 0.015	0.774
	Hand grip strength (right)	22	2.369	149.318	IVW	0.003	− 0.01, 0.015	0.688
					MR Egger	0.013	− 0.006, 0.031	0.189
					WM	0.007	− 0.01, 0.023	0.445
	Low hand grip strength	29	2.585	123.900	IVW	1.013	0.946, 1.085	0.702
					MR Egger	0.970	0.873, 1.078	0.578
					WM	0.983	0.901, 1.073	0.686
	Walking pace	22	2.313	145.718	IVW	0.006	− 0.004, 0.016	0.264
					MR Egger	0.014	− 0.002, 0.029	0.096
					WM	0.006	− 0.008, 0.019	0.409
TSAT	Appendicular lean mass	19	1.823	128.437	IVW	0.022	0.002, 0.043	0.035
					MR Egger	0.017	− 0.015, 0.048	0.316
					WM	0.028	0.006, 0.05	0.011
	Hand grip strength (left)	26	3.229	168.714	IVW	0.006	− 0.005, 0.017	0.305
					MR Egger	0.006	− 0.012, 0.024	0.528
					WM	0.002	− 0.012, 0.015	0.805
	Hand grip strength (right)	26	3.229	168.714	IVW	0.002	− 0.01, 0.013	0.742
					MR Egger	− 0.005	− 0.023, 0.013	0.570
					WM	− 0.006	− 0.02, 0.009	0.421
	Low hand grip strength	26	3.229	168.714	IVW	1.000	0.952, 1.051	0.994
					MR Egger	1.031	0.954, 1.114	0.452
					WM	1.020	0.955, 1.088	0.562
	Walking pace	20	2.094	140.583	IVW	− 0.002	− 0.013, 0.008	0.643
					MR Egger	− 3.133 × 10^–04^	− 0.017, 0.016	0.971
					WM	− 0.002	− 0.016, 0.012	0.759

*WM* weighted median, *IVW* inverse variance weighted, *TIBC* total iron binding capacity, *TSAT* transferrin saturation, *IVs* numbers of instrumental variable, *CI* confidence interval, *OR* odds ratio.

**Table 3:** Univariable Mendelian randomization estimates of sarcopenia-related traits on iron status.

Exposure	Outcome	IVs	R^2^ (%)	F-statistics	Method	Beta	95% CI	P-value
Appendicular lean mass	Ferritin	534	11.326	107.565	IVW	− 0.01	− 0.026, 0.006	0.215
					MR Egger	− 0.017	− 0.054, 0.02	0.372
					WM	− 0.003	− 0.029, 0.023	0.796
	Serum iron	571	12.468	112.178	IVW	− 0.013	− 0.031, 0.004	0.135
					MR Egger	− 0.026	− 0.067, 0.015	0.222
					WM	− 0.028	− 0.056, − 0.001	0.046
	TIBC	555	11.597	106.294	IVW	− 0.031	− 0.052, − 0.01	0.004
					MR Egger	− 0.049	− 0.099, 3.6 × 10^–4^	0.049
					WM	− 0.036	− 0.069, − 0.002	0.037
	TSAT	566	12.453	113.006	IVW	0.002	− 0.018, 0.022	0.827
					MR Egger	0.029	− 0.016, 0.074	0.210
					WM	0.01	− 0.022, 0.042	0.539
Hand grip strength (left)	Ferritin	141	1.396	46.261	IVW	− 0.014	− 0.075, 0.047	0.648
					MR Egger	0.015	− 0.215, 0.244	0.899
					WM	0.02	− 0.068, 0.109	0.653
	Serum iron	129	1.289	46.672	IVW	− 0.087	− 0.156, − 0.018	0.013
					MR Egger	− 0.079	− 0.337, 0.179	0.549
					WM	− 0.056	− 0.156, 0.045	0.279
	TIBC	143	1.444	47.234	IVW	− 0.038	− 0.12, 0.044	0.362
					MR Egger	0.001	− 0.316, 0.318	0.997
					WM	0.012	− 0.101, 0.125	0.838
	TSAT	148	1.492	47.172	IVW	− 0.009	− 0.086, 0.068	0.824
					MR Egger	− 0.088	− 0.377, 0.2	0.549
					WM	− 0.012	− 0.122, 0.099	0.838
Hand grip strength (right)	Ferritin	151	1.502	46.551	IVW	− 0.001	− 0.059, 0.058	0.981
					MR Egger	− 0.091	− 0.313, 0.132	0.425
					WM	0.002	− 0.079, 0.084	0.953
	Serum iron	164	1.659	47.425	IVW	− 0.051	− 0.115, 0.013	0.119
					MR Egger	− 0.031	− 0.264, 0.202	0.796
					WM	− 0.009	− 0.099, 0.081	0.844
	TIBC	158	1.591	47.162	IVW	− 0.05	− 0.128, 0.028	0.211
					MR Egger	− 0.016	− 0.303, 0.27	0.910
					WM	− 0.091	− 0.197, 0.016	0.097
	TSAT	163	1.652	47.504	IVW	0.007	− 0.065, 0.08	0.840
					MR Egger	0.134	− 0.127, 0.395	0.315
					WM	0.056	− 0.046, 0.159	0.283
Low hand grip strength	Ferritin	14	0.208	38.164	IVW	− 0.007	− 0.055, 0.041	0.774
					MR Egger	0.135	1.5 × 10^–4^, 0.271	0.074
					WM	− 0.007	− 0.064, 0.05	0.810
	Serum iron	12	0.188	40.322	IVW	0.026	− 0.025, 0.078	0.315
					MR Egger	0.079	− 0.087, 0.246	0.371
					WM	0.053	− 0.011, 0.116	0.104
	TIBC	11	0.164	38.303	IVW	0.006	− 0.069, 0.081	0.870
					MR Egger	− 0.239	− 0.426, − 0.052	0.034
					WM	− 0.001	− 0.092, 0.091	0.987
	TSAT	15	0.235	40.359	IVW	− 0.029	− 0.088, 0.029	0.325
					MR Egger	0.067	− 0.118, 0.252	0.491
					WM	− 0.028	− 0.1, 0.045	0.457
Walking pace	Ferritin	47	0.391	38.442	IVW	0.076	− 0.058, 0.209	0.267
					MR Egger	− 0.056	− 0.56, 0.449	0.830
					WM	0.154	− 0.028, 0.335	0.097
	Serum iron	50	0.435	40.209	IVW	0.029	− 0.117, 0.175	0.695
					MR Egger	0.215	− 0.37, 0.801	0.475
					WM	0.014	− 0.191, 0.219	0.894
	TIBC	53	0.462	40.315	IVW	− 0.147	− 0.322, 0.028	0.099
					MR Egger	− 0.294	− 1.007, 0.42	0.424
					WM	− 0.231	− 0.465, 0.004	0.054
	TSAT	53	0.457	39.806	IVW	0.017	− 0.155, 0.189	0.849
					MR Egger	0.475	− 0.208, 1.158	0.179
					WM	0.049	− 0.183, 0.281	0.680

*WM* weighted median, *IVW* inverse variance weighted, *TIBC* total iron binding capacity, *TSAT* transferrin saturation, *IVs* numbers of instrumental variable, *CI* confidence interval.

### UVMR analysis

**Figure 2:**
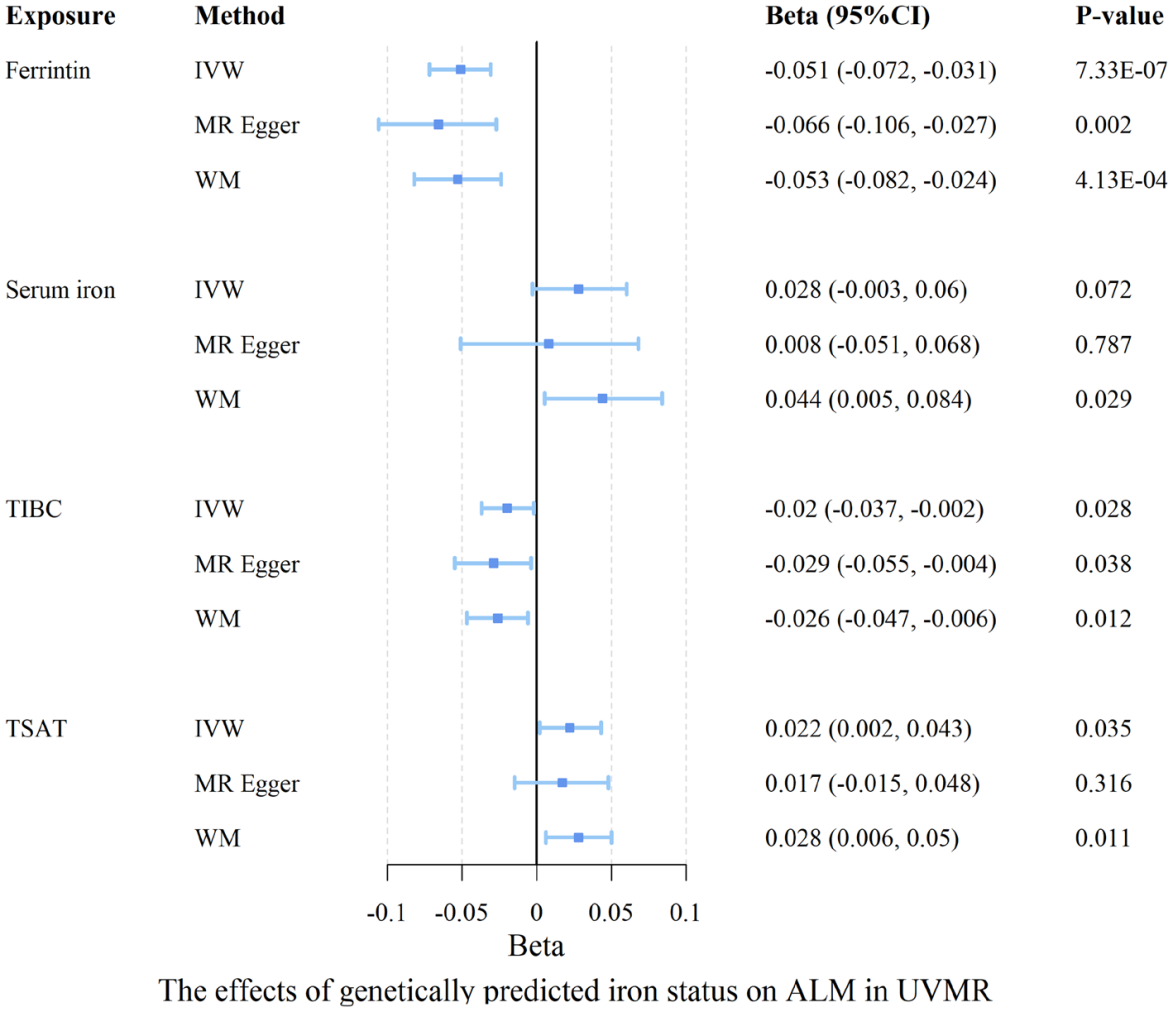
Forest plot for causal associations of iron status with ALM in UVMR analysis. Ferritin, serum iron, TIBC, TSAT are scaled to an SD increase. Effects (beta) represents change in kg ALM. *IVW* inverse variance weighted, *WM* weighted median, *ALM* appendicular lean mass, *TIBC* total iron binding capacity, *TSAT* transferrin saturation, *CI* confidence interval, *UVMR* univariable Mendelian randomization.

As shown in Fig. 2, genetically predicted ferritin has a significant causal effect on appendicular lean mass (β = − 0.051, 95% CI − 0.072, − 0.031, *p* = 7.325 × 10^–07^), indicating that for each standard deviation (SD) increase in ferritin levels, there is an associated decrease in ALM by approximately 0.051 kg. This effect was observed with a high level of statistical power (95.6%). In contrast, no significant causal effects were observed for ferritin on the other four traits of sarcopenia. The associations were still significant after Bonferroni correction (*p* < 0.0025).

For TIBC, our observational analysis revealed a negative association with appendicular lean mass (β = − 0.020, 95% CI − 0.037, − 0.002, *p* = 0.028), though with modest statistical power (47.3%). Our reverse Mendelian randomization study also found a negative TIBC-appendicular lean mass correlation (β = − 0.031, 95% CI − 0.052, − 0.010, *p* = 0.004) with higher power (97.3%). Additionally, TSAT was positively associated with appendicular lean mass (β = 0.022, 95% CI 0.002, 0.043, *p* = 0.035) with 51.3% power, indicating its potential protective role. Notably, the p-values for these associations fell between the Bonferroni-corrected threshold and 0.05, suggesting that additional studies with larger sample sizes are needed to confirm the observed effect.

No significant causal associations were observed between the remaining iron status and sarcopenia-related traits in both the forward and reverse MR analyses. The results regarding causal associations between the four iron-related biomarkers and sarcopenia-related traits by UVMR analyses based on three MR methods are demonstrated in Tables 2 and 3. Upon stratifying the dataset by gender, our investigation revealed no statistically significant associations between four iron status indicators and ALM (Supplementary Table S2).

### Heterogeneity, pleiotropy, and sensitivity analysis

We obtained estimates that were consistent with our original results after removing outliers detected by MR-PRESSO analyses, demonstrating the stability of our findings after correcting for the presence of pleiotropic effects. Our investigations employing steiger filtering revealed no presence of reverse causation among the examined SNPs, ensuring the reliability of the inferred causal direction. In addition, we evaluated the potential impact of pleiotropy by utilizing the MR-Egger intercept, which revealed no indication of any such influence on our estimates. However, we noted moderate heterogeneity in the analysis of low hand grip strength by TIBC. Moreover, in our gender-stratified analyses, heterogeneity was observed in several associations: Serum Iron (female) and TIBC (female) with ALM (female), as well as TSAT (female) with ALM (female). Additionally, heterogeneity was evident in the analysis of ferritin (male) with ALM (male) (Supplementary Tables S3–S5). Furthermore, our leave-one-out sensitivity analyses did not reveal any significant changes in effect estimates when any one SNP was removed (Supplementary Figs. S1–S47), suggesting that our findings were not driven by any one particular SNP.

### MVMR analysis

**Figure 3:**
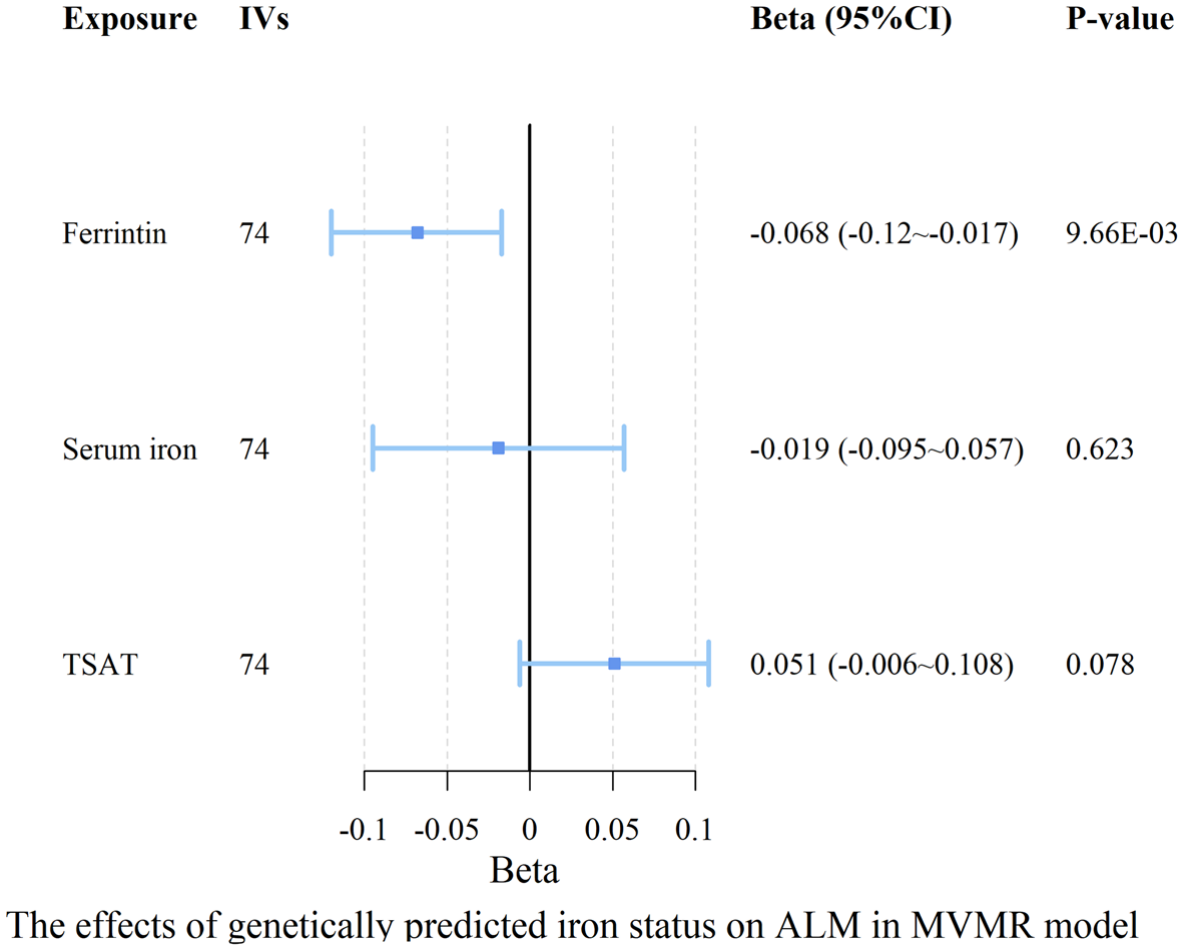
Forest plot for causal associations of iron status with ALM in MVMR analysis. Ferritin, serum iron, TSAT are scaled to an SD increase. Effects (beta) represents change in kg ALM. *ALM* appendicular lean mass, *TSAT* transferrin saturation, *IVs* numbers of instrumental variable, *CI* confidence interval, *MVMR* multivariable Mendelian randomization.

The MVMR analyses using the IVW method demonstrated a significant inverse association between higher ferritin levels and ALM (β = − 0.068, 95% CI − 0.12, − 0.017, p = 9.658 × 10–03), as illustrated in Fig. 3 and Table 4. Interestingly, this effect appears to be the predominant driver of the associations observed between iron status and sarcopenia-related traits, as adjustment for serum iron (β = − 0.019, 95% CI − 0.095, 0.057, *p* = 0.623) and TSAT (β = 0.051, 95% CI − 0.006, 0.108, *p* = 0.078) had negligible impact on the observed effect. Consistent with our UVMR findings, no significant associations were found between iron status and hand grip strength (left or right), low hand grip strength, or walking pace in our MVMR analyses.

**Table 4:** Causal effects of multiple iron status on sarcopenia-related traits based on IVW MVMR model.

Outcome	Exposure	IVs	Beta/OR	95% CI	P-value
Appendicular lean mass	Ferritin	74	− 0.068	− 0.12, − 0.017	9.658 × 10^–03^
	Serum iron	74	− 0.019	− 0.095, 0.057	0.623
	TSAT	74	0.051	− 0.006, 0.108	0.078
Hand grip strength (left)	Ferritin	72	− 0.014	− 0.032, 0.004	0.129
	Serum iron	72	− 0.002	− 0.028, 0.024	0.864
	TSAT	72	0.017	− 0.003, 0.036	0.090
Hand grip strength (right)	Ferritin	72	− 0.014	− 0.032, 0.003	0.108
	Serum iron	72	0.007	− 0.018, 0.033	0.576
	TSAT	72	0.011	− 0.008, 0.03	0.260
Low hand grip strength	Ferritin	74	0.986	0.901, 1.079	0.757
	Serum iron	74	0.972	0.852, 1.109	0.676
	TSAT	74	0.994	0.902, 1.097	0.909
Walking pace	Ferritin	72	− 0.003	− 0.019, 0.013	0.682
	Serum iron	72	− 0.001	− 0.025, 0.022	0.901
	TSAT	72	− 0.004	− 0.021, 0.013	0.651

*TSAT* transferrin saturation, *IVs* numbers of instrumental variable, *CI* confidence interval, *OR* odds ratio.

After adjusting for PA, the analysis revealed that each SD higher genetically predicted ferritin was associated with lower ALM (β = − 0.054, 95% CI − 0.092, − 0.015, *p* = 0.006). However, TIBC (β = 0.033, 95% CI 0.072, 0.005, *p* = 0.090) and TSAT (β = 0.011, 95% CI − 0.008, 0.03, *p* = 0.262), which demonstrated suggestive statistical significance with ALM in UVMR analysis, did not exhibit significance with ALM after correction for the PA (Supplementary Table S6). All the data used in MVMR analysis are detailed in Supplementary Table S7–S11.

## Discussion

In this study, we employed a comprehensive analytical approach to investigate the relationship between iron status and sarcopenia. By analyzing pooled data from genome-wide association studies (GWAS) conducted on European populations, our study aimed to establish a causal relationship between iron status and sarcopenia-related traits. While our sex-subgroup analysis did not reveal any association between the four iron status indicators and ALM, our analysis of the overall dataset, conducted through UVMR and MVMR, consistently demonstrated that genetically predicted serum ferritin levels exerted a significant causal effect on ALM.

Our UVMR and MVMR analyses provide evidence that increased serum ferritin levels may have a detrimental causal effect on ALM. This finding is in line with previous observational studies showing iron overload associates with adverse muscle outcomes^26,57,58^. About 30% of the body's iron is stored in the form of ferritin or hemosiderin, so serum ferritin is a good indicator of the body's iron reserves^59^. High ferritin levels indicate iron overload and saturation of transferrin, allowing non-transferrin bound iron to accumulate and catalyse reactive oxygen species generation. Oxygen-free radicals could cause mitochondrial RNA peroxidation, which further induces the opening of mitochondrial permeability transition pores (mPTP), leads to cytochrome C release into the cytoplasm, caspase-3 activation, and finally skeletal muscle cell apoptosis^60–62^. In addition, some researchers have found that iron overload may affect the function of muscle satellite cells through ferroptosis, affecting the repair of damaged skeletal muscle^63^. Additionally, animal research showed that with increase in iron load in skeletal muscles, skeletal muscle mass decreased while muscle cells were atrophied, Akt-FOXO3 was activated, and atrogin-1 and MuRF1 (ubiquitination marker genes associated with muscle cell atrophy) levels were upregulated^64^. All of the above underlying mechanisms could explain the relationship between serum ferritin and skeletal muscle mass in the extremities. The lack of associations for handgrip strength, walking pace and ferritin also implies that higher ferritin may preferentially induce muscle mass loss rather than strength or physical performance decline.

We observed that TIBC exhibited a potential risk association with sarcopenia, leading to a negative impact on appendicular lean mass. On the other hand, our reverse MR result indicates that decreased appendicular lean mass could elevate TIBC. One possible explanation is that under normal circumstances, ferritin can be disassembled by autophagy, releasing iron for cellular processes^65^. Autophagy is impaired in skeletal muscles with aging^66^. Inappropriate sequestration of iron into ferritin, or a failing in the breakdown of ferritin that ultimately reduces the available free iron in the cell, causing functional iron deficiency, which then affects the normal energy metabolism of skeletal muscle, leading to skeletal muscle atrophy^3^. The above underlying mechanisms could similarly explain the significant association of reduced TSAT with the decrease in appendicular lean mass. This is because elevated serum ferritin associated with decreased TSAT is often typical of functional iron deficiency. Notably, under the Bonferroni correction significance level, no correlation was observed between TIBC and TSAT with appendicular lean mass. These findings imply that future investigations should include larger GWAS datasets and consider conducting meta-analyses using data from multiple sources to provide further insights into the relationship between these variables.

Interestingly, no significant associations were found between serum iron and sarcopenia traits in our study. To date, limited research has been conducted to investigate this particular relationship. Bartali et al.^67^ conducted a longitudinal study involving 698 participants but failed to identify a significant link between serum iron levels and physical function. Similarly, a prior systematic review^68^ also failed to demonstrate a significant relationship between serum iron and sarcopenia. A reason could be that these markers reflect iron availability in the short term, while ferritin indicates long-term iron storage and may better predict chronic health risks.

The present study possesses several notable strengths. To the best of our knowledge, this study represents the first attempt to explore the causal associations between iron status and sarcopenia using Mendelian randomization, leveraging large-scale genome-wide association study (GWAS) data. The implementation of MR design stands as a significant strength, as it effectively mitigates residual confounding and other biases, thereby enhancing the strength of causal inferences drawn^69^. Our employment of UVMR and MVMR analyses surpasses previous observational studies, as we have leveraged summary data derived from GWASs featuring an extensive sample size and a vast number of SNPs. Furthermore, the outcomes obtained are characterized by robustness and reliability, demonstrated by the absence of heterogeneity or pleiotropic effects.

However, several limitations are inherent in our study. Primarily, the genetic variant data primarily relied upon GWASs conducted on individuals of European descent, which may restrict the generalizability of our findings to the broader population. Nonetheless, the restriction of participant descent serves to minimize the potential confounding effects stemming from population admixture. Secondly, it is important to note that while efforts were made to calculate type 1 error rates below 0.05, the possibility of weak instrumental variable bias resulting from sample overlap could not be entirely eliminated, as both exposure and outcome data were partially obtained from UKBs. Lastly, iron deficiency and iron overload may have distinct effects on muscle mass, and understanding these differences is vital for accurate interpretation. However, the inability to assess non-linear relationships hampers our understanding of the underlying mechanisms driving the observed associations. The use of summary-level data limits our ability to capture potential threshold or saturation effects, as it only allows for the estimation of average linear causal effects.

## Conclusion

In summary, our MR study offers novel insights into the potential role of elevated serum ferritin as a causal factor associated with decreased appendicular lean mass. While our results might suggest that strategies to reduce ferritin levels could potentially influence muscle atrophy, it is important to note that these findings are preliminary. Obtaining more detailed data in the future is necessary to conduct nonlinear analyses and elucidate the relationship between iron status and sarcopenia further.

### Supplementary Information

Supplementary Figures.

Supplementary Tables.

## Data Availability

The datasets analyzed in this study are publicly available summary statistics. Summary statistics for the GWASs concerning the exposures and outcome are available from the decode genetics (https://www.decode.com/summarydata/) and UK Biobank (https://www.nealelab.is/uk-biobank). For the datasets used and/or analyzed, and the codes used during the current study, please contact the corresponding author at Zgy996600@163.com (Guo-yang Zhao) on reasonable request.

## References

[1] Cruz-Jentoft AJ, Sayer AA (2019). Sarcopenia. Lancet.

[2] Chen LK, Liu LK, Woo J, Assantachai P, Auyeung TW, Bahyah KS (2014). Sarcopenia in Asia: consensus report of the Asian Working Group for Sarcopenia. J. Am. Med. Dir. Assoc..

[3] Alves FM, Ayton S, Bush AI, Lynch GS, Koopman R (2023). Age-related changes in skeletal muscle iron homeostasis. J. Gerontol. Ser. A.

[4] Mijnarends DM, Luiking YC, Halfens RJG, Evers S, Lenaerts ELA, Verlaan S (2018). Muscle, health and costs: A glance at their relationship. J. Nutr. Health Aging.

[5] Cao L, Morley JE (2016). Sarcopenia is recognized as an independent condition by an international classification of disease, tenth revision, clinical modification (ICD-10-CM) code. J. Am. Med. Dir. Assoc..

[6] Koller M (2023). Sarcopenia-a geriatric pandemic: A narrative review. Wien Med Wochenschr..

[7] Muckenthaler MU, Rivella S, Hentze MW, Galy B (2017). A red carpet for iron metabolism. Cell.

[8] Beard JL (2001). Iron biology in immune function, muscle metabolism and neuronal functioning. J. Nutr..

[9] Zheng Q, Guan Y, Xia L, Wang Z, Jiang Y, Zhang X (2016). Effect of Yi Gong San decoction on iron homeostasis in a mouse model of acute inflammation. Evid. Based Complement. Altern. Med..

[10] Ganz T (2013). Systemic iron homeostasis. Physiol. Rev..

[11] Roemhild K, von Maltzahn F, Weiskirchen R, Knuchel R, von Stillfried S, Lammers T (2021). Iron metabolism: pathophysiology and pharmacology. Trends Pharmacol. Sci..

[12] Gattermann N, Muckenthaler MU, Kulozik AE, Metzgeroth G, Hastka J (2021). The evaluation of iron deficiency and iron overload. Dtsch Arztebl Int..

[13] Doehner W, Scherbakov N, Schellenberg T, Jankowska EA, Scheitz JF, von Haehling S (2022). Iron deficiency is related to low functional outcome in patients at early rehabilitation after acute stroke. J. Cachexia Sarcopenia Muscle.

[14] Gattermann N (2009). The treatment of secondary hemochromatosis. Dtsch. Arztebl. Int..

[15] Kadoglou NPE, Biddulph JP, Rafnsson SB, Trivella M, Nihoyannopoulos P, Demakakos P (2017). The association of ferritin with cardiovascular and all-cause mortality in community-dwellers: The English longitudinal study of ageing. PLoS ONE.

[16] Mena NP, Urrutia PJ, Lourido F, Carrasco CM, Nunez MT (2015). Mitochondrial iron homeostasis and its dysfunctions in neurodegenerative disorders. Mitochondrion.

[17] Li GF, Pan YZ, Sirois P, Li K, Xu YJ (2012). Iron homeostasis in osteoporosis and its clinical implications. Osteoporos. Int..

[18] Valerio LG, Parks T, Petersen DR (1996). Alcohol mediates increases in hepatic and serum nonheme iron stores in a rat model for alcohol-induced liver injury. Alcohol Clin. Exp. Res..

[19] Zeidan RS, Han SM, Leeuwenburgh C, Xiao R (2021). Iron homeostasis and organismal aging. Ageing Res. Rev..

[20] Sze SCW, Zhang L, Zhang S, Lin K, Ng TB, Ng ML (2022). Aberrant transferrin and ferritin upregulation elicits iron accumulation and oxidative inflammaging causing ferroptosis and undermines estradiol biosynthesis in aging rat ovaries by upregulating NF-Kappab-activated inducible nitric oxide synthase: First demonstration of an intricate mechanism. Int. J. Mol. Sci..

[21] Jung SH, DeRuisseau LR, Kavazis AN, DeRuisseau KC (2008). Plantaris muscle of aged rats demonstrates iron accumulation and altered expression of iron regulation proteins. Exp. Physiol..

[22] Labranche R, Gilbert G, Cerny M, Vu KN, Soulieres D, Olivie D (2018). Liver iron quantification with MR imaging: A primer for radiologists. Radiographics.

[23] Halon-Golabek M, Borkowska A, Herman-Antosiewicz A, Antosiewicz J (2019). Iron metabolism of the skeletal muscle and neurodegeneration. Front. Neurosci..

[24] Xu B, Guo Z, Jiang B, Zhang K, Zhu W, Lian X (2022). Factors affecting sarcopenia in older patients with chronic diseases. Ann. Palliat. Med..

[25] Ho V, Lee CT, Merchant RA (2022). The "Iron Tale"- iron indices and handgrip strength in community-dwelling adults. Aging Clin. Exp. Res..

[26] Nakagawa C, Inaba M, Ishimura E, Yamakawa T, Shoji S, Okuno S (2016). Association of increased serum ferritin with impaired muscle strength/quality in hemodialysis patients. J. Renal Nutr..

[27] Davey Smith G, Hemani G (2014). Mendelian randomization: Genetic anchors for causal inference in epidemiological studies. Hum. Mol. Genet..

[28] Davies NM, Holmes MV, Davey SG (2018). Reading Mendelian randomisation studies: A guide, glossary, and checklist for clinicians. BMJ.

[29] Smith GD, Ebrahim S (2003). 'Mendelian randomization': can genetic epidemiology contribute to understanding environmental determinants of disease?. Int. J. Epidemiol..

[30] Burgess S, Small DS, Thompson SG (2017). A review of instrumental variable estimators for Mendelian randomization. Stat. Methods Med. Res..

[31] Skrivankova VW, Richmond RC, Woolf BAR, Yarmolinsky J, Davies NM, Swanson SA (2021). Strengthening the reporting of observational studies in epidemiology using Mendelian randomization: The STROBE-MR statement. Jama.

[32] Zheng J, Baird D, Borges MC, Bowden J, Hemani G, Haycock P (2017). Recent developments in Mendelian randomization studies. Curr. Epidemiol. Rep..

[33] Bell S, Rigas AS, Magnusson MK, Ferkingstad E, Allara E, Bjornsdottir G (2021). A genome-wide meta-analysis yields 46 new loci associating with biomarkers of iron homeostasis. Commun. Biol..

[34] Burgess S, Davies NM, Thompson SG (2016). Bias due to participant overlap in two-sample Mendelian randomization. Genet. Epidemiol..

[35] Li M, Lin J, Liang S, Chen Z, Bai Y, Long X (2021). The role of age at menarche and age at menopause in Alzheimer's disease: Evidence from a bidirectional mendelian randomization study. Aging.

[36] Shen J, Zhou H, Liu J, Zhang Y, Zhou T, Yang Y (2021). A modifiable risk factors atlas of lung cancer: A Mendelian randomization study. Cancer Med..

[37] Cui Z, Hou G, Meng X, Feng H, He B, Tian Y (2020). Bidirectional causal associations between inflammatory bowel disease and ankylosing spondylitis: A two-sample mendelian randomization analysis. Front. Genet..

[38] Hartwig FP, Davies NM, Hemani G, Davey SG (2016). Two-sample Mendelian randomization: avoiding the downsides of a powerful, widely applicable but potentially fallible technique. Int. J. Epidemiol..

[39] Kamat MA, Blackshaw JA, Young R, Surendran P, Burgess S, Danesh J (2019). PhenoScanner V2: an expanded tool for searching human genotype-phenotype associations. Bioinformatics.

[40] Staley JR, Blackshaw J, Kamat MA, Ellis S, Surendran P, Sun BB (2016). PhenoScanner: A database of human genotype-phenotype associations. Bioinformatics.

[41] Palmer TM, Lawlor DA, Harbord RM, Sheehan NA, Tobias JH, Timpson NJ (2012). Using multiple genetic variants as instrumental variables for modifiable risk factors. Stat. Methods Med. Res..

[42] Teslovich TM, Musunuru K, Smith AV, Edmondson AC, Stylianou IM, Koseki M (2010). Biological, clinical and population relevance of 95 loci for blood lipids. Nature.

[43] Burgess S, Thompson SG (2011). Avoiding bias from weak instruments in Mendelian randomization studies. Int. J. Epidemiol..

[44] Marouli E, Del Greco MF, Astley CM, Yang J, Ahmad S, Berndt SI (2019). Mendelian randomisation analyses find pulmonary factors mediate the effect of height on coronary artery disease. Commun. Biol..

[45] Yuan S, Tang B, Zheng J, Larsson SC (2020). Circulating lipoprotein lipids, apolipoproteins and ischemic stroke. Ann. Neurol..

[46] Yin KJ, Huang JX, Wang P, Yang XK, Tao SS, Li HM (2022). No genetic causal association between periodontitis and arthritis: A bidirectional two-sample mendelian randomization analysis. Front Immunol..

[47] Rushton DH, Barth JH (2010). What is the evidence for gender differences in ferritin and haemoglobin?. Crit. Rev. Oncol./Hematol..

[48] Magnus MC, Guyatt AL, Lawn RB, Wyss AB, Trajanoska K, Küpers LK (2020). Identifying potential causal effects of age at menarche: A Mendelian randomization phenome-wide association study. BMC Med..

[49] Millard LAC, Munafò MR, Tilling K, Wootton RE, Davey Smith G (2019). MR-pheWAS with stratification and interaction: Searching for the causal effects of smoking heaviness identified an effect on facial aging. PLoS Genet..

[50] Arathimos R, Millard LAC, Bell JA, Relton CL, Suderman M (2020). Impact of sex hormone-binding globulin on the human phenome. Hum. Mol. Genet..

[51] Wu F, Huang Y, Hu J, Shao Z (2020). Mendelian randomization study of inflammatory bowel disease and bone mineral density. BMC Med..

[52] Went M, Cornish AJ, Law PJ, Kinnersley B, van Duin M, Weinhold N (2020). Search for multiple myeloma risk factors using Mendelian randomization. Blood Adv..

[53] Verbanck M, Chen CY, Neale B, Do R (2018). Detection of widespread horizontal pleiotropy in causal relationships inferred from Mendelian randomization between complex traits and diseases. Nat. Genet..

[54] Greco MF, Minelli C, Sheehan NA, Thompson JR (2015). Detecting pleiotropy in Mendelian randomisation studies with summary data and a continuous outcome. Stat. Med..

[55] Burgess S, Thompson SG (2017). Interpreting findings from Mendelian randomization using the MR-Egger method. Eur. J. Epidemiol..

[56] Brion MJ, Shakhbazov K, Visscher PM (2013). Calculating statistical power in Mendelian randomization studies. Int. J. Epidemiol..

[57] Kim TH, Hwang HJ, Kim SH (2014). Relationship between serum ferritin levels and sarcopenia in Korean females aged 60 years and older using the fourth Korea National Health and Nutrition Examination Survey (KNHANES IV-2, 3), 2008–2009. PLoS ONE.

[58] Perna S, Peroni G, Faliva MA, Bartolo A, Naso M, Miccono A (2017). Sarcopenia and sarcopenic obesity in comparison: prevalence, metabolic profile, and key differences. A cross-sectional study in Italian hospitalized elderly. Aging Clin. Exp. Res..

[59] Cook JD, Flowers CH, Skikne BS (2003). The quantitative assessment of body iron. Blood.

[60] Veatch JR, McMurray MA, Nelson ZW, Gottschling DE (2009). Mitochondrial dysfunction leads to nuclear genome instability via an iron-sulfur cluster defect. Cell.

[61] Liang LP, Jarrett SG, Patel M (2008). Chelation of mitochondrial iron prevents seizure-induced mitochondrial dysfunction and neuronal injury. J. Neurosci..

[62] Duvigneau JC, Piskernik C, Haindl S, Kloesch B, Hartl RT, Hüttemann M (2008). A novel endotoxin-induced pathway: upregulation of heme oxygenase 1, accumulation of free iron, and free iron-mediated mitochondrial dysfunction. Lab. Investig..

[63] Wang Y, Zhang Z, Jiao W, Wang Y, Wang X, Zhao Y (2022). Ferroptosis and its role in skeletal muscle diseases. Front. Mol. Biosci..

[64] Ikeda Y, Imao M, Satoh A, Watanabe H, Hamano H, Horinouchi Y (2016). Iron-induced skeletal muscle atrophy involves an Akt-forkhead box O3–E3 ubiquitin ligase-dependent pathway. J. Trace Elem. Med. Biol..

[65] Radisky DC, Kaplan J (1998). Iron in cytosolic ferritin can be recycled through lysosomal degradation in human fibroblasts. Biochem. J..

[66] Carnio S, LoVerso F, Baraibar MA, Longa E, Khan MM, Maffei M (2014). Autophagy impairment in muscle induces neuromuscular junction degeneration and precocious aging. Cell Rep..

[67] Bartali B, Frongillo EA, Guralnik JM, Stipanuk MH, Allore HG, Cherubini A (2008). Serum micronutrient concentrations and decline in physical function among older persons. Jama.

[68] van Dronkelaar C, van Velzen A, Abdelrazek M, van der Steen A, Weijs PJM, Tieland M (2018). Minerals and sarcopenia; the role of calcium, iron, magnesium, phosphorus, potassium, selenium, sodium, and zinc on muscle mass, muscle strength, and physical performance in older adults: A systematic review. J. Am. Med. Dir. Assoc..

[69] Smit RA, Trompet S, de Craen AJ, Jukema JW (2014). Using genetic variation for establishing causality of cardiovascular risk factors: overcoming confounding and reverse causality. Neth. Heart J..

