# Supplementary material for: Iron status and sarcopenia-related traits: a bi-directional Mendelian randomization study
Source: Sci Rep. 2024 Apr 22;14:9179. doi: 10.1038/s41598-024-60059-w (PMC11035655; doi:10.1038/s41598-024-60059-w)
Supplement: Supplementary file 1 — Supplementary Figures. [file 41598_2024_60059_MOESM1_ESM.pdf]

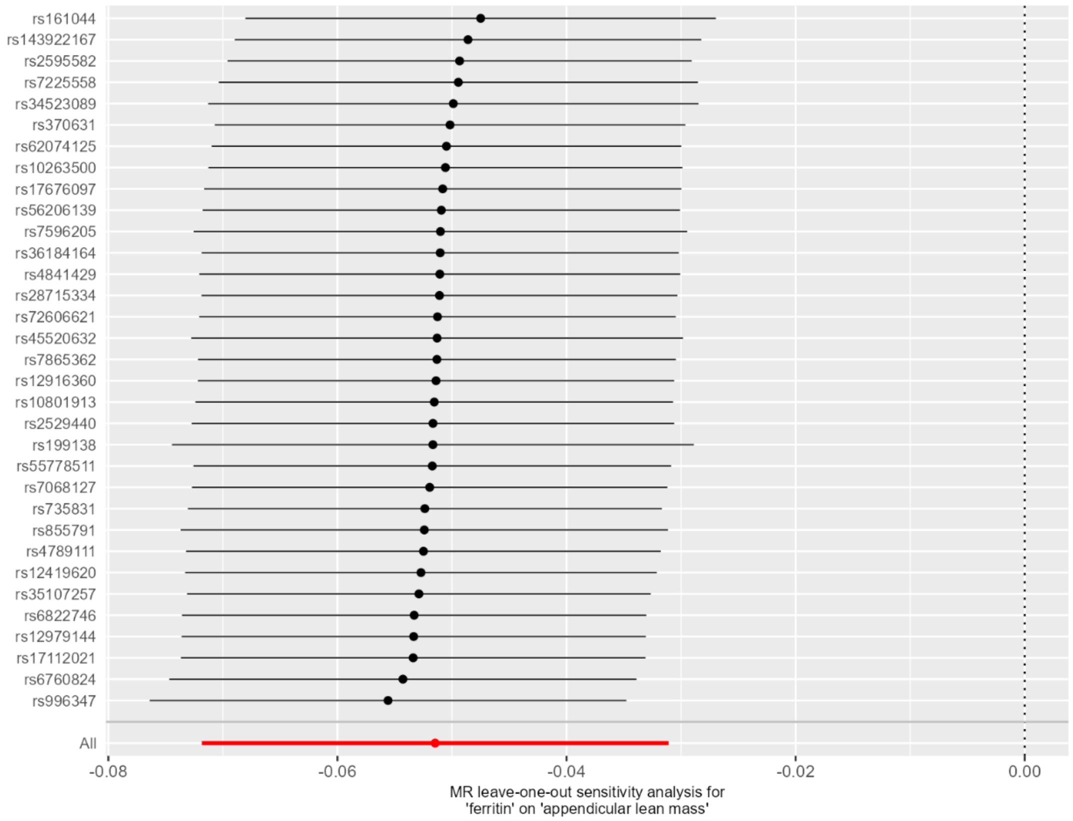

Supplementary Figure 1. Leave-one-out plot for sensitivity analysis of single SNP effect on "ferritin"-to-"appendicular lean mass" UVMR results.

Leave-one-out plot using IVW method by sequentially re-evaluating the causal estimate after discarding one SNP at a time, which helps determine whether the overall effect is driven by the specific genetic variant. The black point denotes the causal effect estimate of ferritin on appendicular lean mass after discarding a certain SNP, and the black line signifies the 95% CI of estimate. The red point symbolizes the causal effect estimate of ferritin on appendicular lean mass, and the red line indicates the 95% CI of the estimate. **Abbreviations:** SNP = number of single-nucleotide polymorphism; UVMR = univariate Mendelian randomization; CI = confidence interval.

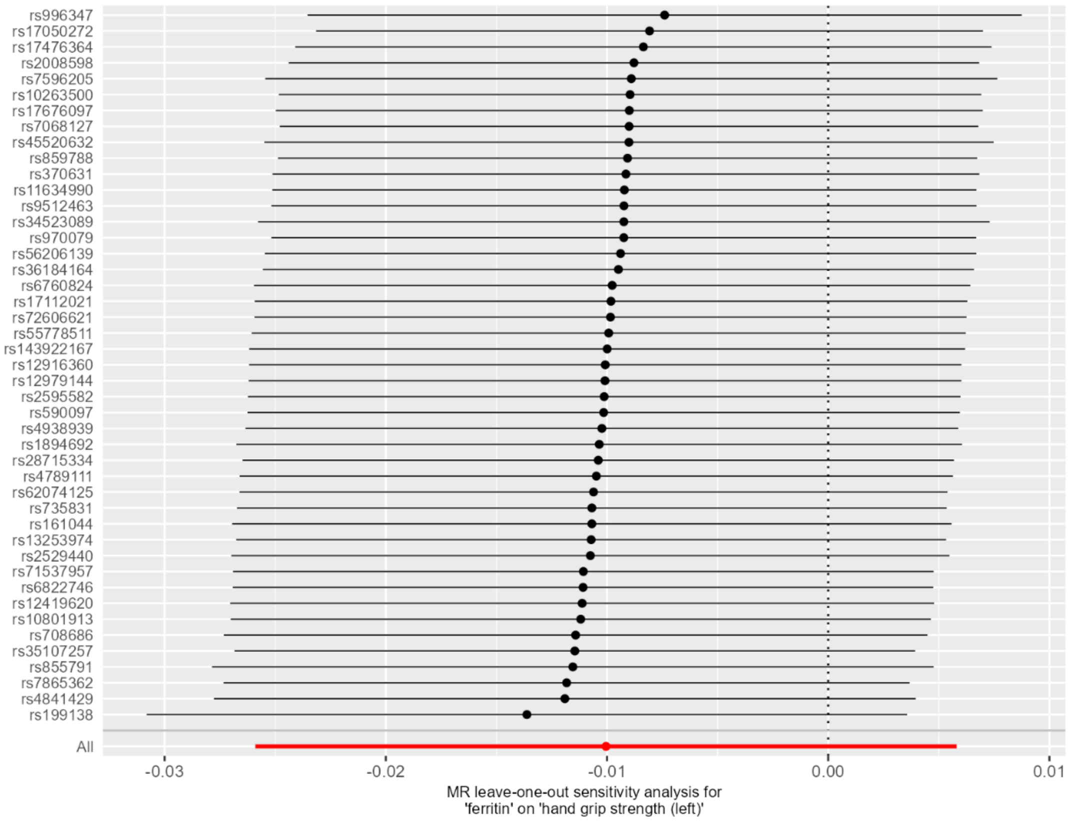

Supplementary Figure 2. Leave-one-out plot for sensitivity analysis of single SNP effect on "ferritin"-to-"hand grip strength (left)" UVMR results.

Leave-one-out plot using IVW method by sequentially re-evaluating the causal estimate after discarding one SNP at a time, which helps determine whether the overall effect is driven by the specific genetic variant. The black point denotes the causal effect estimate of ferritin on hand grip strength (left) after discarding a certain SNP, and the black line signifies the 95% CI of estimate. The red point symbolizes the causal effect estimate of ferritin on hand grip strength (left), and the red line indicates the 95% CI of the estimate. **Abbreviations:** SNP = number of single-nucleotide polymorphism; UVMR = univariate Mendelian randomization; CI = confidence interval.

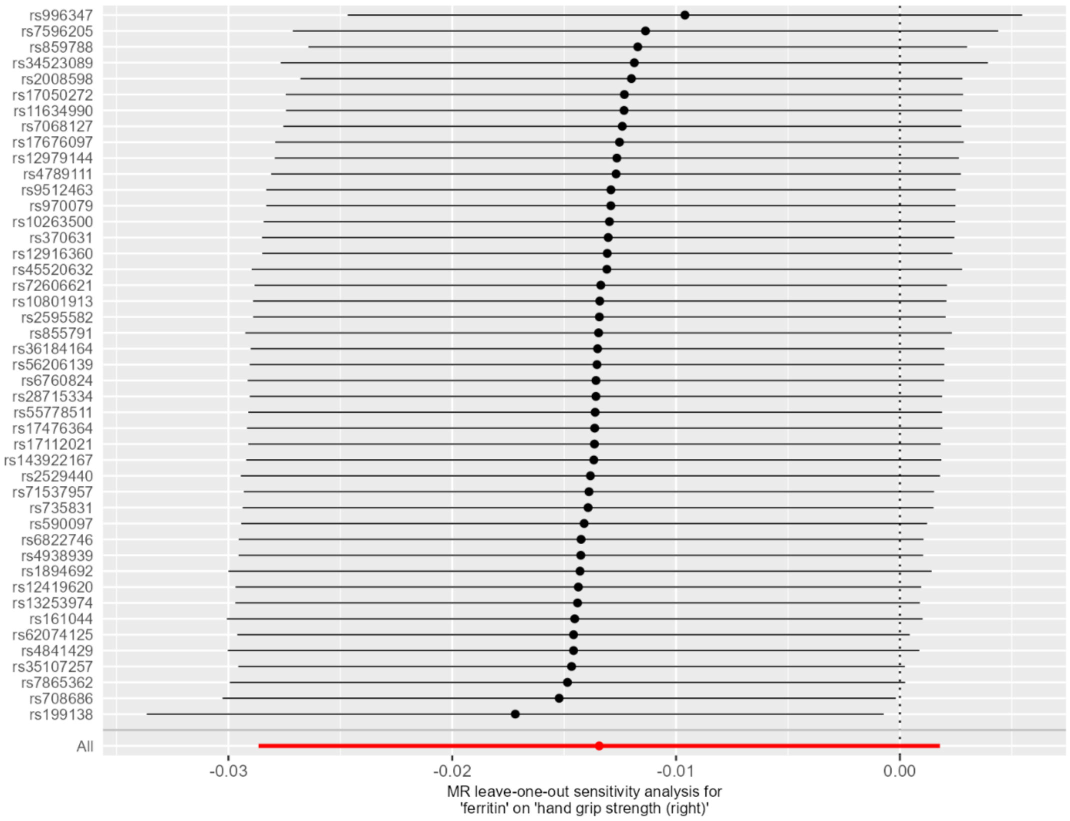

Supplementary Figure 3. Leave-one-out plot for sensitivity analysis of single SNP effect on "ferritin"-to-"hand grip strength (right)" UVMR results.

Leave-one-out plot using IVW method by sequentially re-evaluating the causal estimate after discarding one SNP at a time, which helps determine whether the overall effect is driven by the specific genetic variant. The black point denotes the causal effect estimate of ferritin on hand grip strength (right) after discarding a certain SNP, and the black line signifies the 95% CI of estimate. The red point symbolizes the causal effect estimate of ferritin on hand grip strength (right), and the red line indicates the 95% CI of the estimate. **Abbreviations:** SNP = number of single-nucleotide polymorphism; UVMR = univariate Mendelian randomization; CI = confidence interval.

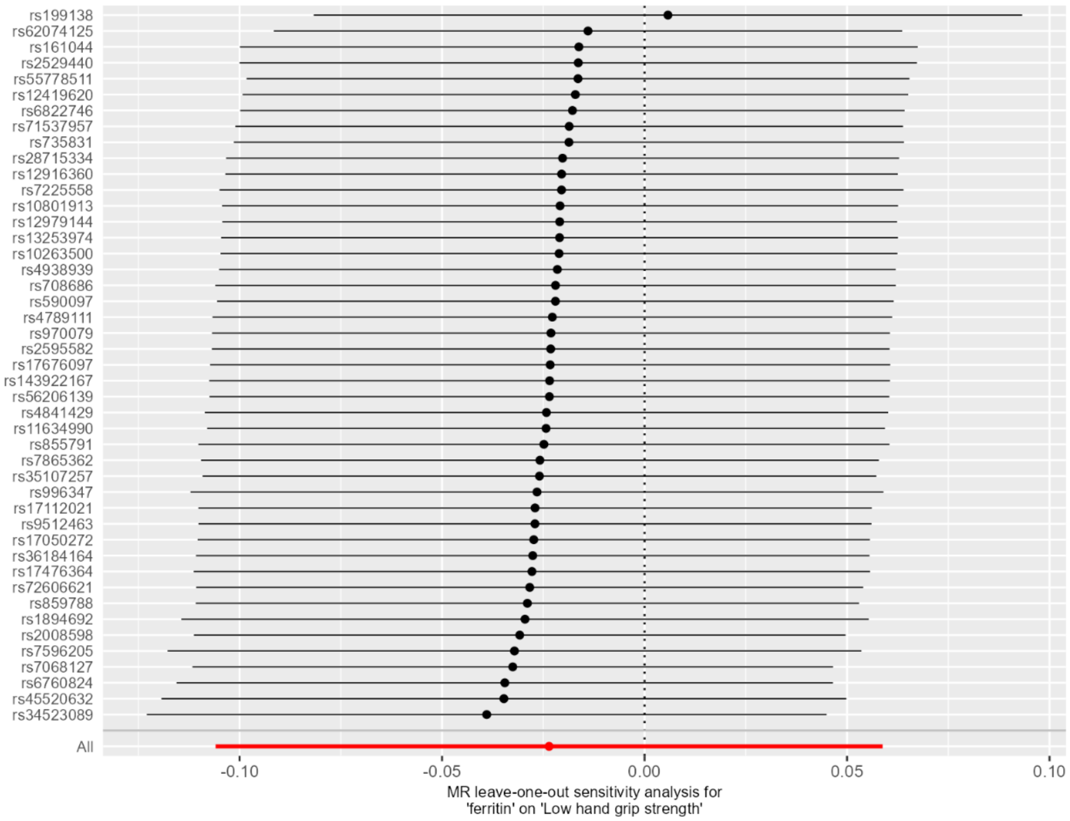

Supplementary Figure 4. Leave-one-out plot for sensitivity analysis of single SNP effect on "ferritin"-to-"low hand grip strength" UVMR results.

Leave-one-out plot using IVW method by sequentially re-evaluating the causal estimate after discarding one SNP at a time, which helps determine whether the overall effect is driven by the specific genetic variant. The black point denotes the causal effect estimate of ferritin on low hand grip strength after discarding a certain SNP, and the black line signifies the 95% CI of estimate. The red point symbolizes the causal effect estimate of ferritin on low hand grip strength, and the red line indicates the 95% CI of the estimate. **Abbreviations:** SNP = number of single-nucleotide polymorphism; UVMR = univariate Mendelian randomization; CI = confidence interval.

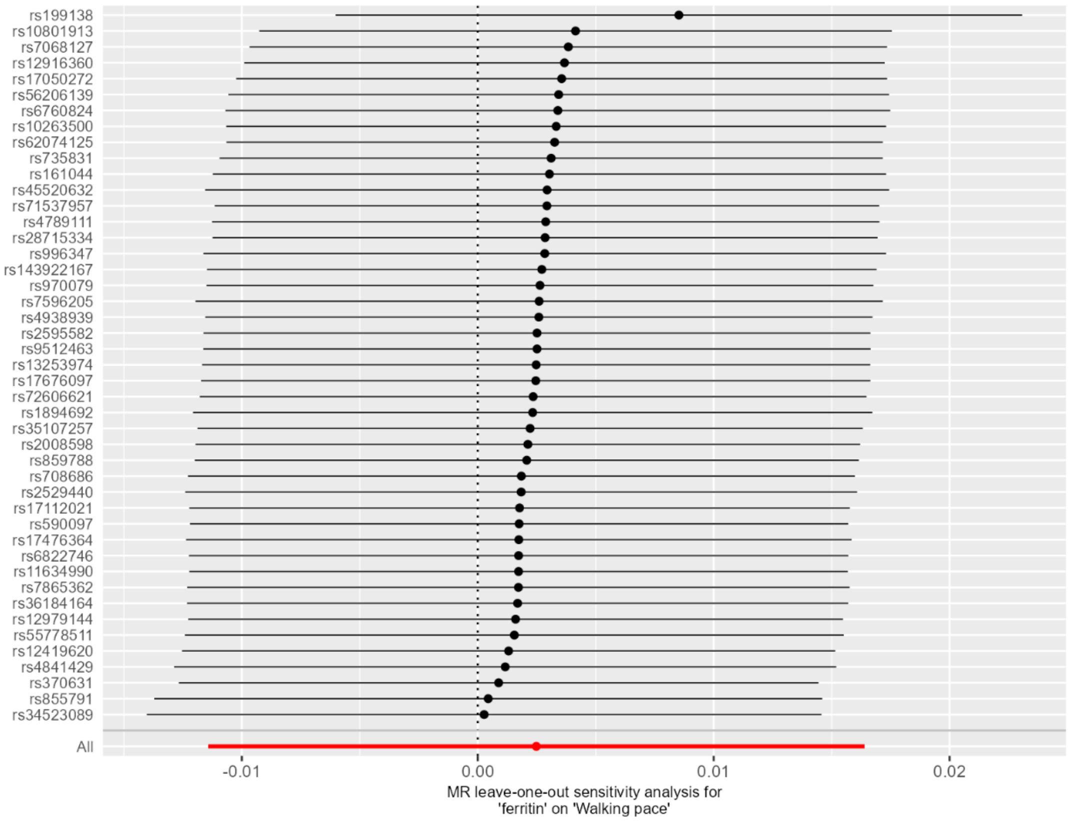

Supplementary Figure 5. Leave-one-out plot for sensitivity analysis of single SNP effect on "ferritin"-to-"walking pace" UVMR results.

Leave-one-out plot using IVW method by sequentially re-evaluating the causal estimate after discarding one SNP at a time, which helps determine whether the overall effect is driven by the specific genetic variant. The black point denotes the causal effect estimate of ferritin on walking pace after discarding a certain SNP, and the black line signifies the 95% CI of estimate. The red point symbolizes the causal effect estimate of ferritin on walking pace, and the red line indicates the 95% CI of the estimate. **Abbreviations:** SNP = number of single-nucleotide polymorphism; UVMR = univariate Mendelian randomization; CI = confidence interval.

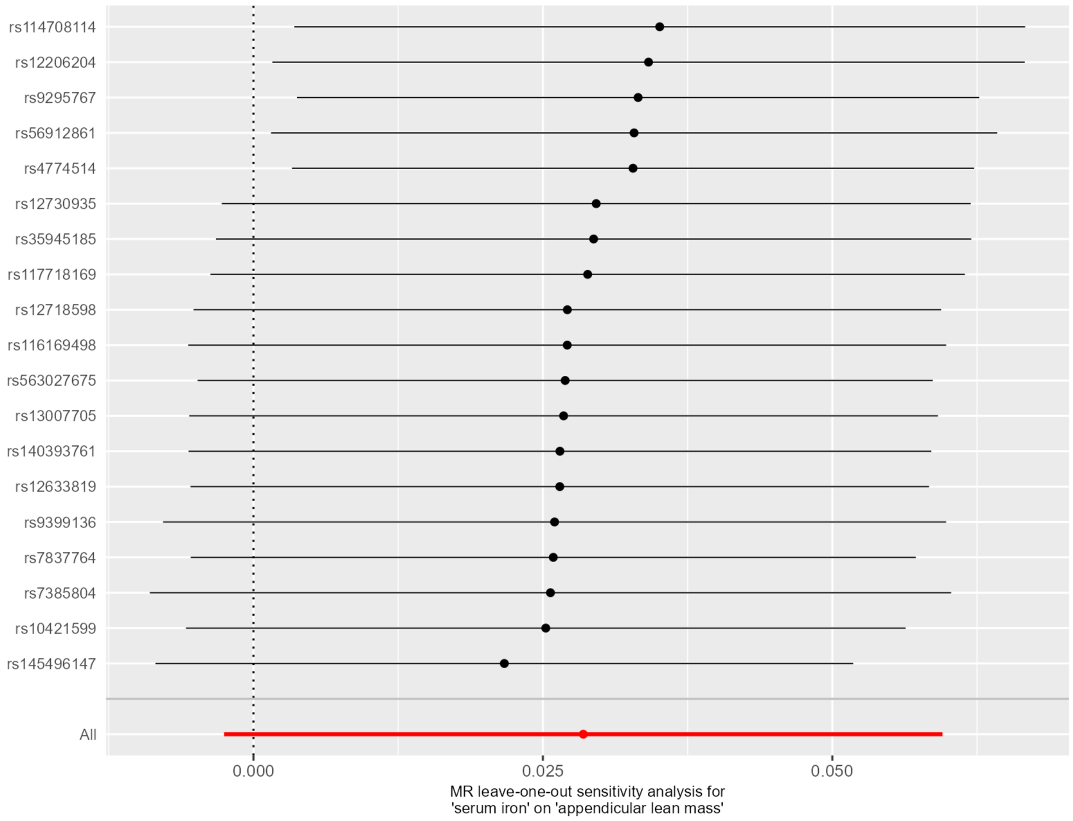

Supplementary Figure 6. Leave-one-out plot for sensitivity analysis of single SNP effect on "serum iron"-to-"appendicular lean mass" UVMR results.

Leave-one-out plot using IVW method by sequentially re-evaluating the causal estimate after discarding one SNP at a time, which helps determine whether the overall effect is driven by the specific genetic variant. The black point denotes the causal effect estimate of serum iron on appendicular lean mass after discarding a certain SNP, and the black line signifies the 95% CI of estimate. The red point symbolizes the causal effect estimate of serum iron on appendicular lean mass, and the red line indicates the 95% CI of the estimate. **Abbreviations:** SNP = number of single-nucleotide polymorphism; UVMR = univariate Mendelian randomization; CI = confidence interval.

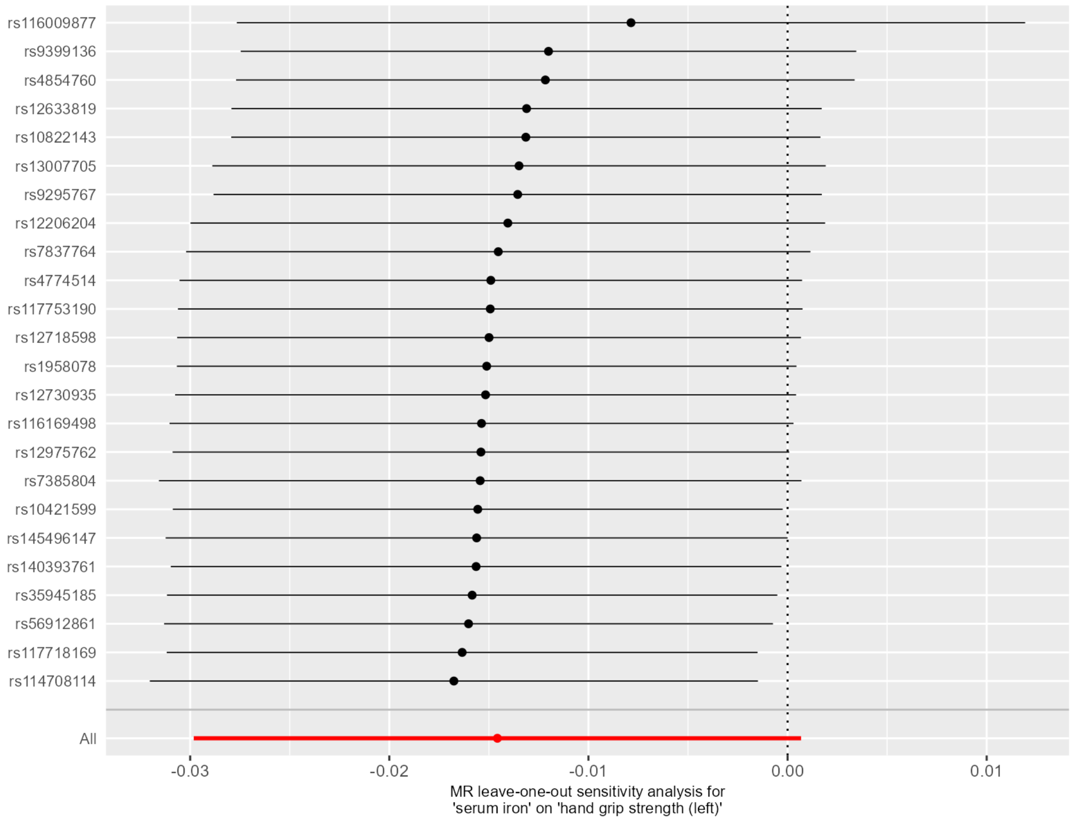

Supplementary Figure 7. Leave-one-out plot for sensitivity analysis of single SNP effect on "serum iron"-to-"hand grip strength (left)" UVMR results.

Leave-one-out plot using IVW method by sequentially re-evaluating the causal estimate after discarding one SNP at a time, which helps determine whether the overall effect is driven by the specific genetic variant. The black point denotes the causal effect estimate of serum iron on hand grip strength (left) after discarding a certain SNP, and the black line signifies the 95% CI of estimate. The red point symbolizes the causal effect estimate of serum iron on hand grip strength (left), and the red line indicates the 95% CI of the estimate. **Abbreviations:** SNP = number of single-nucleotide polymorphism; UVMR = univariate Mendelian randomization; CI = confidence interval.

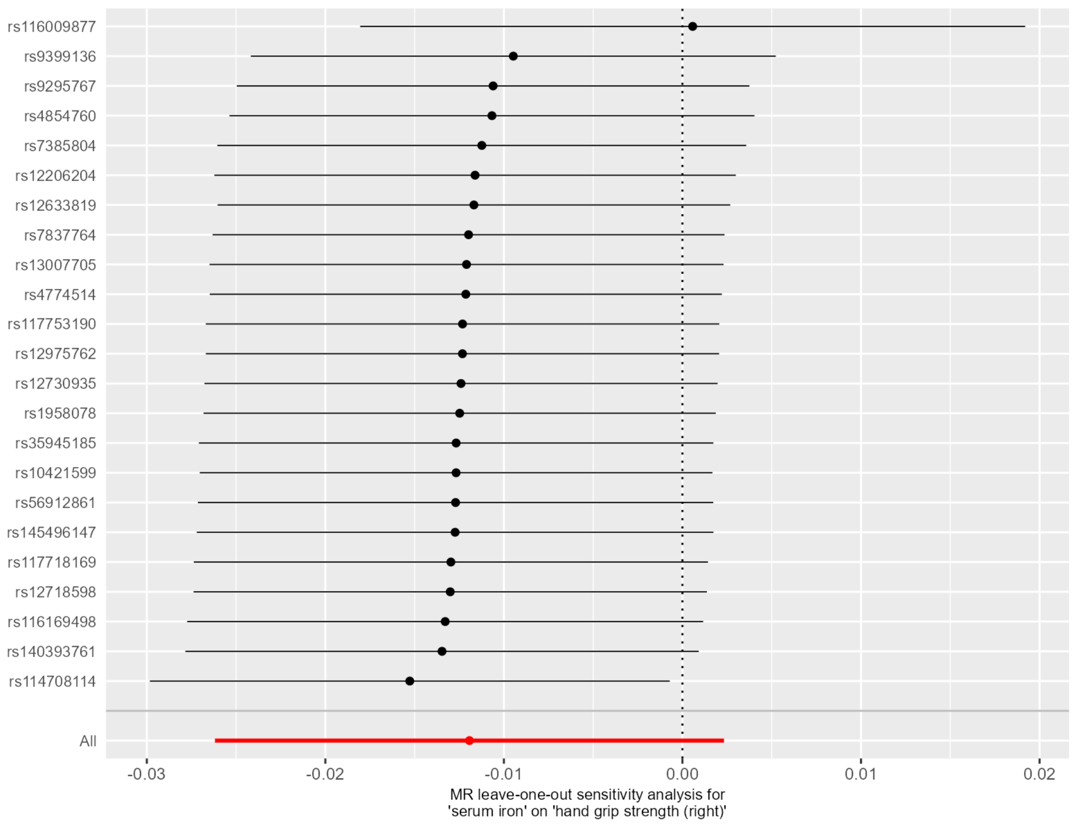

Supplementary Figure 8. Leave-one-out plot for sensitivity analysis of single SNP effect on "serum iron"-to-"hand grip strength (right)" UVMR results.

Leave-one-out plot using IVW method by sequentially re-evaluating the causal estimate after discarding one SNP at a time, which helps determine whether the overall effect is driven by the specific genetic variant. The black point denotes the causal effect estimate of serum iron on hand grip strength (right) after discarding a certain SNP, and the black line signifies the 95% CI of estimate. The red point symbolizes the causal effect estimate of serum iron on hand grip strength (right), and the red line indicates the 95% CI of the estimate. **Abbreviations:** SNP = number of single-nucleotide polymorphism; UVMR = univariate Mendelian randomization; CI = confidence interval.

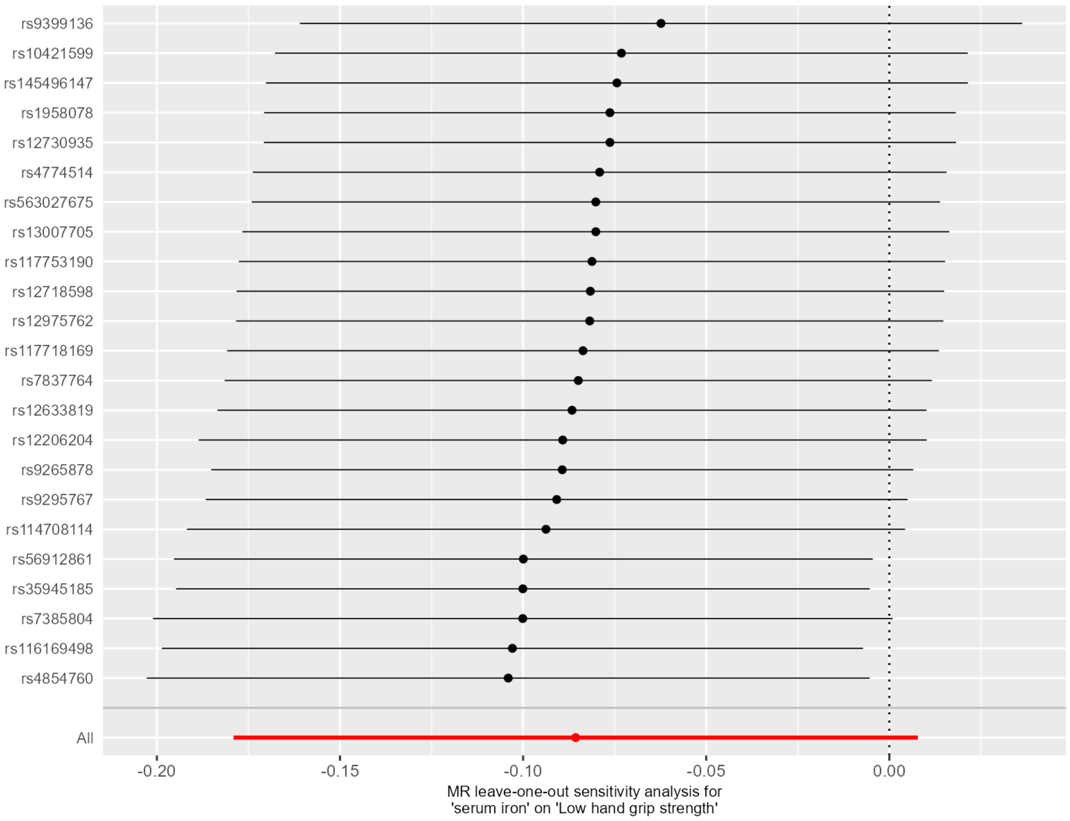

Supplementary Figure 9. Leave-one-out plot for sensitivity analysis of single SNP effect on "serum iron"-to-"low hand grip strength" UVMR results.

Leave-one-out plot using IVW method by sequentially re-evaluating the causal estimate after discarding one SNP at a time, which helps determine whether the overall effect is driven by the specific genetic variant. The black point denotes the causal effect estimate of serum iron on low hand grip strength after discarding a certain SNP, and the black line signifies the 95% CI of estimate. The red point symbolizes the causal effect estimate of serum iron on low hand grip strength, and the red line indicates the 95% CI of the estimate. **Abbreviations:** SNP = number of single-nucleotide polymorphism; UVMR = univariate Mendelian randomization; CI = confidence interval.

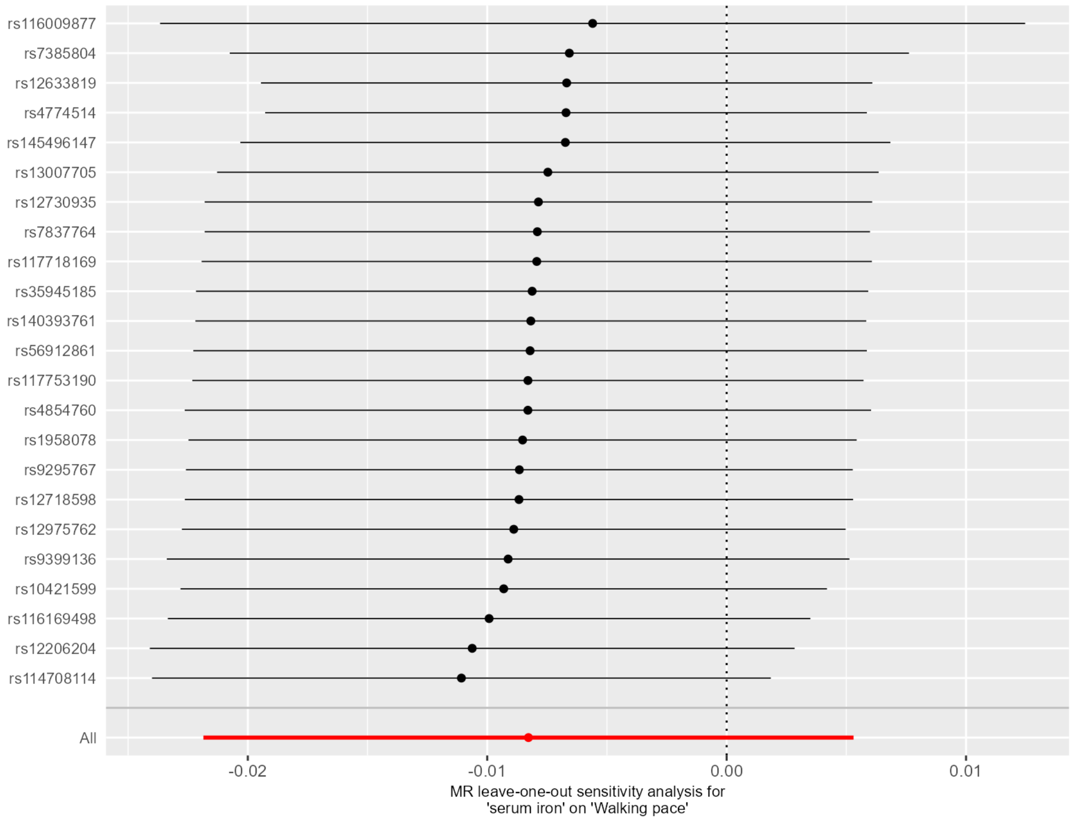

Supplementary Figure 10. Leave-one-out plot for sensitivity analysis of single SNP effect on "serum iron"-to-"walking pace" UVMR results.

Leave-one-out plot using IVW method by sequentially re-evaluating the causal estimate after discarding one SNP at a time, which helps determine whether the overall effect is driven by the specific genetic variant. The black point denotes the causal effect estimate of serum iron on walking pace after discarding a certain SNP, and the black line signifies the 95% CI of estimate. The red point symbolizes the causal effect estimate of serum iron on walking pace, and the red line indicates the 95% CI of the estimate. **Abbreviations:** SNP = number of single-nucleotide polymorphism; UVMR = univariate Mendelian randomization; CI = confidence interval.

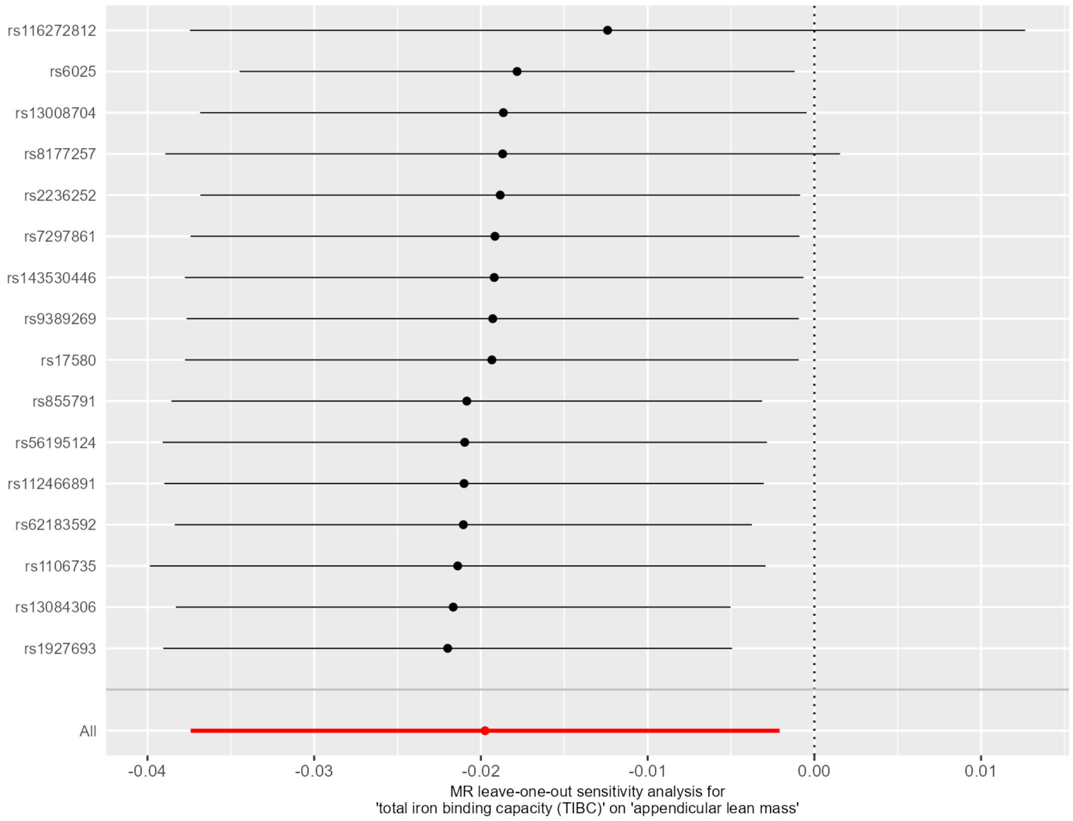

Supplementary Figure 11. Leave-one-out plot for sensitivity analysis of single SNP effect on "total iron binding capacity"-to-"appendicular lean mass" UVMR results.

Leave-one-out plot using IVW method by sequentially re-evaluating the causal estimate after discarding one SNP at a time, which helps determine whether the overall effect is driven by the specific genetic variant. The black point denotes the causal effect estimate of total iron binding capacity on appendicular lean mass after discarding a certain SNP, and the black line signifies the 95% CI of estimate. The red point symbolizes the causal effect estimate of total iron binding capacity on appendicular lean mass, and the red line indicates the 95% CI of the estimate. **Abbreviations:** SNP = number of single-nucleotide polymorphism; UVMR = univariate Mendelian randomization; CI = confidence interval.

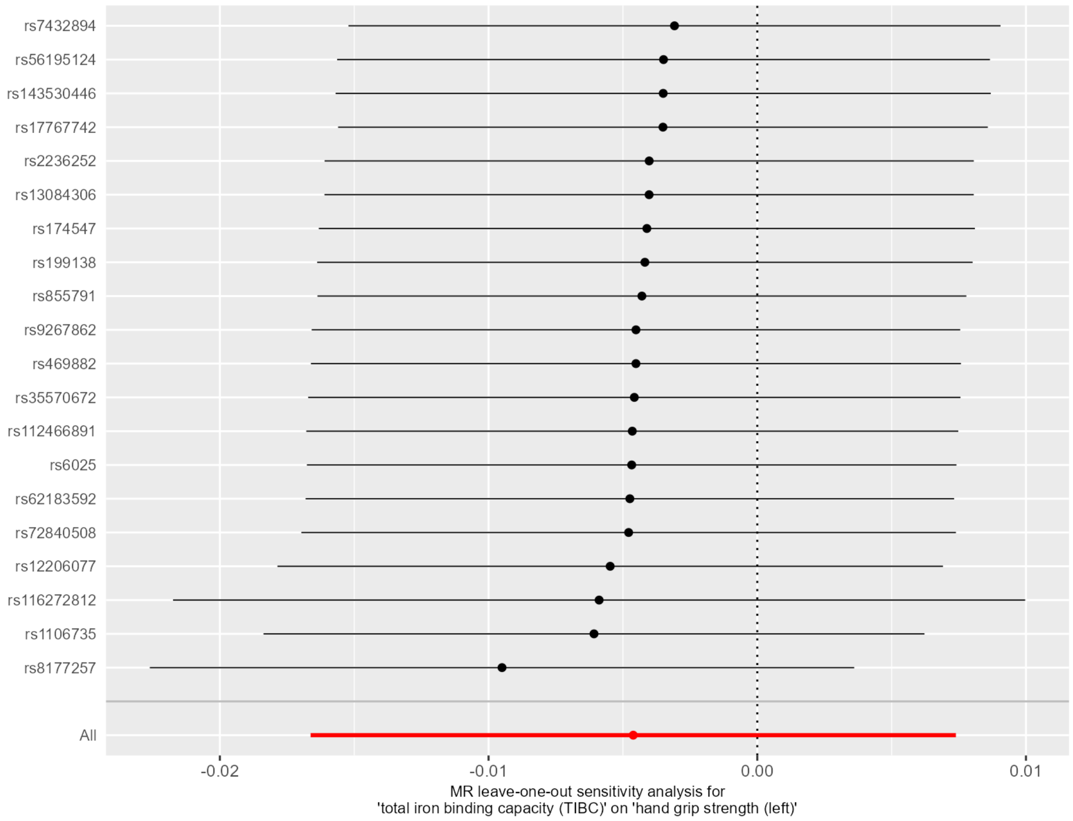

Supplementary Figure 12. Leave-one-out plot for sensitivity analysis of single SNP effect on "total iron binding capacity"-to-"hand grip strength (left)" UVMR results.

Leave-one-out plot using IVW method by sequentially re-evaluating the causal estimate after discarding one SNP at a time, which helps determine whether the overall effect is driven by the specific genetic variant. The black point denotes the causal effect estimate of total iron binding capacity on hand grip strength (left) after discarding a certain SNP, and the black line signifies the 95% CI of estimate. The red point symbolizes the causal effect estimate of total iron binding capacity on hand grip strength (left), and the red line indicates the 95% CI of the estimate. **Abbreviations:** SNP = number of single-nucleotide polymorphism; UVMR = univariate Mendelian randomization; CI = confidence interval.

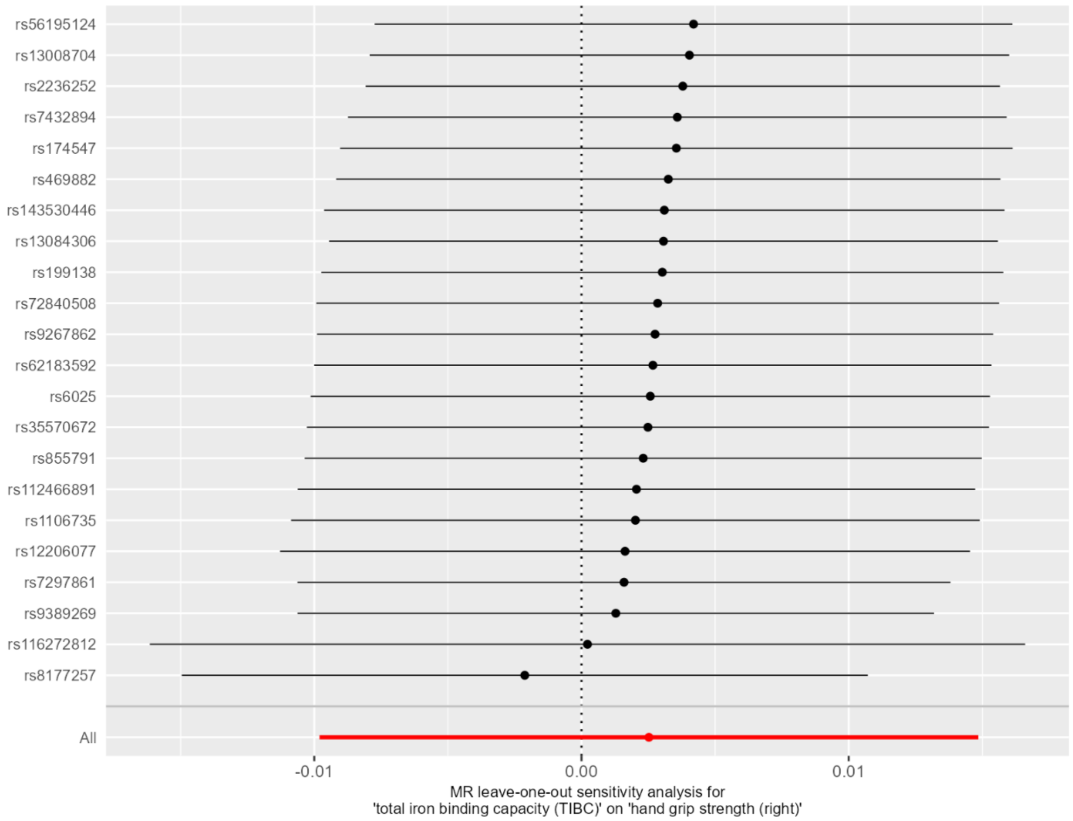

Supplementary Figure 13. Leave-one-out plot for sensitivity analysis of single SNP effect on "total iron binding capacity"-to-"hand grip strength (right)" UVMR results.

Leave-one-out plot using IVW method by sequentially re-evaluating the causal estimate after discarding one SNP at a time, which helps determine whether the overall effect is driven by the specific genetic variant. The black point denotes the causal effect estimate of total iron binding capacity on hand grip strength (right) after discarding a certain SNP, and the black line signifies the 95% CI of estimate. The red point symbolizes the causal effect estimate of total iron binding capacity on hand grip strength (right), and the red line indicates the 95% CI of the estimate. **Abbreviations:** SNP = number of single-nucleotide polymorphism; UVMR = univariate Mendelian randomization; CI = confidence interval.

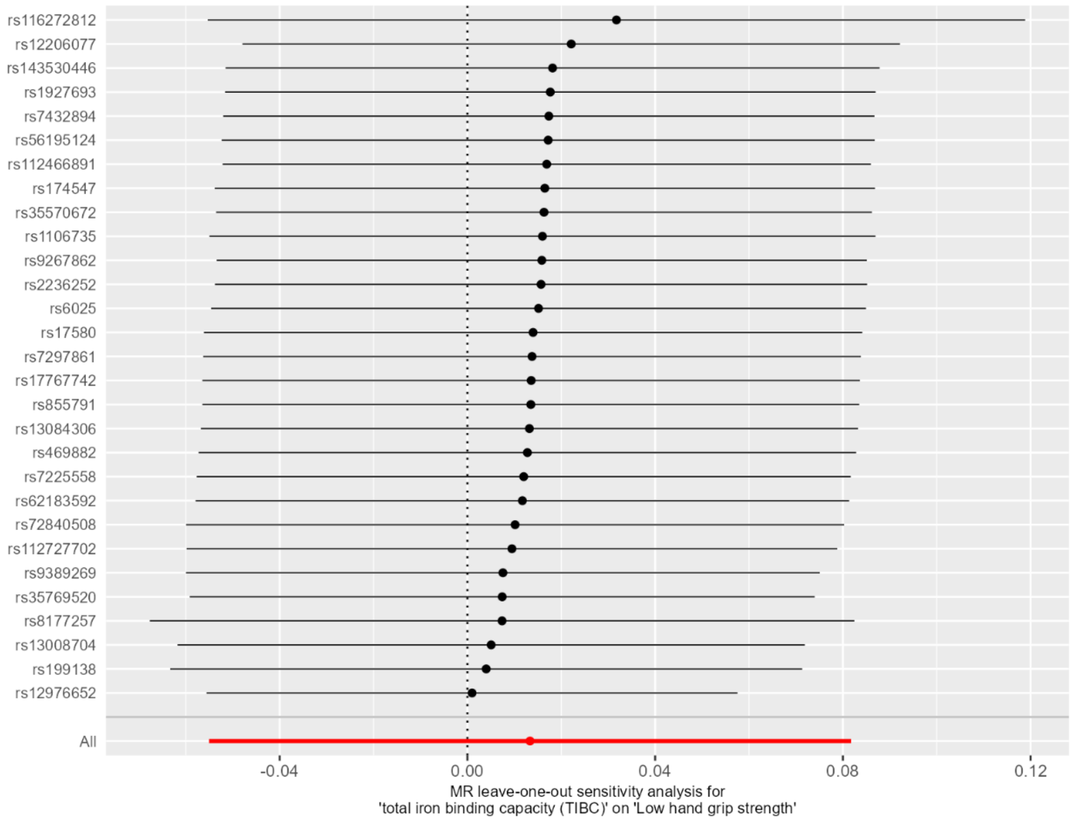

Supplementary Figure 14. Leave-one-out plot for sensitivity analysis of single SNP effect on "total iron binding capacity"-to-"low hand grip strength" UVMR results.

Leave-one-out plot using IVW method by sequentially re-evaluating the causal estimate after discarding one SNP at a time, which helps determine whether the overall effect is driven by the specific genetic variant. The black point denotes the causal effect estimate of total iron binding capacity on low hand grip strength after discarding a certain SNP, and the black line signifies the 95% CI of estimate. The red point symbolizes the causal effect estimate of total iron binding capacity on low hand grip strength, and the red line indicates the 95% CI of the estimate. **Abbreviations:** SNP = number of single-nucleotide polymorphism; UVMR = univariate Mendelian randomization; CI = confidence interval.

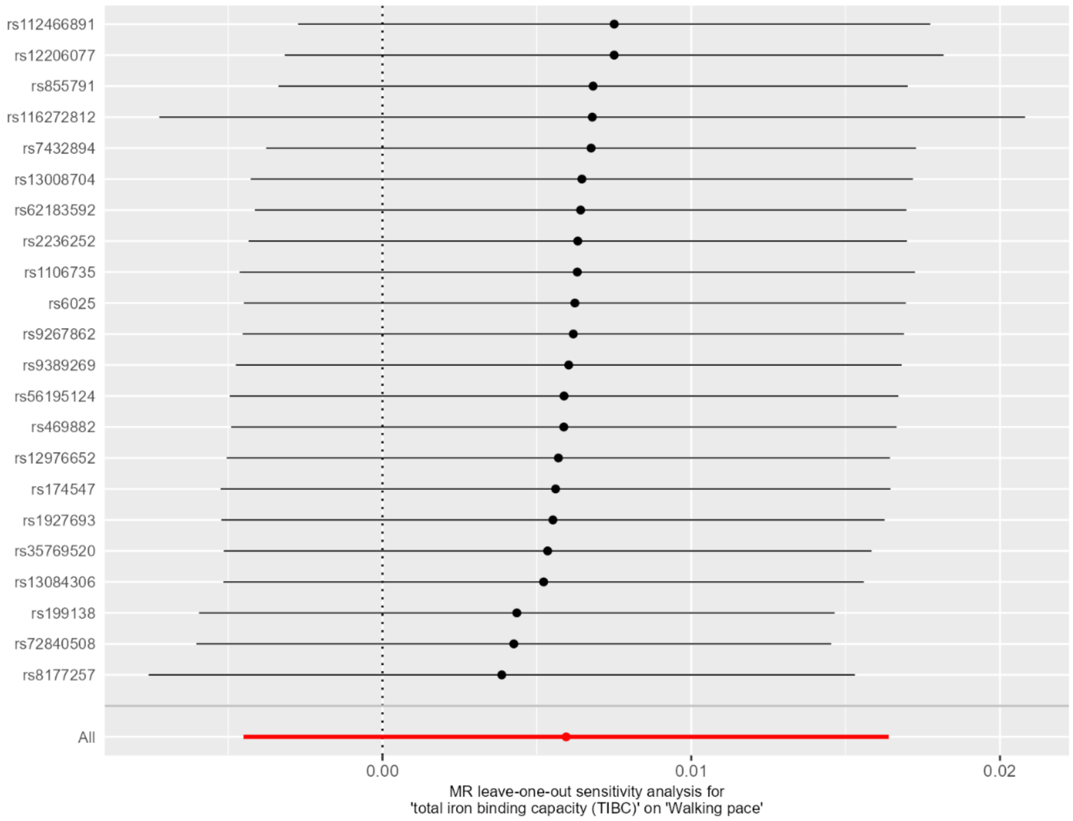

Supplementary Figure 15. Leave-one-out plot for sensitivity analysis of single SNP effect on "total iron binding capacity"-to-"walking pace" UVMR results.

Leave-one-out plot using IVW method by sequentially re-evaluating the causal estimate after discarding one SNP at a time, which helps determine whether the overall effect is driven by the specific genetic variant. The black point denotes the causal effect estimate of total iron binding capacity on walking pace after discarding a certain SNP, and the black line signifies the 95% CI of estimate. The red point symbolizes the causal effect estimate of total iron binding capacity on walking pace, and the red line indicates the 95% CI of the estimate. **Abbreviations:** SNP = number of single-nucleotide polymorphism; UVMR = univariate Mendelian randomization; CI = confidence interval.

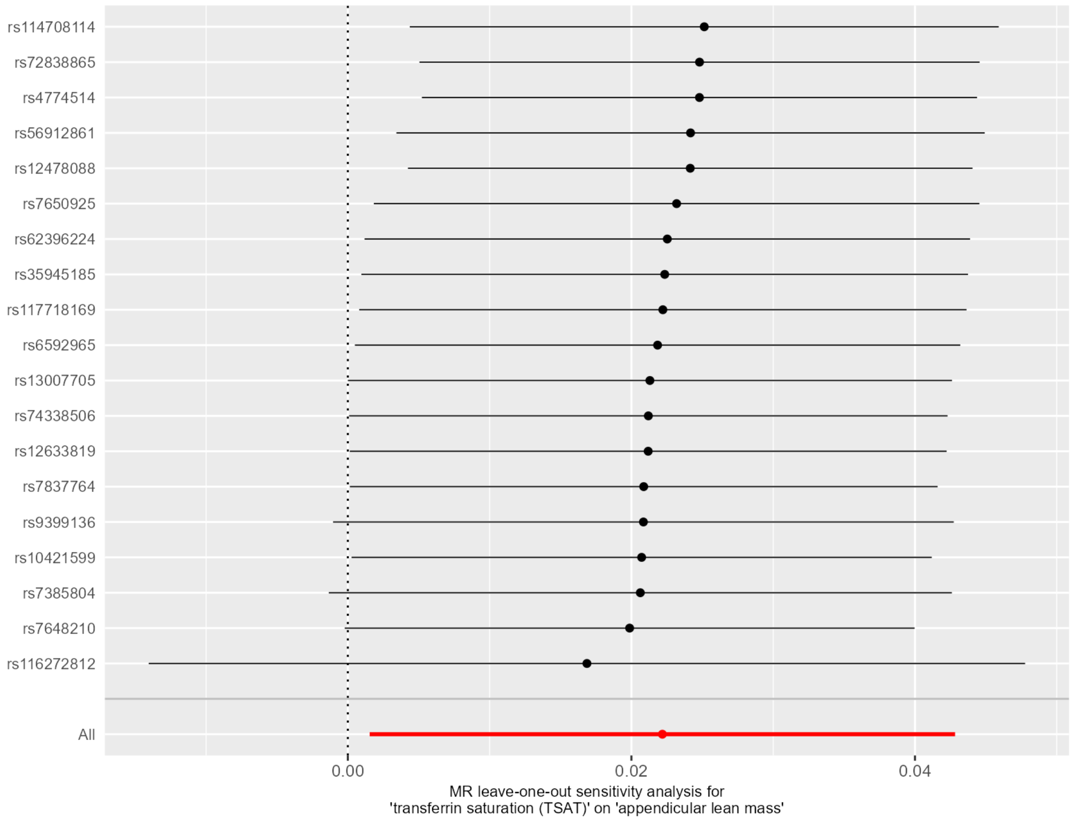

Supplementary Figure 16. Leave-one-out plot for sensitivity analysis of single SNP effect on "transferrin saturation"-to-"appendicular lean mass" UVMR results.

Leave-one-out plot using IVW method by sequentially re-evaluating the causal estimate after discarding one SNP at a time, which helps determine whether the overall effect is driven by the specific genetic variant. The black point denotes the causal effect estimate of transferrin saturation on appendicular lean mass after discarding a certain SNP, and the black line signifies the 95% CI of estimate. The red point symbolizes the causal effect estimate of transferrin saturation on appendicular lean mass, and the red line indicates the 95% CI of the estimate. **Abbreviations:** SNP = number of single-nucleotide polymorphism; UVMR = univariate Mendelian randomization; CI = confidence interval.

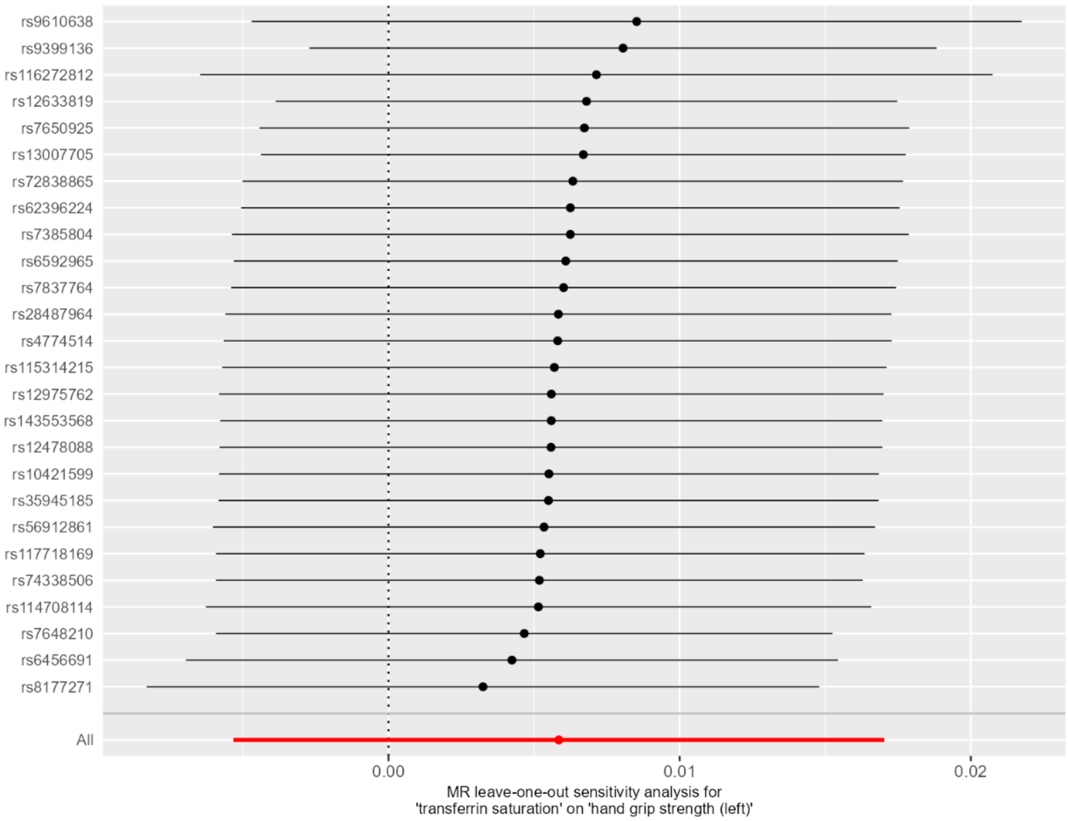

Supplementary Figure 17. Leave-one-out plot for sensitivity analysis of single SNP effect on "transferrin saturation"-to-"hand grip strength (left)" UVMR results.

Leave-one-out plot using IVW method by sequentially re-evaluating the causal estimate after discarding one SNP at a time, which helps determine whether the overall effect is driven by the specific genetic variant. The black point denotes the causal effect estimate of transferrin saturation on hand grip strength (left) after discarding a certain SNP, and the black line signifies the 95% CI of estimate. The red point symbolizes the causal effect estimate of transferrin saturation on hand grip strength (left), and the red line indicates the 95% CI of the estimate. **Abbreviations:** SNP = number of single-nucleotide polymorphism; UVMR = univariate Mendelian randomization; CI = confidence interval.

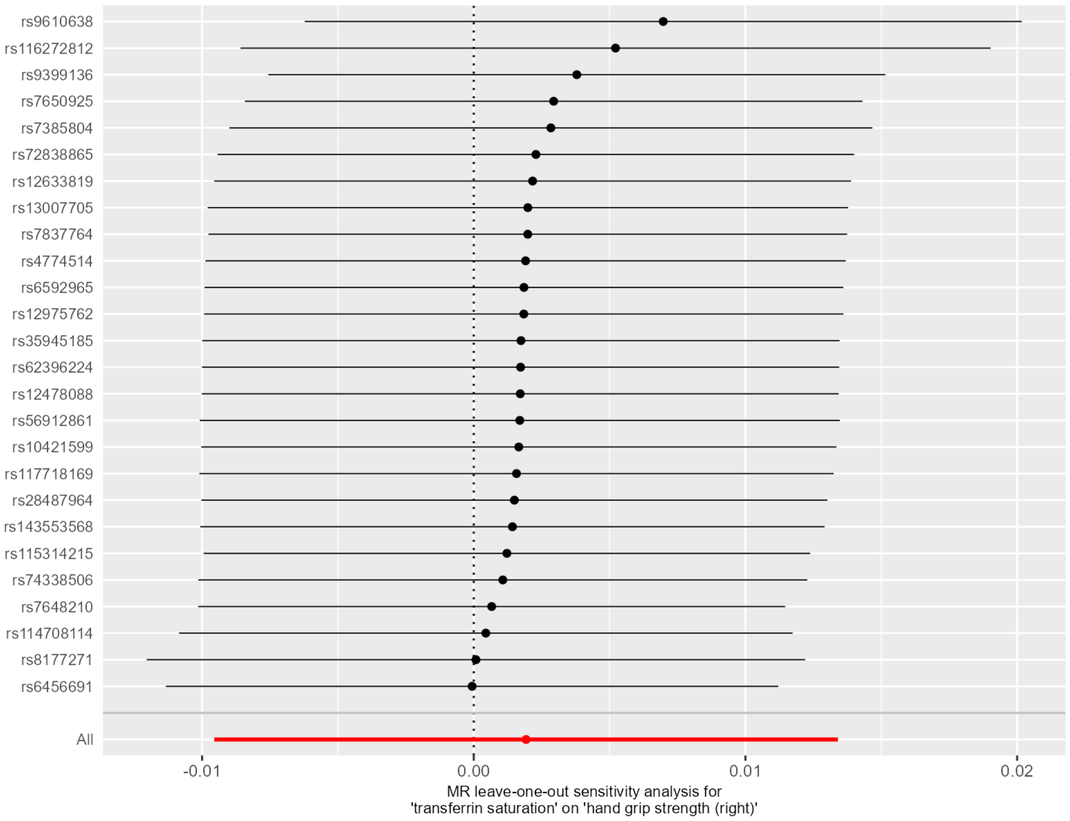

Supplementary Figure 18. Leave-one-out plot for sensitivity analysis of single SNP effect on "transferrin saturation"-to-"hand grip strength (right)" UVMR results.

Leave-one-out plot using IVW method by sequentially re-evaluating the causal estimate after discarding one SNP at a time, which helps determine whether the overall effect is driven by the specific genetic variant. The black point denotes the causal effect estimate of transferrin saturation on hand grip strength (right) after discarding a certain SNP, and the black line signifies the 95% CI of estimate. The red point symbolizes the causal effect estimate of transferrin saturation on hand grip strength (right), and the red line indicates the 95% CI of the estimate. **Abbreviations:** SNP = number of single-nucleotide polymorphism; UVMR = univariate Mendelian randomization; CI = confidence interval.

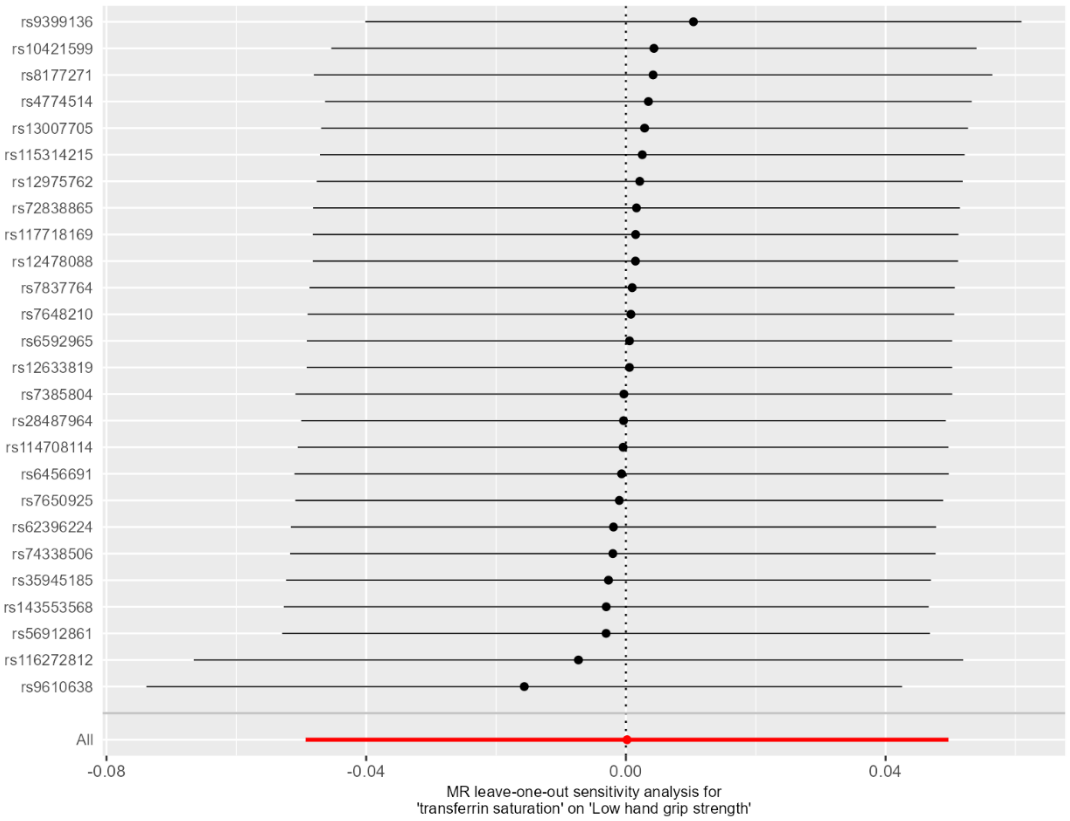

Supplementary Figure 19. Leave-one-out plot for sensitivity analysis of single SNP effect on "transferrin saturation"-to-"low hand grip strength" UVMR results.

Leave-one-out plot using IVW method by sequentially re-evaluating the causal estimate after discarding one SNP at a time, which helps determine whether the overall effect is driven by the specific genetic variant. The black point denotes the causal effect estimate of transferrin saturation on low hand grip strength after discarding a certain SNP, and the black line signifies the 95% CI of estimate. The red point symbolizes the causal effect estimate of transferrin saturation on low hand grip strength, and the red line indicates the 95% CI of the estimate. **Abbreviations:** SNP = number of single-nucleotide polymorphism; UVMR = univariate Mendelian randomization; CI = confidence interval.

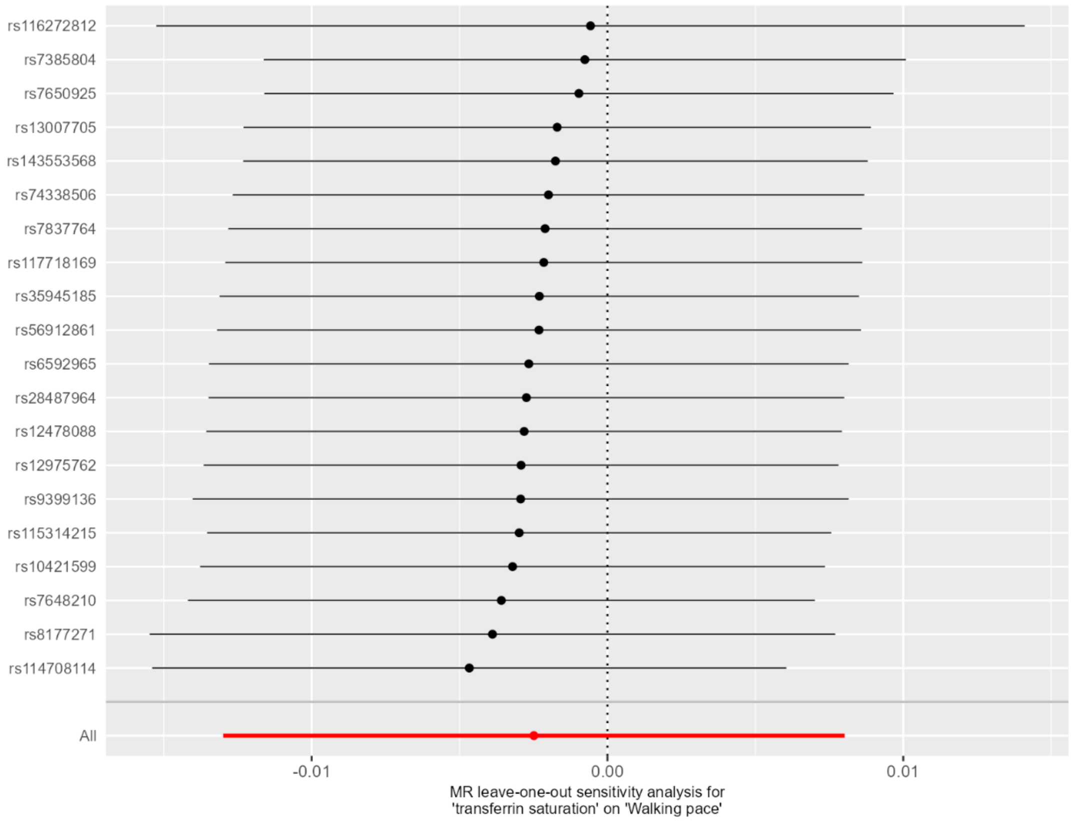

Supplementary Figure 20. Leave-one-out plot for sensitivity analysis of single SNP effect on "transferrin saturation"-to-"walking pace" UVMR results.

Leave-one-out plot using IVW method by sequentially re-evaluating the causal estimate after discarding one SNP at a time, which helps determine whether the overall effect is driven by the specific genetic variant. The black point denotes the causal effect estimate of transferrin saturation on walking pace after discarding a certain SNP, and the black line signifies the 95% CI of estimate. The red point symbolizes the causal effect estimate of transferrin saturation on walking pace, and the red line indicates the 95% CI of the estimate. **Abbreviations:** SNP = number of single-nucleotide polymorphism; UVMR = univariate Mendelian randomization; CI = confidence interval.

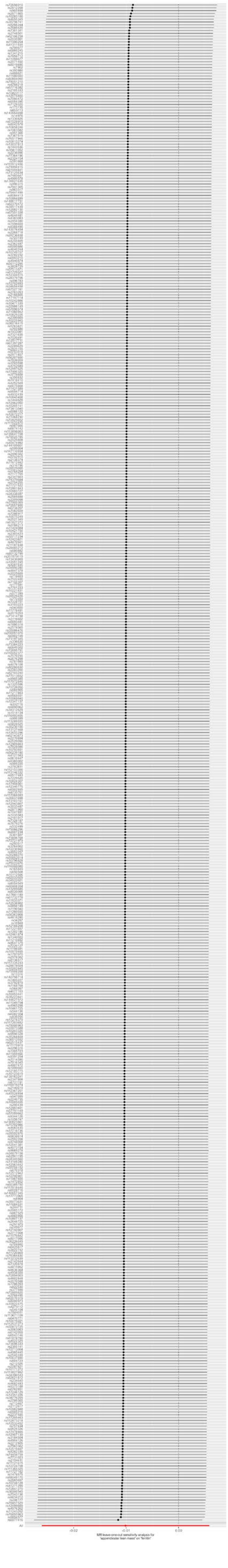
All
-0.02
-0.01
0.00
MR leave-one-out sensitivity analysis for
'appendicular lean mass' on 'ferritin'

Supplementary Figure 21. Leave-one-out plot for sensitivity analysis of single SNP effect on "appendicular lean mass"-to-"ferritin" UVMR results.

Leave-one-out plot using IVW method by sequentially re-evaluating the causal estimate after discarding one SNP at a time, which helps determine whether the overall effect is driven by the specific genetic variant. The black point denotes the causal effect estimate of appendicular lean mass on ferritin after discarding a certain SNP, and the black line signifies the 95% CI of estimate. The red point symbolizes the causal effect estimate of appendicular lean mass on ferritin, and the red line indicates the 95% CI of the estimate. **Abbreviations:** SNP = number of single-nucleotide polymorphism; UVMR = univariate Mendelian randomization; CI = confidence interval.

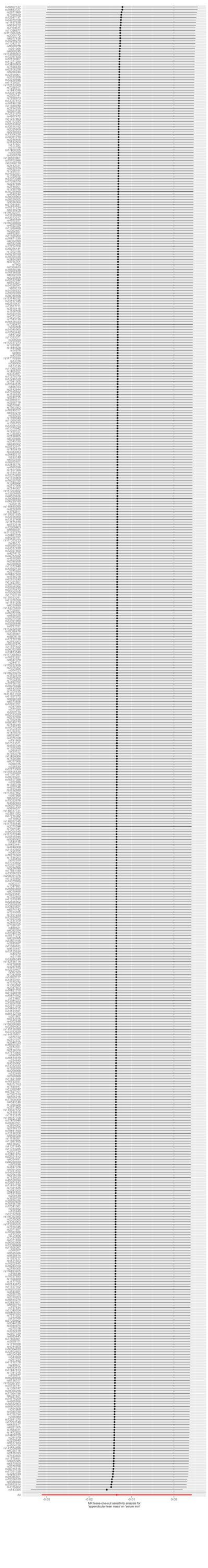
All
-0.03
-0.02
-0.01
0.00
MR leave-one-out sensitivity analysis for
'appendicular lean mass' on 'serum iron'

Supplementary Figure 22. Leave-one-out plot for sensitivity analysis of single SNP effect on "appendicular lean mass"-to-"serum iron" UVMR results.

Leave-one-out plot using IVW method by sequentially re-evaluating the causal estimate after discarding one SNP at a time, which helps determine whether the overall effect is driven by the specific genetic variant. The black point denotes the causal effect estimate of appendicular lean mass on serum iron after discarding a certain SNP, and the black line signifies the 95% CI of estimate. The red point symbolizes the causal effect estimate of appendicular lean mass on serum iron, and the red line indicates the 95% CI of the estimate. **Abbreviations:** SNP = number of single-nucleotide polymorphism; UVMR = univariate Mendelian randomization; CI = confidence interval.

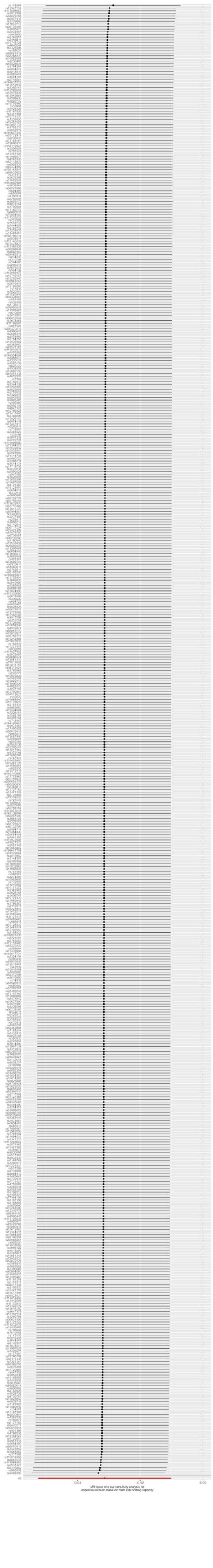
All
-0.04
-0.02
0.00
MR leave-one-out sensitivity analysis for
'appendicular lean mass' on 'total iron binding capacity'

Supplementary Figure 23. Leave-one-out plot for sensitivity analysis of single SNP effect on "appendicular lean mass"-to-"total iron binding capacity" UVMR results.

Leave-one-out plot using IVW method by sequentially re-evaluating the causal estimate after discarding one SNP at a time, which helps determine whether the overall effect is driven by the specific genetic variant. The black point denotes the causal effect estimate of appendicular lean mass on total iron binding capacity after discarding a certain SNP, and the black line signifies the 95% CI of estimate. The red point symbolizes the causal effect estimate of appendicular lean mass on total iron binding capacity, and the red line indicates the 95% CI of the estimate. **Abbreviations:** SNP = number of single-nucleotide polymorphism; UVMR = univariate Mendelian randomization; CI = confidence interval.

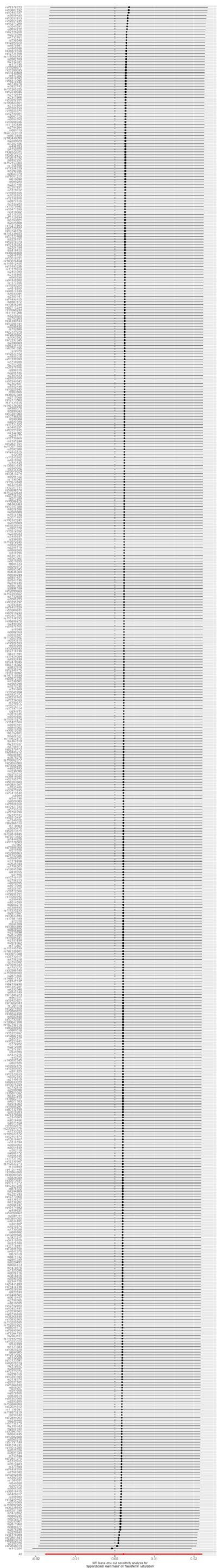
All
-0.02
-0.01
0.00
0.01
0.02
MR leave-one-out sensitivity analysis for
'appendicular lean mass' on 'transferrin saturation'

Supplementary Figure 24. Leave-one-out plot for sensitivity analysis of single SNP effect on "appendicular lean mass"-to-"transferrin saturation" UVMR results.

Leave-one-out plot using IVW method by sequentially re-evaluating the causal estimate after discarding one SNP at a time, which helps determine whether the overall effect is driven by the specific genetic variant. The black point denotes the causal effect estimate of appendicular lean mass on transferrin saturation after discarding a certain SNP, and the black line signifies the 95% CI of estimate. The red point symbolizes the causal effect estimate of appendicular lean mass on transferrin saturation, and the red line indicates the 95% CI of the estimate. **Abbreviations:** SNP = number of single-nucleotide polymorphism; UVMR = univariate Mendelian randomization; CI = confidence interval.

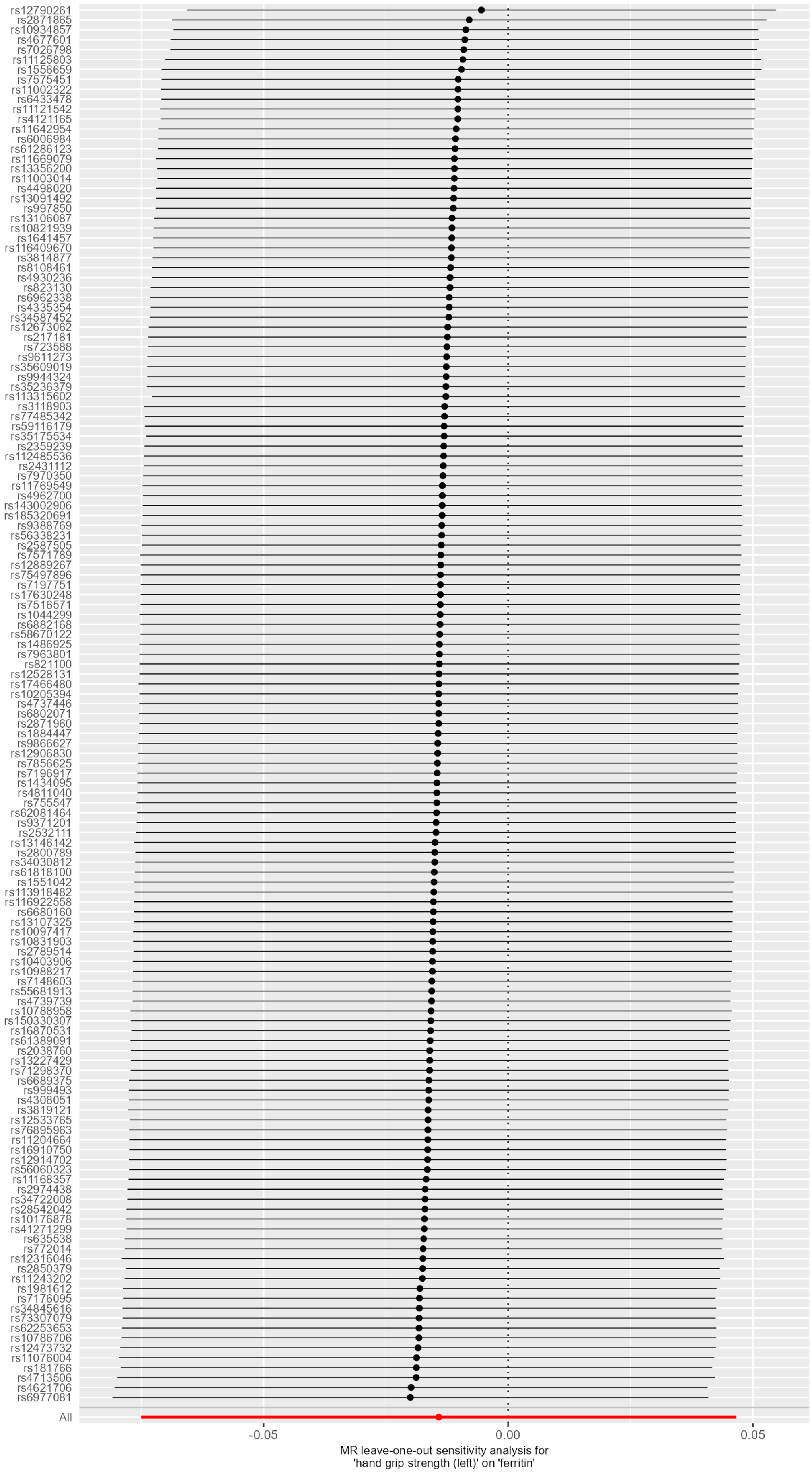
rs12790261
rs2871865
rs10934857
rs4677601
rs7026798
rs11125803
rs1556659
rs7575451
rs11002322
rs6433478
rs11121542
rs4121165
rs11642954
rs6006984
rs61286123
rs11669079
rs13356200
rs11003014
rs4498020
rs13091492
rs997850
rs13106087
rs10821939
rs1641457
rs116409670
rs3814877
rs8108461
rs4930236
rs823130
rs6962338
rs4335354
rs34587452
rs12673062
rs217181
rs723588
rs9611273
rs35609019
rs9944324
rs35236379
rs113315602
rs3118903
rs77485342
rs59116179
rs35175534
rs2359239
rs112485536
rs2431112
rs7970350
rs11769549
rs4962700
rs143002906
rs185320691
rs9388769
rs56338231
rs2587505
rs7571789
rs12889267
rs75497896
rs7197751
rs17630248
rs7516571
rs1044299
rs6882168
rs58670122
rs1486925
rs7963801
rs821100
rs12528131
rs17466480
rs10205394
rs4737446
rs6802071
rs2871960
rs1884447
rs9866627
rs12906830
rs7856625
rs7196917
rs1434095
rs4811040
rs755547
rs62081464
rs9371201
rs2532111
rs13146142
rs2800789
rs34030812
rs61818100
rs1551042
rs113918482
rs116922558
rs6680160
rs13107325
rs10097417
rs10831903
rs2789514
rs10403906
rs10988217
rs7148603
rs55681913
rs4739739
rs10788958
rs150330307
rs16870531
rs61389091
rs2038760
rs13227429
rs71298370
rs6689375
rs999493
rs4308051
rs3819121
rs12533765
rs76895963
rs11204664
rs16910750
rs12914702
rs56060323
rs11168357
rs2974438
rs34722008
rs28542042
rs10176878
rs41271299
rs635538
rs772014
rs12316046
rs2850379
rs11243202
rs1981612
rs7176095
rs34845616
rs73307079
rs62253653
rs10786706
rs12473732
rs11076004
rs181766
rs4713506
rs4621706
rs6977081
All
-0.05
0.00
0.05
MR leave-one-out sensitivity analysis for
'hand grip strength (left)' on 'ferritin'

Supplementary Figure 25. Leave-one-out plot for sensitivity analysis of single SNP effect on "hand grip strength (left)"-to-"ferritin" UVMR results.

Leave-one-out plot using IVW method by sequentially re-evaluating the causal estimate after discarding one SNP at a time, which helps determine whether the overall effect is driven by the specific genetic variant. The black point denotes the causal effect estimate of hand grip strength (left) on ferritin after discarding a certain SNP, and the black line signifies the 95% CI of estimate. The red point symbolizes the causal effect estimate of hand grip strength (left) on ferritin, and the red line indicates the 95% CI of the estimate. **Abbreviations:** SNP = number of single-nucleotide polymorphism; UVMR = univariate Mendelian randomization; CI = confidence interval.

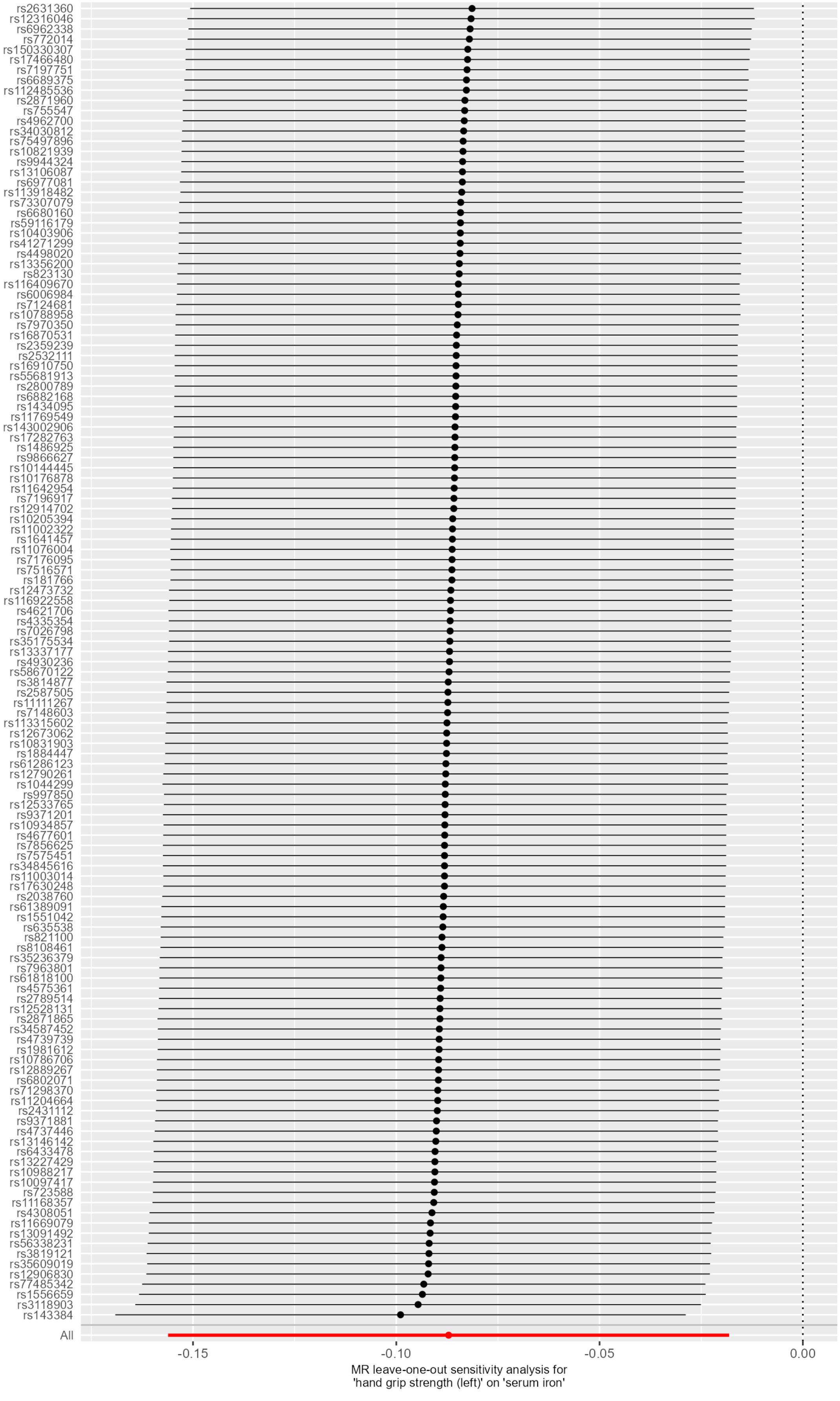
rs2631360
rs12316046
rs6962338
rs772014
rs150330307
rs17466480
rs7197751
rs6689375
rs112485536
rs2871960
rs755547
rs4962700
rs34030812
rs75497896
rs10821939
rs9944324
rs13106087
rs6977081
rs113918482
rs73307079
rs6680160
rs59116179
rs10403906
rs41271299
rs4498020
rs13356200
rs823130
rs116409670
rs6006984
rs7124681
rs10788958
rs7970350
rs16870531
rs2359239
rs2532111
rs16910750
rs55681913
rs2800789
rs6882168
rs1434095
rs11769549
rs143002906
rs17282763
rs1486925
rs9866627
rs10144445
rs10176878
rs11642954
rs7196917
rs12914702
rs10205394
rs11002322
rs1641457
rs11076004
rs7176095
rs7516571
rs181766
rs12473732
rs116922558
rs4621706
rs4335354
rs7026798
rs35175534
rs13337177
rs4930236
rs58670122
rs3814877
rs2587505
rs11111267
rs7148603
rs113315602
rs12673062
rs10831903
rs1884447
rs61286123
rs12790261
rs1044299
rs997850
rs12533765
rs9371201
rs10934857
rs4677601
rs7856625
rs7575451
rs34845616
rs11003014
rs17630248
rs2038760
rs61389091
rs1551042
rs635538
rs821100
rs8108461
rs35236379
rs7963801
rs61818100
rs4575361
rs2789514
rs12528131
rs2871865
rs34587452
rs4739739
rs1981612
rs10786706
rs12889267
rs6802071
rs71298370
rs11204664
rs2431112
rs9371881
rs4737446
rs13146142
rs6433478
rs13227429
rs10988217
rs10097417
rs723588
rs11168357
rs4308051
rs11669079
rs13091492
rs56338231
rs3819121
rs35609019
rs12906830
rs77485342
rs1556659
rs3118903
rs143384
All
-0.15
-0.10
-0.05
0.00
MR leave-one-out sensitivity analysis for
'hand grip strength (left)' on 'serum iron'

Supplementary Figure 26. Leave-one-out plot for sensitivity analysis of single SNP effect on "hand grip strength (left)"-to-"serum iron" UVMR results.

Leave-one-out plot using IVW method by sequentially re-evaluating the causal estimate after discarding one SNP at a time, which helps determine whether the overall effect is driven by the specific genetic variant. The black point denotes the causal effect estimate of hand grip strength (left) on serum iron after discarding a certain SNP, and the black line signifies the 95% CI of estimate. The red point symbolizes the causal effect estimate of hand grip strength (left) on serum iron, and the red line indicates the 95% CI of the estimate. **Abbreviations:** SNP = number of single-nucleotide polymorphism; UVMR = univariate Mendelian randomization; CI = confidence interval.

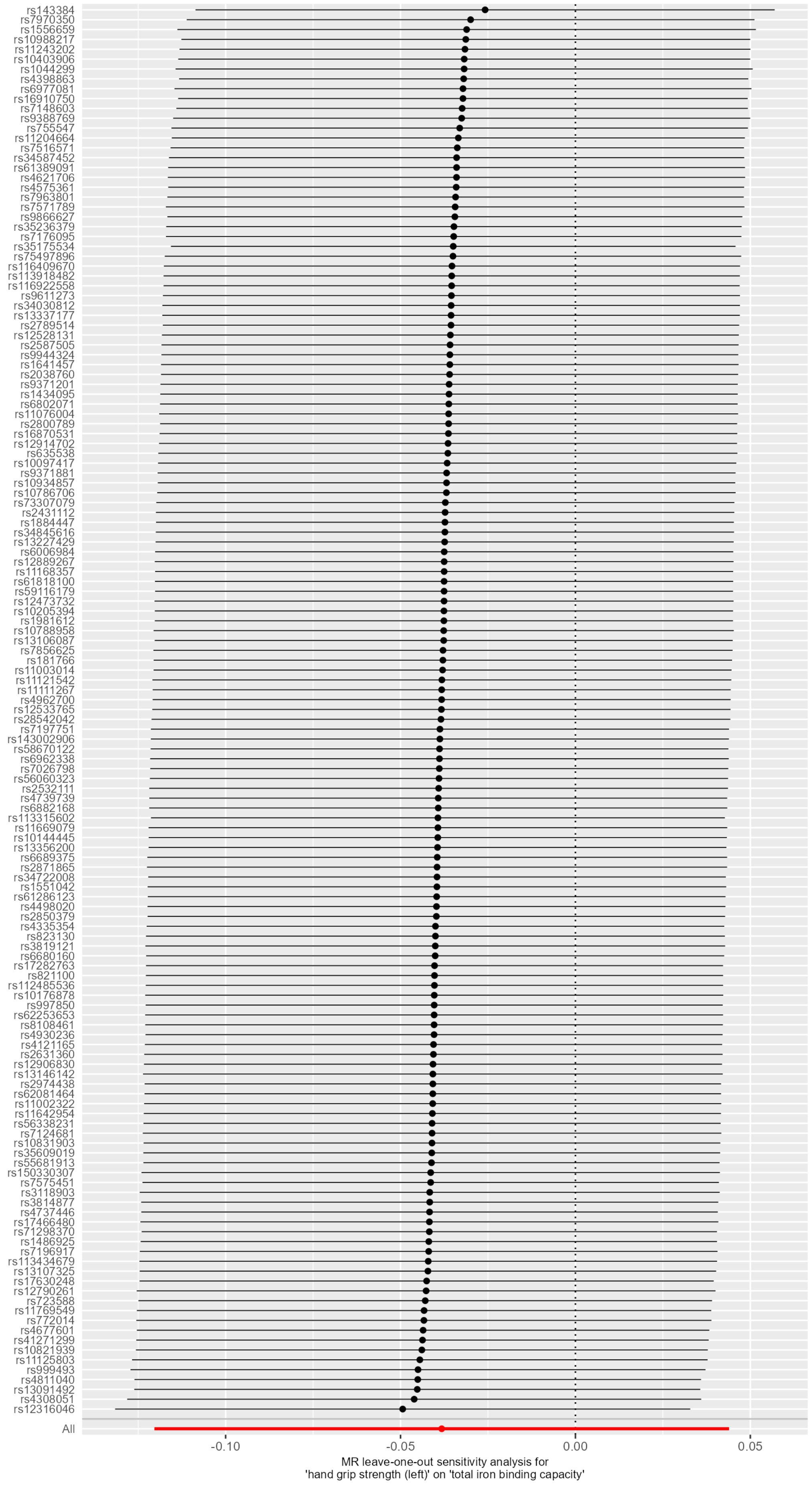
rs143384
rs7970350
rs1556659
rs10988217
rs11243202
rs10403906
rs1044299
rs4398863
rs6977081
rs16910750
rs7148603
rs9388769
rs755547
rs11204664
rs7516571
rs34587452
rs61389091
rs4621706
rs4575361
rs7963801
rs7571789
rs9866627
rs35236379
rs7176095
rs35175534
rs75497896
rs116409670
rs113918482
rs116922558
rs9611273
rs34030812
rs13337177
rs2789514
rs12528131
rs2587505
rs9944324
rs1641457
rs2038760
rs9371201
rs1434095
rs6802071
rs11076004
rs2800789
rs16870531
rs12914702
rs635538
rs10097417
rs9371881
rs10934857
rs10786706
rs73307079
rs2431112
rs1884447
rs34845616
rs13227429
rs6006984
rs12889267
rs11168357
rs61818100
rs59116179
rs12473732
rs10205394
rs1981612
rs10788958
rs13106087
rs7856625
rs181766
rs11003014
rs11121542
rs11111267
rs4962700
rs12533765
rs28542042
rs7197751
rs143002906
rs58670122
rs6962338
rs7026798
rs56060323
rs2532111
rs4739739
rs6882168
rs113315602
rs11669079
rs10144445
rs13356200
rs6689375
rs2871865
rs34722008
rs1551042
rs61286123
rs4498020
rs2850379
rs4335354
rs823130
rs3819121
rs6680160
rs17282763
rs821100
rs112485536
rs10176878
rs997850
rs62253653
rs8108461
rs4930236
rs4121165
rs2631360
rs12906830
rs13146142
rs2974438
rs62081464
rs11002322
rs11642954
rs56338231
rs7124681
rs10831903
rs35609019
rs55681913
rs150330307
rs7575451
rs3118903
rs3814877
rs4737446
rs17466480
rs71298370
rs1486925
rs7196917
rs113434679
rs13107325
rs17630248
rs12790261
rs723588
rs11769549
rs772014
rs4677601
rs41271299
rs10821939
rs11125803
rs999493
rs4811040
rs13091492
rs4308051
rs12316046
All
-0.10
-0.05
0.00
0.05
MR leave-one-out sensitivity analysis for
'hand grip strength (left)' on 'total iron binding capacity'

Supplementary Figure 27. Leave-one-out plot for sensitivity analysis of single SNP effect on "hand grip strength (left)"-to-"total iron binding capacity" UVMR results.

Leave-one-out plot using IVW method by sequentially re-evaluating the causal estimate after discarding one SNP at a time, which helps determine whether the overall effect is driven by the specific genetic variant. The black point denotes the causal effect estimate of hand grip strength (left) on total iron binding capacity after discarding a certain SNP, and the black line signifies the 95% CI of estimate. The red point symbolizes the causal effect estimate of hand grip strength (left) on total iron binding capacity, and the red line indicates the 95% CI of the estimate. **Abbreviations:** SNP = number of single-nucleotide polymorphism; UVMR = univariate Mendelian randomization; CI = confidence interval.

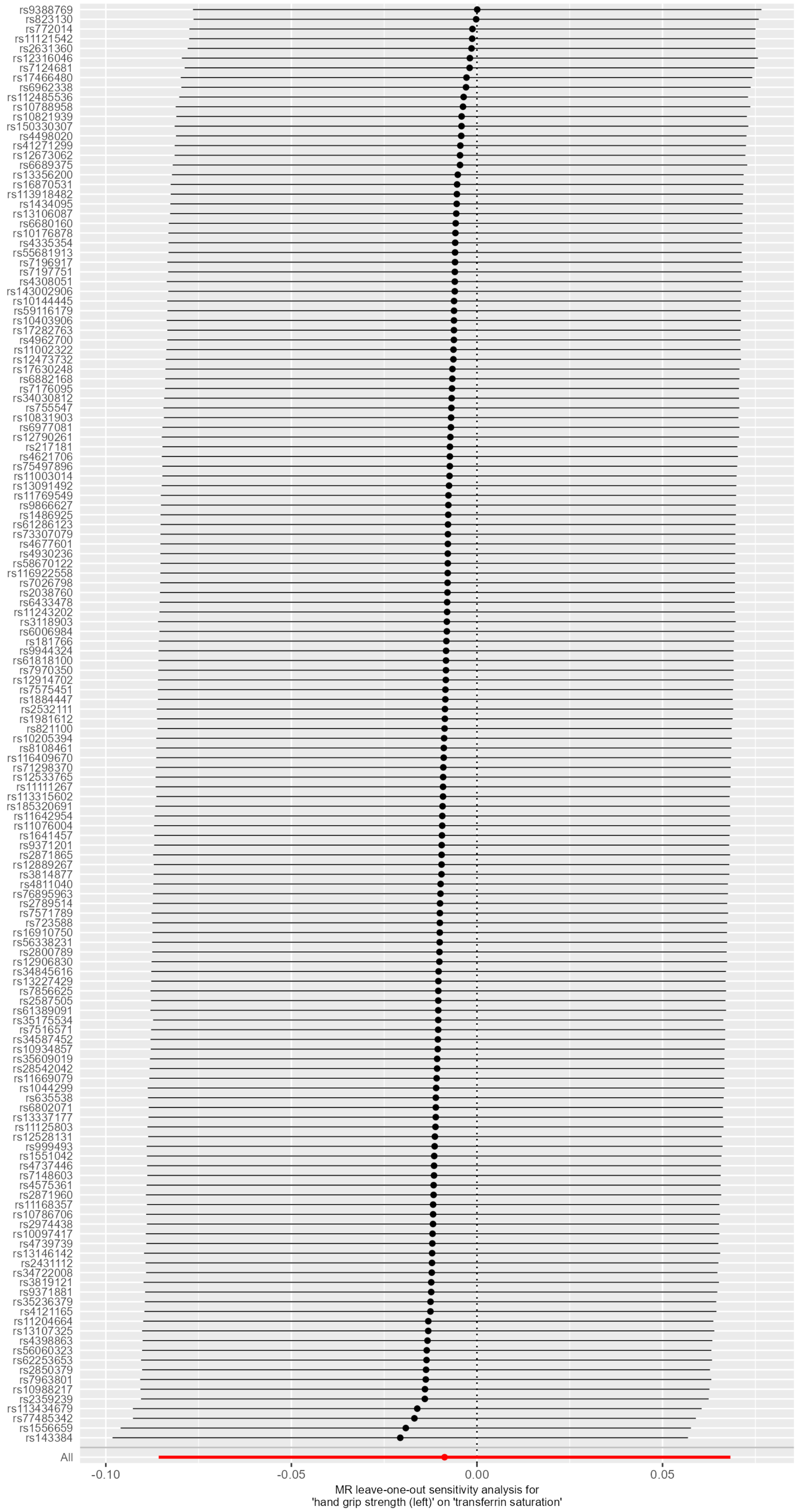
rs9388769
rs823130
rs772014
rs11121542
rs2631360
rs12316046
rs7124681
rs17466480
rs6962338
rs112485536
rs10788958
rs10821939
rs150330307
rs4498020
rs41271299
rs12673062
rs6689375
rs13356200
rs16870531
rs113918482
rs1434095
rs13106087
rs6680160
rs10176878
rs4335354
rs55681913
rs7196917
rs7197751
rs4308051
rs143002906
rs10144445
rs59116179
rs10403906
rs17282763
rs4962700
rs11002322
rs12473732
rs17630248
rs6882168
rs7176095
rs34030812
rs755547
rs10831903
rs6977081
rs12790261
rs217181
rs4621706
rs75497896
rs11003014
rs13091492
rs11769549
rs9866627
rs1486925
rs61286123
rs73307079
rs4677601
rs4930236
rs58670122
rs116922558
rs7026798
rs2038760
rs6433478
rs11243202
rs3118903
rs6006984
rs181766
rs9944324
rs61818100
rs7970350
rs12914702
rs7575451
rs1884447
rs2532111
rs1981612
rs821100
rs10205394
rs8108461
rs116409670
rs71298370
rs12533765
rs11111267
rs113315602
rs185320691
rs11642954
rs11076004
rs1641457
rs9371201
rs2871865
rs12889267
rs3814877
rs4811040
rs76895963
rs2789514
rs7571789
rs723588
rs16910750
rs56338231
rs2800789
rs12906830
rs34845616
rs13227429
rs7856625
rs2587505
rs61389091
rs35175534
rs7516571
rs34587452
rs10934857
rs35609019
rs28542042
rs11669079
rs1044299
rs635538
rs6802071
rs13337177
rs11125803
rs12528131
rs999493
rs1551042
rs4737446
rs71486603
rs4575361
rs2871960
rs11168357
rs10786706
rs2974438
rs10097417
rs4739739
rs13146142
rs2431112
rs34722008
rs3819121
rs9371881
rs35236379
rs4121165
rs11204664
rs13107325
rs4398863
rs56060323
rs62253653
rs2850379
rs7963801
rs10988217
rs2359239
rs113434679
rs77485342
rs1556659
rs143384
All
-0.10
-0.05
0.00
0.05
MR leave-one-out sensitivity analysis for
'hand grip strength (left)' on 'transferrin saturation'

Supplementary Figure 28. Leave-one-out plot for sensitivity analysis of single SNP effect on "hand grip strength (left)"-to-"transferrin saturation" UVMR results.

Leave-one-out plot using IVW method by sequentially re-evaluating the causal estimate after discarding one SNP at a time, which helps determine whether the overall effect is driven by the specific genetic variant. The black point denotes the causal effect estimate of hand grip strength (left) on transferrin saturation after discarding a certain SNP, and the black line signifies the 95% CI of estimate. The red point symbolizes the causal effect estimate of hand grip strength (left) on transferrin saturation, and the red line indicates the 95% CI of the estimate. **Abbreviations:** SNP = number of single-nucleotide polymorphism; UVMR = univariate Mendelian randomization; CI = confidence interval.

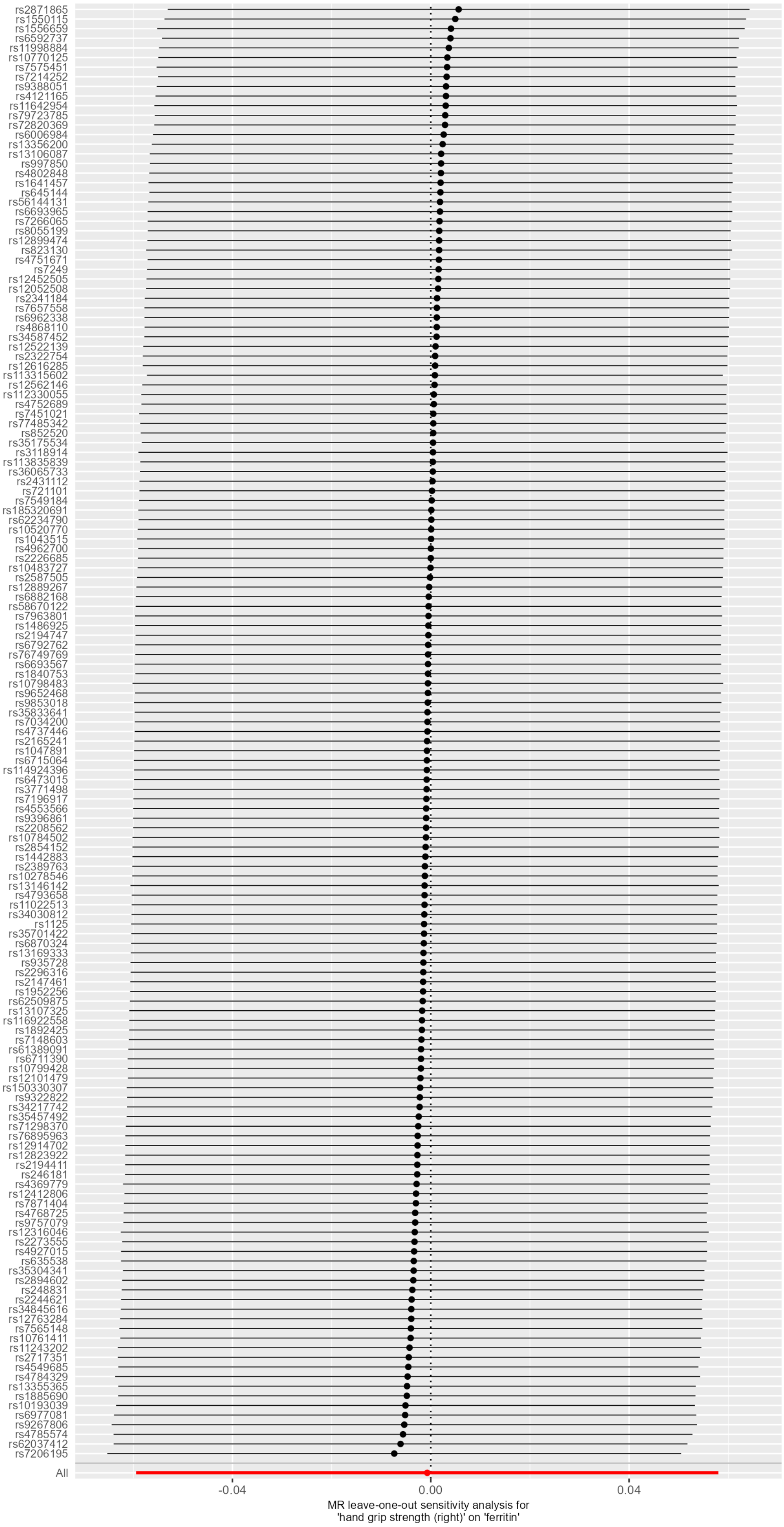
rs2871865
rs1550115
rs1556659
rs6592737
rs11998884
rs10770125
rs7575451
rs7214252
rs9388051
rs4121165
rs11642954
rs79723785
rs72820369
rs6006984
rs13356200
rs13106087
rs997850
rs4802848
rs1641457
rs645144
rs56144131
rs6693965
rs7266065
rs8055199
rs12899474
rs823130
rs4751671
rs7249
rs12452505
rs12052508
rs2341184
rs7657558
rs6962338
rs4868110
rs34587452
rs12522139
rs2322754
rs12616285
rs113315602
rs12562146
rs112330055
rs4752689
rs7451021
rs77485342
rs852520
rs35175534
rs3118914
rs113835839
rs36065733
rs2431112
rs721101
rs7549184
rs185320691
rs62234790
rs10520770
rs1043515
rs4962700
rs2226685
rs10483727
rs2587505
rs12889267
rs6882168
rs58670122
rs7963801
rs1486925
rs2194747
rs6792762
rs76749769
rs6693567
rs1840753
rs10798483
rs9652468
rs9853018
rs35833641
rs7034200
rs4737446
rs2165241
rs1047891
rs6715064
rs114924396
rs6473015
rs3771498
rs7196917
rs4553566
rs9396861
rs2208562
rs10784502
rs2854152
rs1442883
rs2389763
rs10278546
rs13146142
rs4793658
rs11022513
rs34030812
rs1125
rs35701422
rs6870324
rs13169333
rs935728
rs2296316
rs2147461
rs1952256
rs62509875
rs13107325
rs116922558
rs1892425
rs7148603
rs61389091
rs6711390
rs10799428
rs12101479
rs150330307
rs9322822
rs34217742
rs35457492
rs71298370
rs76895963
rs12914702
rs12823922
rs2194411
rs246181
rs4369779
rs12412806
rs7871404
rs4768725
rs9757079
rs12316046
rs2273555
rs4927015
rs635538
rs35304341
rs2894602
rs248831
rs2244621
rs34845616
rs12763284
rs7565148
rs10761411
rs11243202
rs2717351
rs45496685
rs4784329
rs13355365
rs1885690
rs10193039
rs6977081
rs9267806
rs4785574
rs62037412
rs7206195
All
-0.04
0.00
0.04
MR leave-one-out sensitivity analysis for
'hand grip strength (right)' on 'ferritin'

Supplementary Figure 29. Leave-one-out plot for sensitivity analysis of single SNP effect on "hand grip strength (right)"-to-"ferritin" UVMR results.

Leave-one-out plot using IVW method by sequentially re-evaluating the causal estimate after discarding one SNP at a time, which helps determine whether the overall effect is driven by the specific genetic variant. The black point denotes the causal effect estimate of hand grip strength (right) on ferritin after discarding a certain SNP, and the black line signifies the 95% CI of estimate. The red point symbolizes the causal effect estimate of hand grip strength (right) on ferritin, and the red line indicates the 95% CI of the estimate. **Abbreviations:** SNP = number of single-nucleotide polymorphism; UVMR = univariate Mendelian randomization; CI = confidence interval.

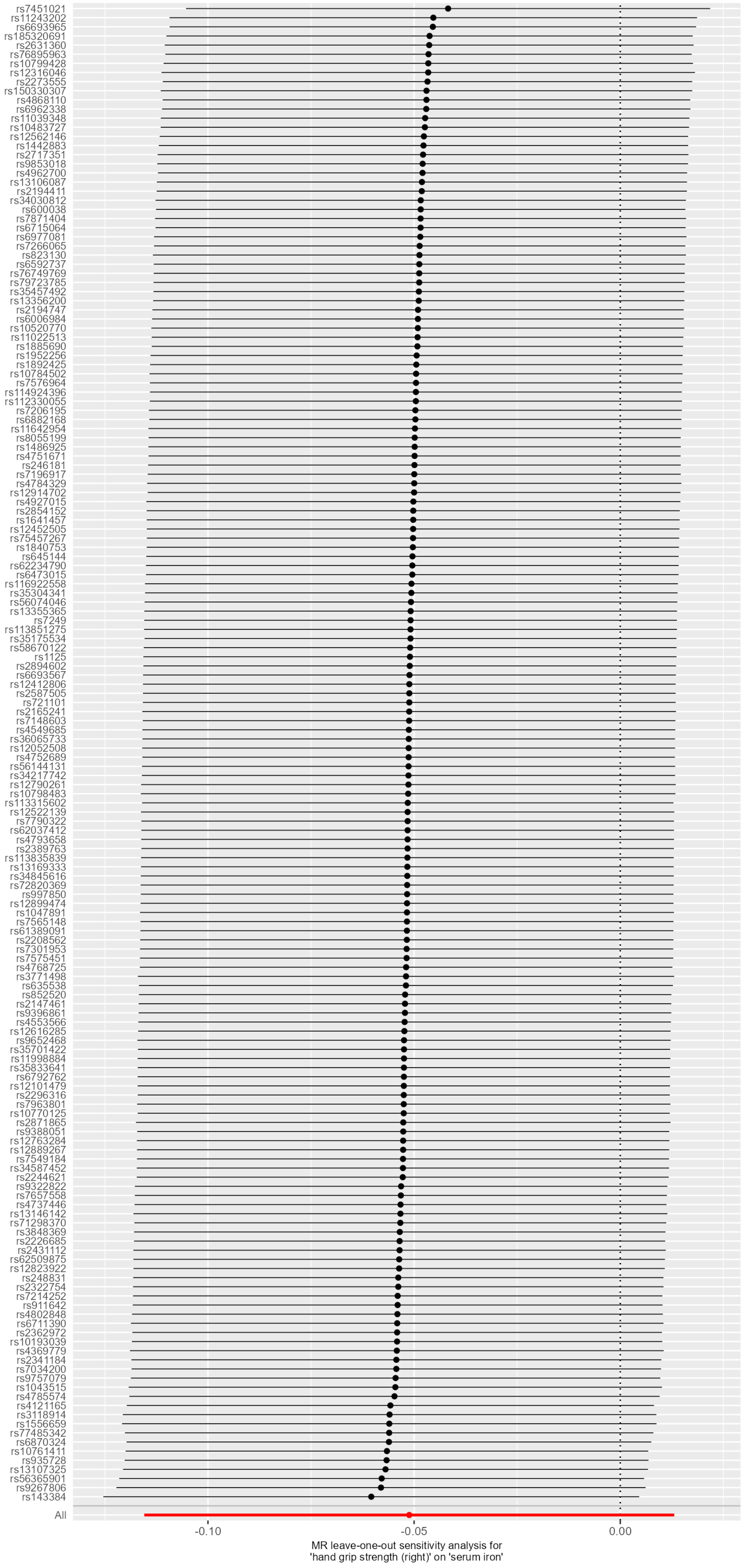
rs7451021
rs11243202
rs6693965
rs185320691
rs2631360
rs76895963
rs10799428
rs12316046
rs2273555
rs150330307
rs4868110
rs6962338
rs11039348
rs10483727
rs12562146
rs1442883
rs2717351
rs9853018
rs4962700
rs13106087
rs2194411
rs34030812
rs600038
rs7871404
rs6715064
rs6977081
rs7266065
rs823130
rs6592737
rs76749769
rs79723785
rs35457492
rs13356200
rs2194747
rs6006984
rs10520770
rs11022513
rs1885690
rs1952256
rs1892425
rs10784502
rs7576964
rs114924396
rs112330055
rs7206195
rs6882168
rs11642954
rs8055199
rs1486925
rs4751671
rs246181
rs7196917
rs4784329
rs12914702
rs4927015
rs2854152
rs1641457
rs12452505
rs75457267
rs1840753
rs645144
rs62234790
rs6473015
rs116922558
rs35304341
rs56074046
rs13355365
rs7249
rs113851275
rs35175534
rs58670122
rs1125
rs2894602
rs6693567
rs12412806
rs2587505
rs721101
rs2165241
rs7148603
rs4549685
rs36065733
rs12052508
rs4752689
rs56144131
rs34217742
rs12790261
rs10798483
rs113315602
rs12522139
rs7790322
rs62037412
rs4793658
rs2389763
rs113835839
rs13169333
rs34845616
rs72820369
rs997850
rs12899474
rs1047891
rs7565148
rs61389091
rs2208562
rs7301953
rs7575451
rs4768725
rs3771498
rs635538
rs852520
rs2147461
rs9396861
rs4553566
rs12616285
rs9652468
rs35701422
rs11998884
rs35833641
rs6792762
rs12101479
rs2296316
rs7963801
rs10770125
rs2871865
rs9388051
rs12763284
rs12889267
rs7549184
rs34587452
rs2244621
rs9322822
rs7657558
rs4737446
rs13146142
rs71298370
rs3848369
rs2226685
rs2431112
rs62509875
rs12823922
rs248831
rs2322754
rs7214252
rs911642
rs4802848
rs6711390
rs2362972
rs10193039
rs4369779
rs2341184
rs7034200
rs9757079
rs1043515
rs4785574
rs4121165
rs3118914
rs1556659
rs77485342
rs6870324
rs10761411
rs935728
rs13107325
rs56365901
rs9267806
rs143384
All
-0.10
-0.05
0.00
MR leave-one-out sensitivity analysis for
'hand grip strength (right)' on 'serum iron'

Supplementary Figure 30. Leave-one-out plot for sensitivity analysis of single SNP effect on "hand grip strength (right)"-to-"serum iron" UVMR results.

Leave-one-out plot using IVW method by sequentially re-evaluating the causal estimate after discarding one SNP at a time, which helps determine whether the overall effect is driven by the specific genetic variant. The black point denotes the causal effect estimate of hand grip strength (right) on serum iron after discarding a certain SNP, and the black line signifies the 95% CI of estimate. The red point symbolizes the causal effect estimate of hand grip strength (right) on serum iron, and the red line indicates the 95% CI of the estimate. **Abbreviations:** SNP = number of single-nucleotide polymorphism; UVMR = univariate Mendelian randomization; CI = confidence interval.

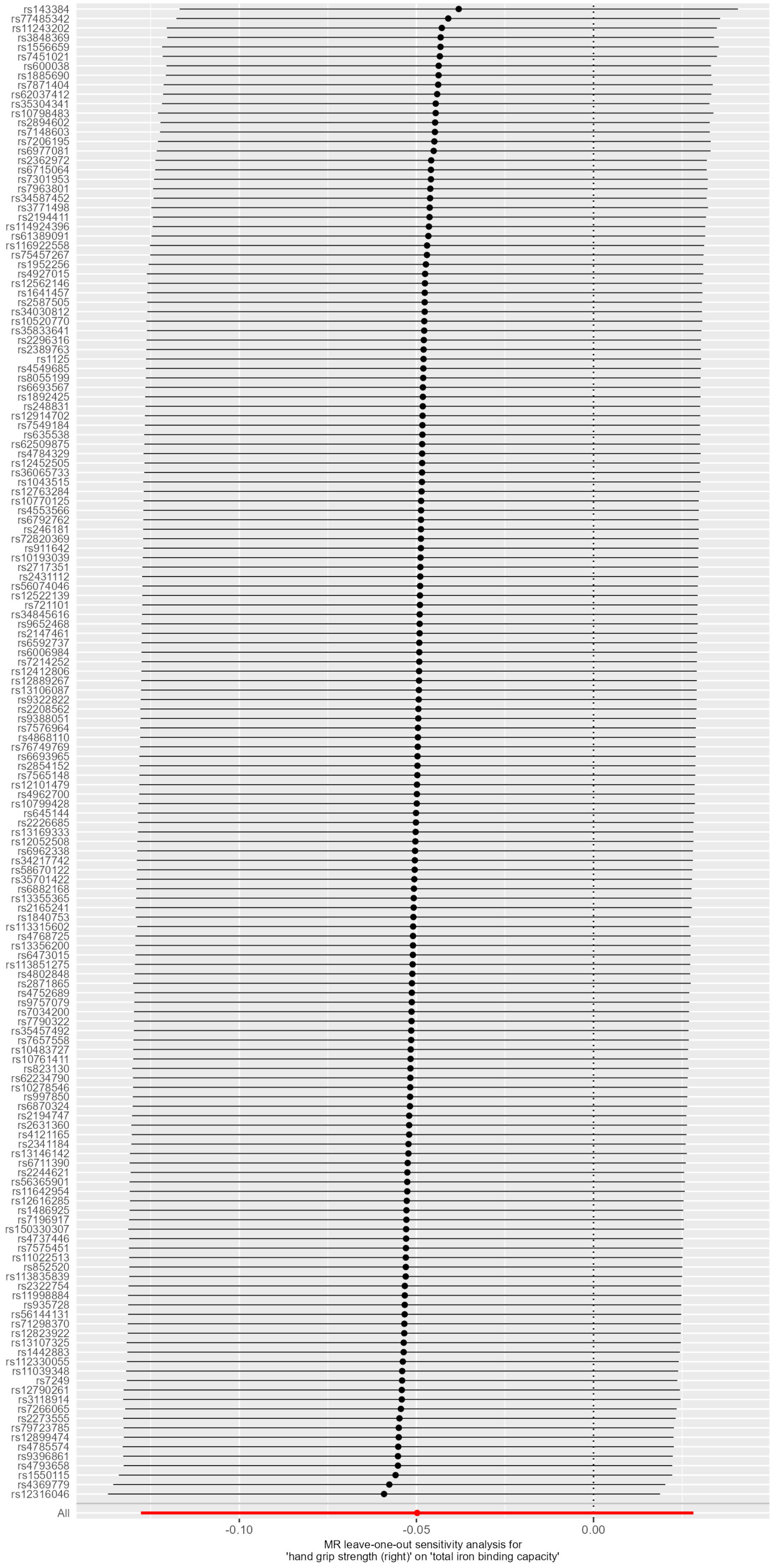
rs143384
rs77485342
rs11243202
rs3848369
rs1556659
rs7451021
rs600038
rs1885690
rs7871404
rs62037412
rs35304341
rs10798483
rs2894602
rs7148603
rs7206195
rs6977081
rs2362972
rs6715064
rs7301953
rs7963801
rs34587452
rs3771498
rs2194411
rs114924396
rs61389091
rs116922558
rs75457267
rs1952256
rs4927015
rs12562146
rs1641457
rs2587505
rs34030812
rs10520770
rs35833641
rs2296316
rs2389763
rs1125
rs4549685
rs8055199
rs6693567
rs1892425
rs248831
rs12914702
rs7549184
rs635538
rs62509875
rs4784329
rs12452505
rs36065733
rs1043515
rs12763284
rs10770125
rs4553566
rs6792762
rs246181
rs72820369
rs911642
rs10193039
rs2717351
rs2431112
rs56074046
rs12522139
rs721101
rs34845616
rs9652468
rs2147461
rs6592737
rs6006984
rs7214252
rs12412806
rs12889267
rs13106087
rs9322822
rs2208562
rs9388051
rs7576964
rs4868110
rs76749769
rs6693965
rs2854152
rs7565148
rs12101479
rs4962700
rs10799428
rs645144
rs2226685
rs13169333
rs12052508
rs6962338
rs34217742
rs58670122
rs35701422
rs6882168
rs13355365
rs2165241
rs1840753
rs113315602
rs4768725
rs13356200
rs6473015
rs113851275
rs4802848
rs2871865
rs4752689
rs9757079
rs7034200
rs7790322
rs35457492
rs7657558
rs10483727
rs10761411
rs823130
rs62234790
rs10278546
rs997850
rs6870324
rs2194747
rs2631360
rs4121165
rs2341184
rs13146142
rs6711390
rs2244621
rs56365901
rs11642954
rs12616285
rs1486925
rs7196917
rs150330307
rs4737446
rs7575451
rs11022513
rs852520
rs113835839
rs2322754
rs11998884
rs935728
rs56144131
rs71298370
rs12823922
rs13107325
rs1442883
rs112330055
rs11039348
rs7249
rs12790261
rs3118914
rs7266065
rs2273555
rs79723785
rs12899474
rs4785574
rs9396861
rs4793658
rs1550115
rs4369779
rs12316046
All
-0.10
-0.05
0.00
MR leave-one-out sensitivity analysis for
'hand grip strength (right)' on 'total iron binding capacity'

Supplementary Figure 31. Leave-one-out plot for sensitivity analysis of single SNP effect on "hand grip strength (right)"-to-"total iron binding capacity" UVMR results.

Leave-one-out plot using IVW method by sequentially re-evaluating the causal estimate after discarding one SNP at a time, which helps determine whether the overall effect is driven by the specific genetic variant. The black point denotes the causal effect estimate of hand grip strength (right) on total iron binding capacity after discarding a certain SNP, and the black line signifies the 95% CI of estimate. The red point symbolizes the causal effect estimate of hand grip strength (right) on total iron binding capacity, and the red line indicates the 95% CI of the estimate. **Abbreviations:** SNP = number of single-nucleotide polymorphism; UVMR = univariate Mendelian randomization; CI = confidence interval.

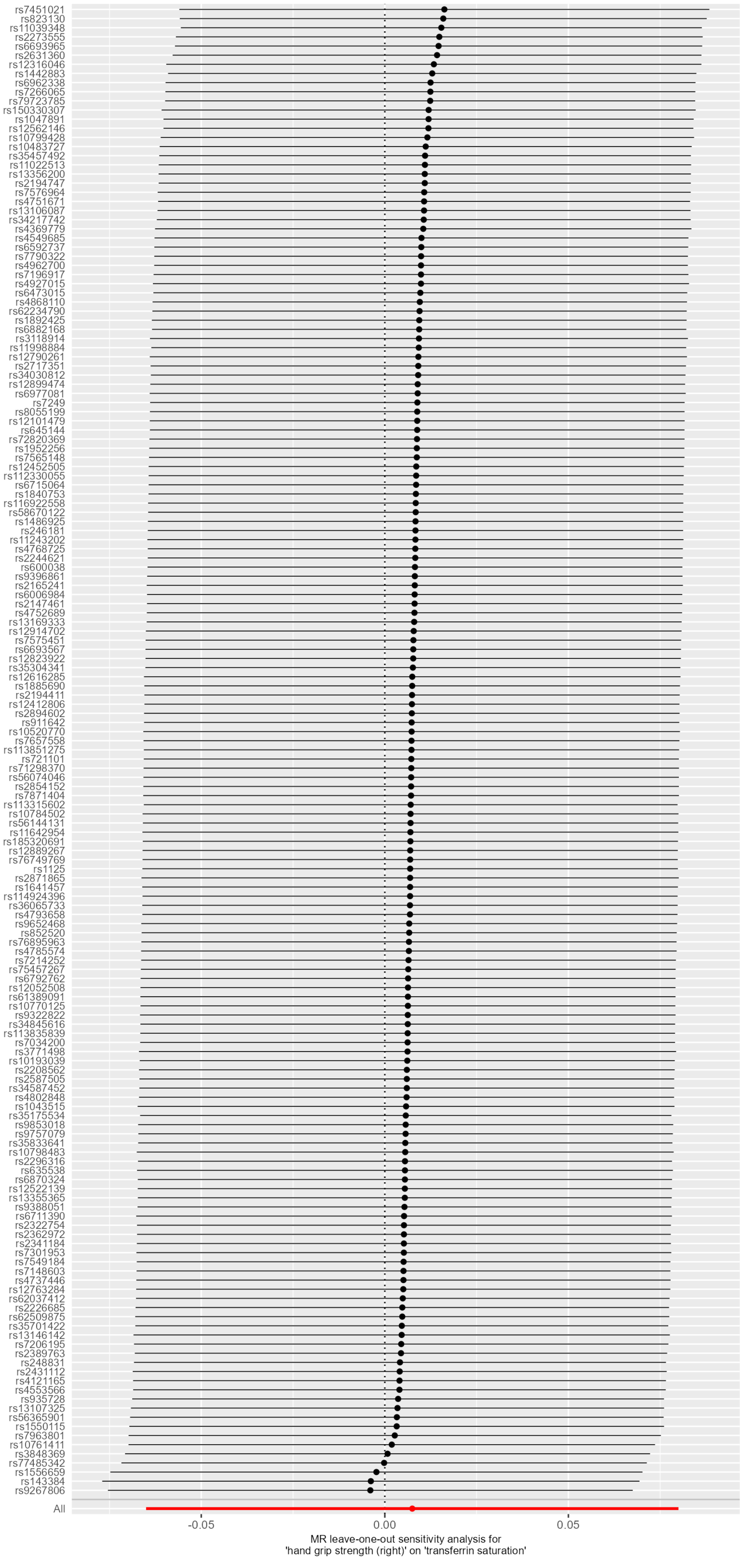
rs7451021
rs823130
rs11039348
rs2273555
rs6693965
rs2631360
rs12316046
rs1442883
rs6962338
rs7266065
rs79723785
rs150330307
rs1047891
rs12562146
rs10799428
rs10483727
rs35457492
rs11022513
rs13356200
rs2194747
rs7576964
rs4751671
rs13106087
rs34217742
rs4369779
rs4549685
rs6592737
rs7790322
rs4962700
rs7196917
rs4927015
rs6473015
rs4868110
rs62234790
rs1892425
rs6882168
rs3118914
rs11998884
rs12790261
rs2717351
rs34030812
rs12899474
rs6977081
rs7249
rs8055199
rs12101479
rs645144
rs72820369
rs1952256
rs7565148
rs12452505
rs112330055
rs6715064
rs1840753
rs116922558
rs58670122
rs1486925
rs246181
rs11243202
rs4768725
rs2244621
rs600038
rs9396861
rs2165241
rs6006984
rs2147461
rs4752689
rs13169333
rs12914702
rs7575451
rs6693567
rs12823922
rs35304341
rs12616285
rs1885690
rs2194411
rs12412806
rs2894602
rs911642
rs10520770
rs7657558
rs113851275
rs721101
rs71298370
rs56074046
rs2854152
rs7871404
rs113315602
rs10784502
rs56144131
rs11642954
rs185320691
rs12889267
rs76749769
rs1125
rs2871865
rs1641457
rs114924396
rs36065733
rs4793658
rs9652468
rs852520
rs76895963
rs4785574
rs7214252
rs75457267
rs6792762
rs12052508
rs61389091
rs10770125
rs9322822
rs34845616
rs113835839
rs7034200
rs3771498
rs10193039
rs2208562
rs2587505
rs34587452
rs4802848
rs1043515
rs35175534
rs9853018
rs9757079
rs35833641
rs10798483
rs2296316
rs635538
rs6870324
rs12522139
rs13355365
rs9388051
rs6711390
rs2322754
rs2362972
rs2341184
rs7301953
rs7549184
rs7148603
rs4737446
rs12763284
rs62037412
rs2226685
rs62509875
rs35701422
rs13146142
rs7206195
rs2389763
rs248831
rs2431112
rs4121165
rs4553566
rs935728
rs13107325
rs56365901
rs1550115
rs7963801
rs10761411
rs3848369
rs77485342
rs1556659
rs143384
rs9267806
All
-0.05
0.00
0.05
MR leave-one-out sensitivity analysis for
'hand grip strength (right)' on 'transferrin saturation'

Supplementary Figure 32. Leave-one-out plot for sensitivity analysis of single SNP effect on "hand grip strength (right)"-to-"transferrin saturation" UVMR results.

Leave-one-out plot using IVW method by sequentially re-evaluating the causal estimate after discarding one SNP at a time, which helps determine whether the overall effect is driven by the specific genetic variant. The black point denotes the causal effect estimate of hand grip strength (right) on transferrin saturation after discarding a certain SNP, and the black line signifies the 95% CI of estimate. The red point symbolizes the causal effect estimate of hand grip strength (right) on transferrin saturation, and the red line indicates the 95% CI of the estimate. **Abbreviations:** SNP = number of single-nucleotide polymorphism; UVMR = univariate Mendelian randomization; CI = confidence interval.

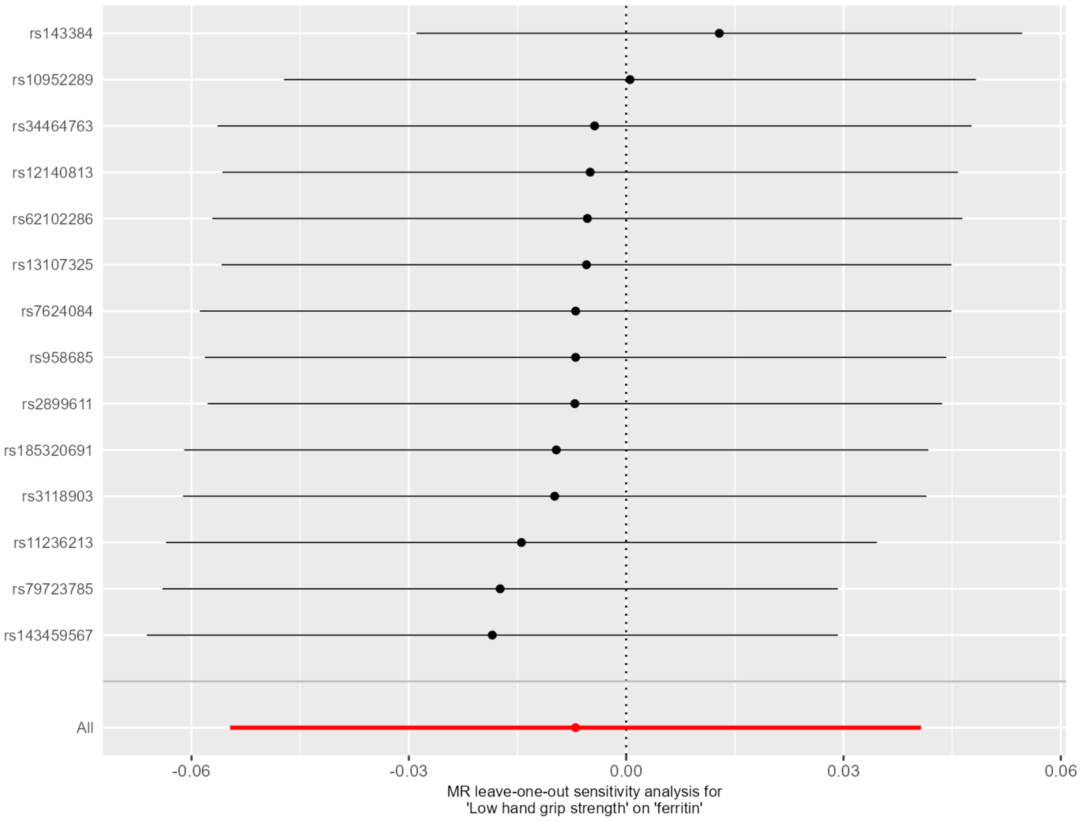

Supplementary Figure 33. Leave-one-out plot for sensitivity analysis of single SNP effect on "low hand grip strength"-to-"ferritin" UVMR results.

Leave-one-out plot using IVW method by sequentially re-evaluating the causal estimate after discarding one SNP at a time, which helps determine whether the overall effect is driven by the specific genetic variant. The black point denotes the causal effect estimate of low hand grip strength on ferritin after discarding a certain SNP, and the black line signifies the 95% CI of estimate. The red point symbolizes the causal effect estimate of low hand grip strength on ferritin, and the red line indicates the 95% CI of the estimate. **Abbreviations:** SNP = number of single-nucleotide polymorphism; UVMR = univariate Mendelian randomization; CI = confidence interval.

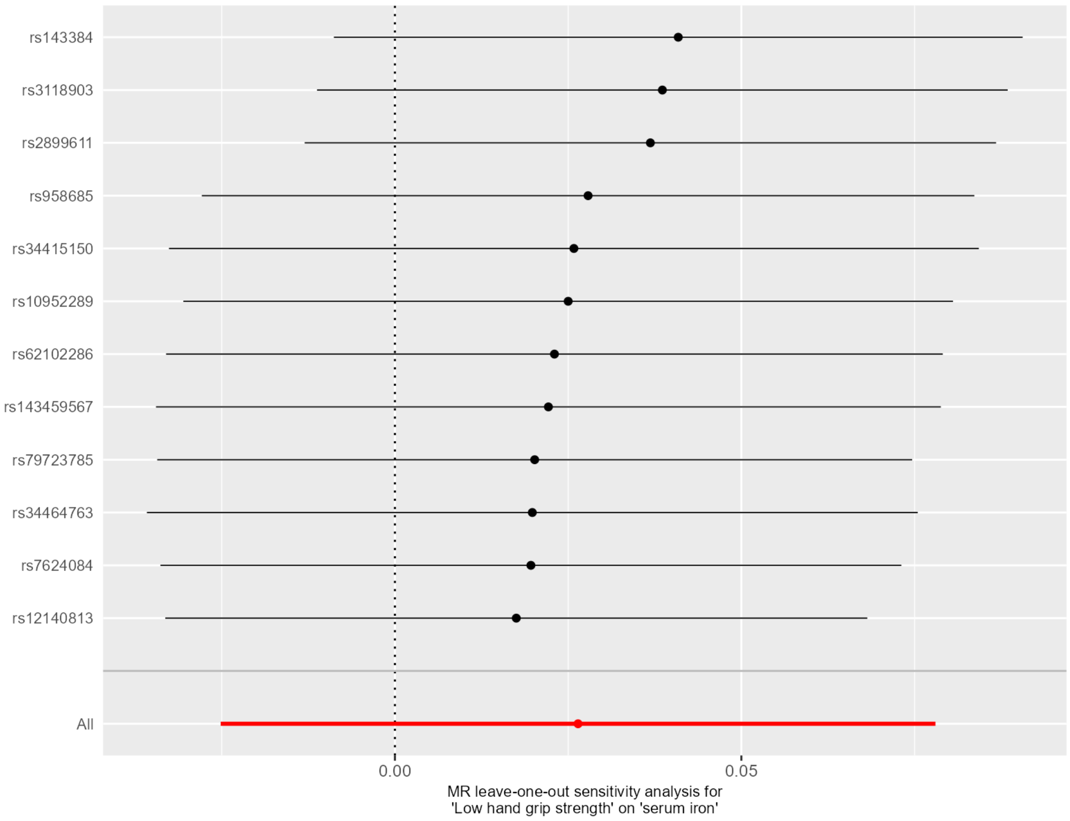

Supplementary Figure 34. Leave-one-out plot for sensitivity analysis of single SNP effect on "low hand grip strength"-to-"serum iron" UVMR results.

Leave-one-out plot using IVW method by sequentially re-evaluating the causal estimate after discarding one SNP at a time, which helps determine whether the overall effect is driven by the specific genetic variant. The black point denotes the causal effect estimate of low hand grip strength on serum iron after discarding a certain SNP, and the black line signifies the 95% CI of estimate. The red point symbolizes the causal effect estimate of low hand grip strength on serum iron, and the red line indicates the 95% CI of the estimate. **Abbreviations:** SNP = number of single-nucleotide polymorphism; UVMR = univariate Mendelian randomization; CI = confidence interval.

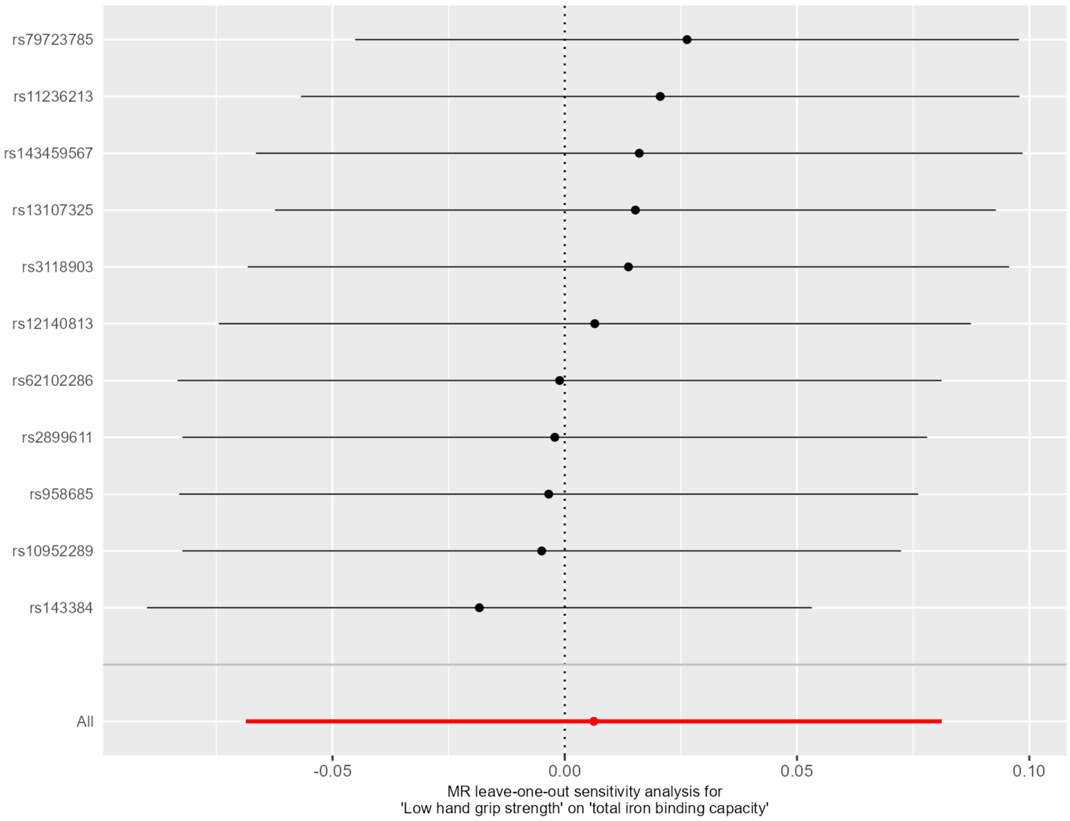

Supplementary Figure 35. Leave-one-out plot for sensitivity analysis of single SNP effect on "low hand grip strength"-to-"total iron binding capacity" UVMR results.

Leave-one-out plot using IVW method by sequentially re-evaluating the causal estimate after discarding one SNP at a time, which helps determine whether the overall effect is driven by the specific genetic variant. The black point denotes the causal effect estimate of low hand grip strength on total iron binding capacity after discarding a certain SNP, and the black line signifies the 95% CI of estimate. The red point symbolizes the causal effect estimate of low hand grip strength on total iron binding capacity, and the red line indicates the 95% CI of the estimate. **Abbreviations:** SNP = number of single-nucleotide polymorphism; UVMR = univariate Mendelian randomization; CI = confidence interval.

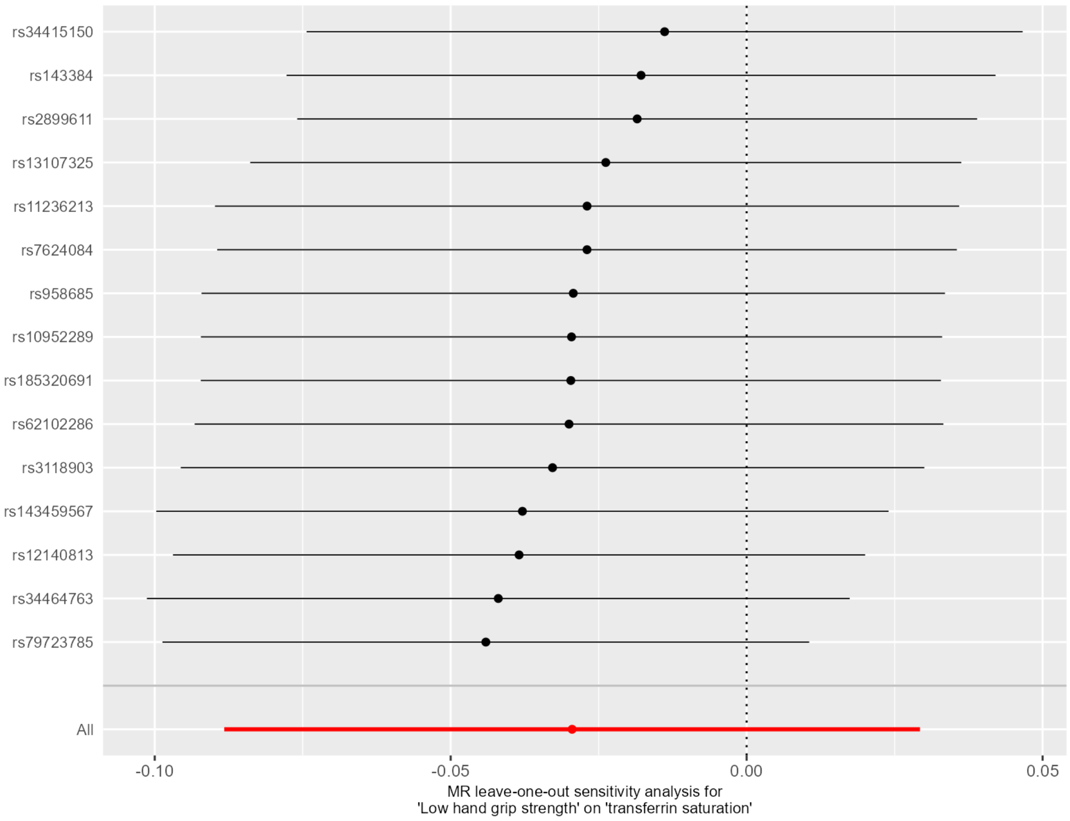

Supplementary Figure 36. Leave-one-out plot for sensitivity analysis of single SNP effect on "low hand grip strength"-to-"transferrin saturation" UVMR results.

Leave-one-out plot using IVW method by sequentially re-evaluating the causal estimate after discarding one SNP at a time, which helps determine whether the overall effect is driven by the specific genetic variant. The black point denotes the causal effect estimate of low hand grip strength on transferrin saturation after discarding a certain SNP, and the black line signifies the 95% CI of estimate. The red point symbolizes the causal effect estimate of low hand grip strength on transferrin saturation, and the red line indicates the 95% CI of the estimate. **Abbreviations:** SNP = number of single-nucleotide polymorphism; UVMR = univariate Mendelian randomization; CI = confidence interval.

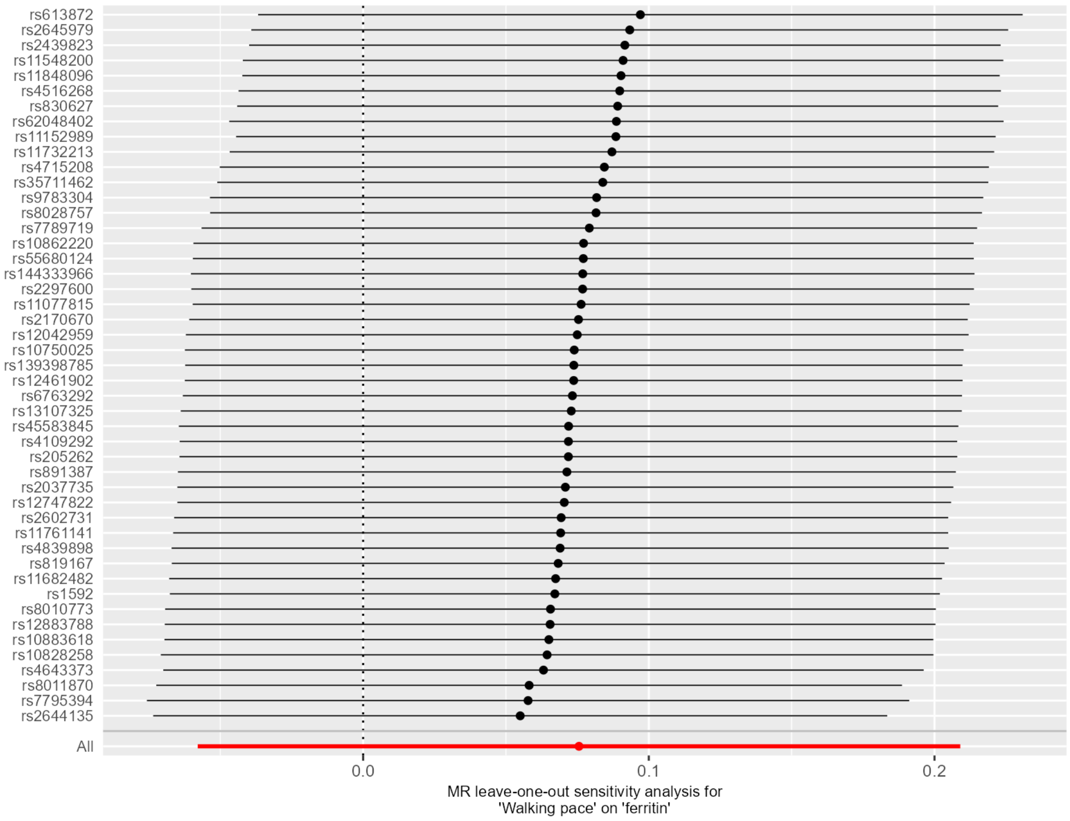

Supplementary Figure 37. Leave-one-out plot for sensitivity analysis of single SNP effect on "walking pace"-to-"ferritin" UVMR results.

Leave-one-out plot using IVW method by sequentially re-evaluating the causal estimate after discarding one SNP at a time, which helps determine whether the overall effect is driven by the specific genetic variant. The black point denotes the causal effect estimate of walking pace on ferritin after discarding a certain SNP, and the black line signifies the 95% CI of estimate. The red point symbolizes the causal effect estimate of walking pace on ferritin, and the red line indicates the 95% CI of the estimate. **Abbreviations:** SNP = number of single-nucleotide polymorphism; UVMR = univariate Mendelian randomization; CI = confidence interval.

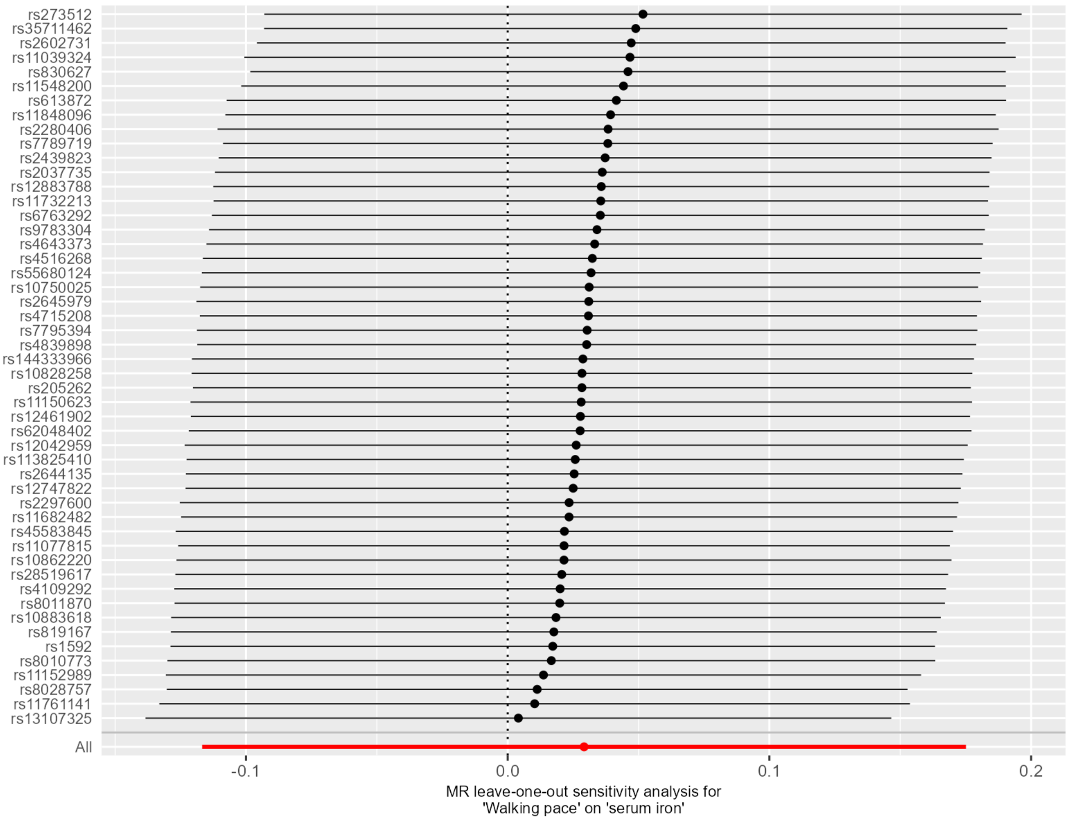

Supplementary Figure 38. Leave-one-out plot for sensitivity analysis of single SNP effect on "walking pace"-to-"serum iron" UVMR results.

Leave-one-out plot using IVW method by sequentially re-evaluating the causal estimate after discarding one SNP at a time, which helps determine whether the overall effect is driven by the specific genetic variant. The black point denotes the causal effect estimate of walking pace on serum iron after discarding a certain SNP, and the black line signifies the 95% CI of estimate. The red point symbolizes the causal effect estimate of walking pace on serum iron, and the red line indicates the 95% CI of the estimate. **Abbreviations:** SNP = number of single-nucleotide polymorphism; UVMR = univariate Mendelian randomization; CI = confidence interval.

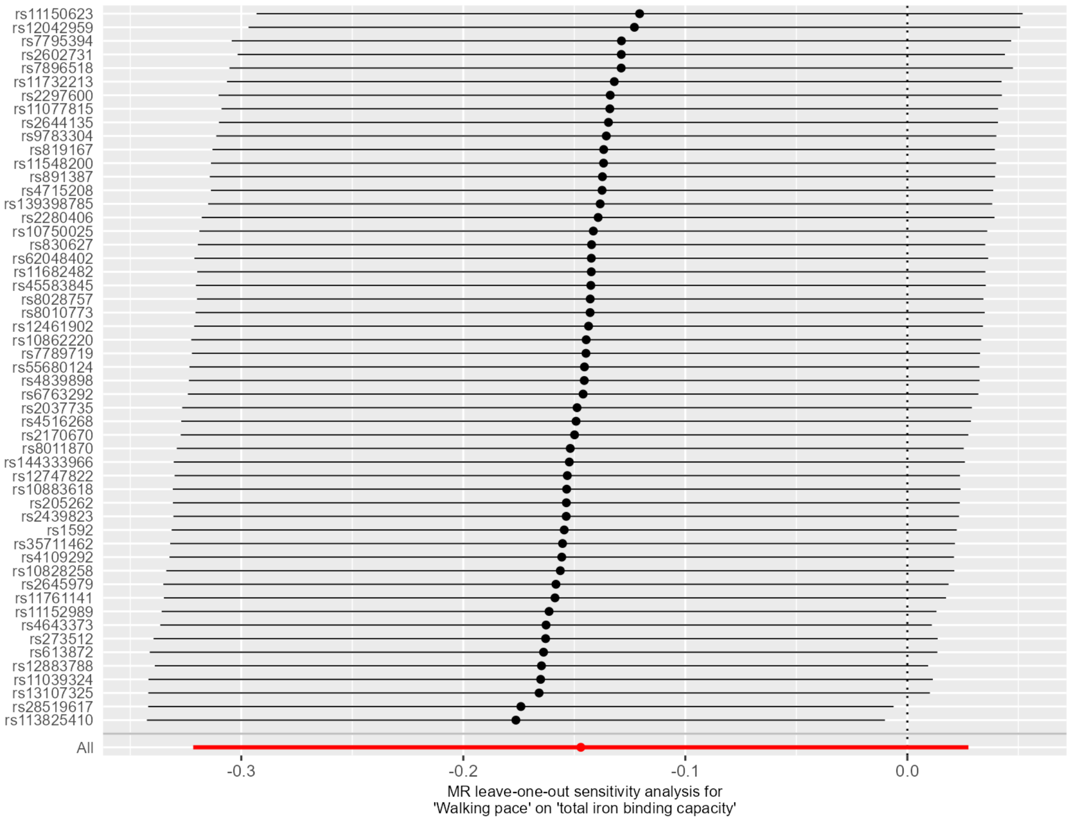

Supplementary Figure 39. Leave-one-out plot for sensitivity analysis of single SNP effect on "walking pace"-to-"total iron binding capacity" UVMR results.

Leave-one-out plot using IVW method by sequentially re-evaluating the causal estimate after discarding one SNP at a time, which helps determine whether the overall effect is driven by the specific genetic variant. The black point denotes the causal effect estimate of walking pace on total iron binding capacity after discarding a certain SNP, and the black line signifies the 95% CI of estimate. The red point symbolizes the causal effect estimate of walking pace on total iron binding capacity, and the red line indicates the 95% CI of the estimate. **Abbreviations:** SNP = number of single-nucleotide polymorphism; UVMR = univariate Mendelian randomization; CI = confidence interval.

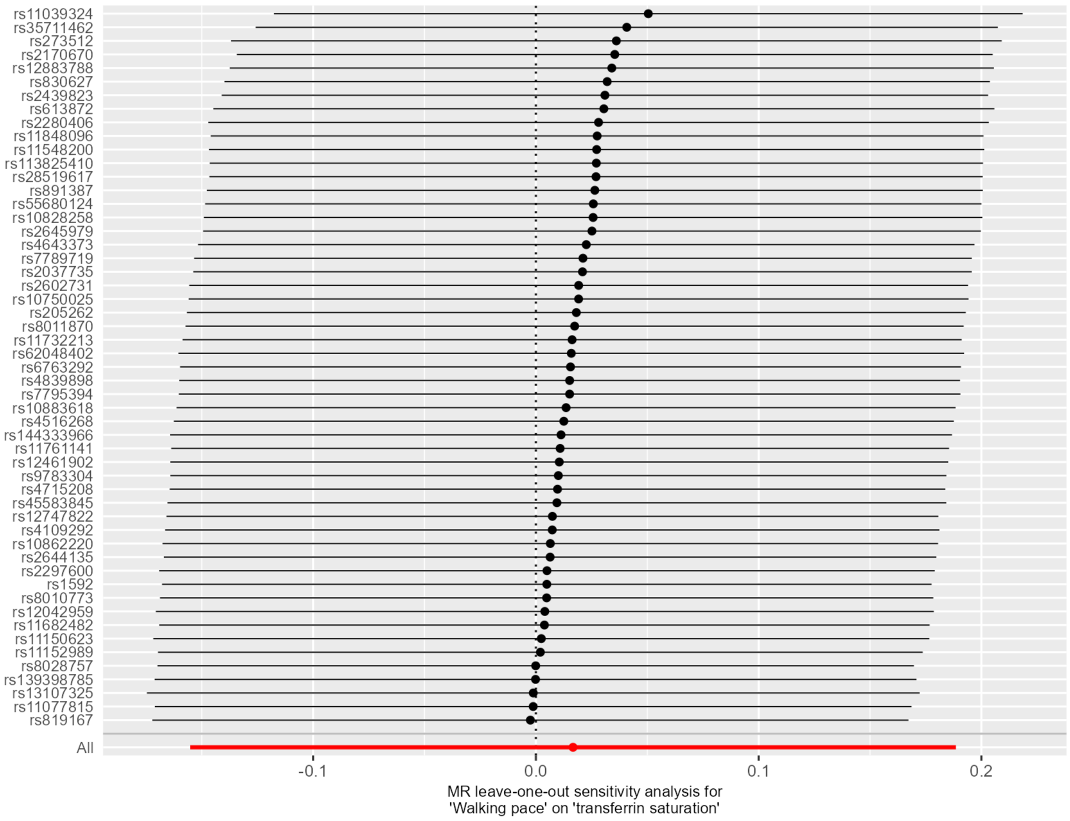

Supplementary Figure 40. Leave-one-out plot for sensitivity analysis of single SNP effect on "walking pace"-to-"transferrin saturation" UVMR results.

Leave-one-out plot using IVW method by sequentially re-evaluating the causal estimate after discarding one SNP at a time, which helps determine whether the overall effect is driven by the specific genetic variant. The black point denotes the causal effect estimate of walking pace on transferrin saturation after discarding a certain SNP, and the black line signifies the 95% CI of estimate. The red point symbolizes the causal effect estimate of walking pace on transferrin saturation, and the red line indicates the 95% CI of the estimate. **Abbreviations:** SNP = number of single-nucleotide polymorphism; UVMR = univariate Mendelian randomization; CI = confidence interval.

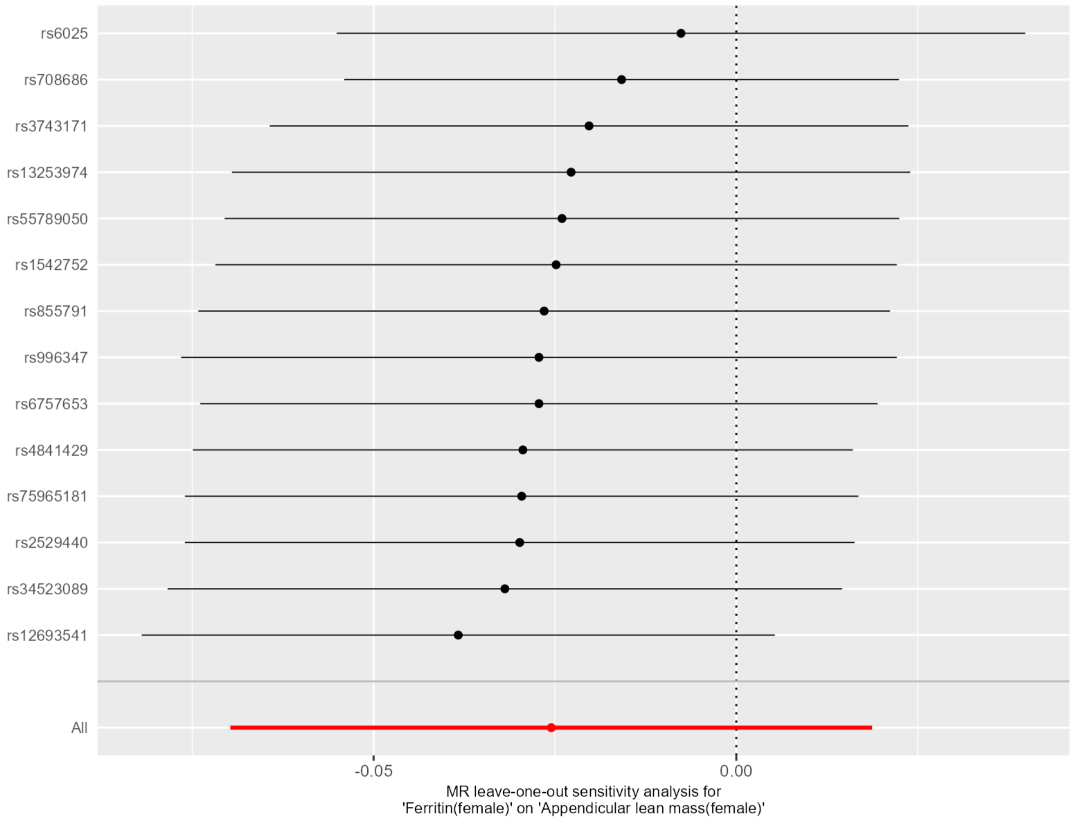

Supplementary Figure 41. Leave-one-out plot for sensitivity analysis of single SNP effect on “ferritin(female)”-to-“appendicular lean mass(female)” UVMR results.

Leave-one-out plot using IVW method by sequentially re-evaluating the causal estimate after discarding one SNP at a time, which helps determine whether the overall effect is driven by the specific genetic variant. The black point denotes the causal effect estimate of ferritin on appendicular lean mass after discarding a certain SNP, and the black line signifies the 95% CI of estimate. The red point symbolizes the causal effect estimate of ferritin on appendicular lean mass, and the red line indicates the 95% CI of the estimate. **Abbreviations:** SNP = number of single-nucleotide polymorphism; UVMR = univariate Mendelian randomization; CI = confidence interval.

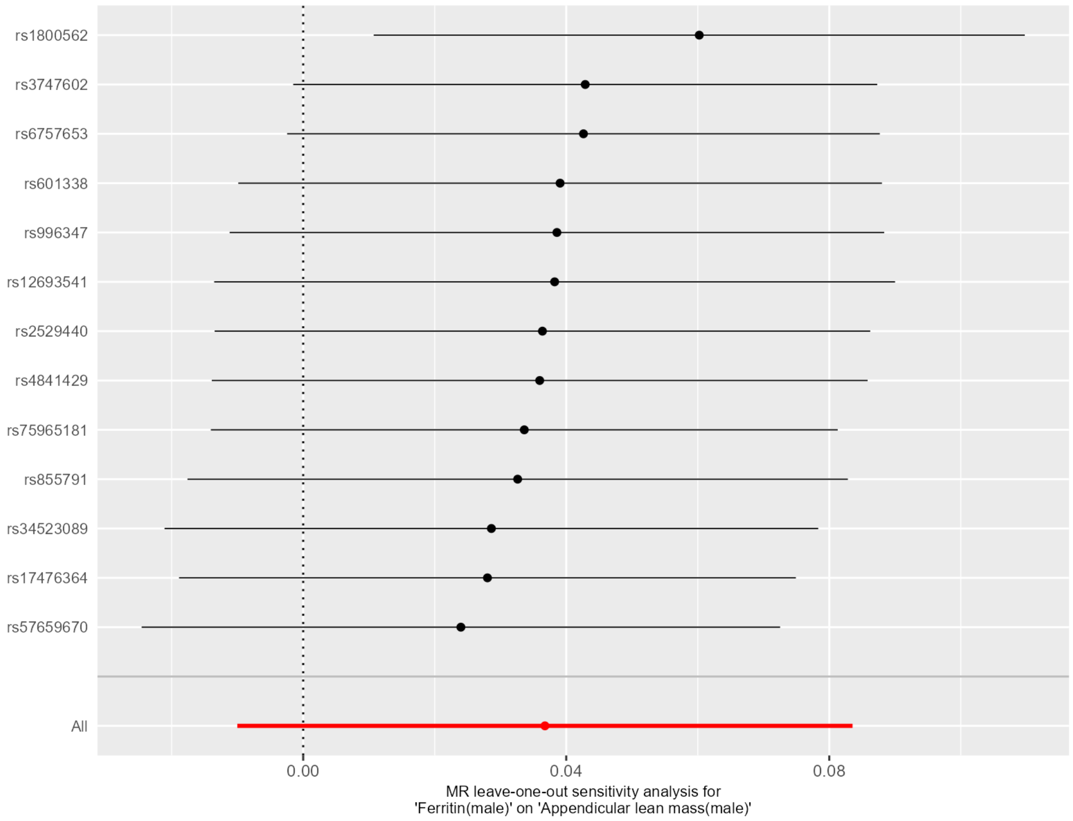

Supplementary Figure 42. Leave-one-out plot for sensitivity analysis of single SNP effect on "ferritin(male)"-to-"appendicular lean mass(male)" UVMR results.

Leave-one-out plot using IVW method by sequentially re-evaluating the causal estimate after discarding one SNP at a time, which helps determine whether the overall effect is driven by the specific genetic variant. The black point denotes the causal effect estimate of ferritin on appendicular lean mass after discarding a certain SNP, and the black line signifies the 95% CI of estimate. The red point symbolizes the causal effect estimate of ferritin on appendicular lean mass, and the red line indicates the 95% CI of the estimate. **Abbreviations:** SNP = number of single-nucleotide polymorphism; UVMR = univariate Mendelian randomization; CI = confidence interval.

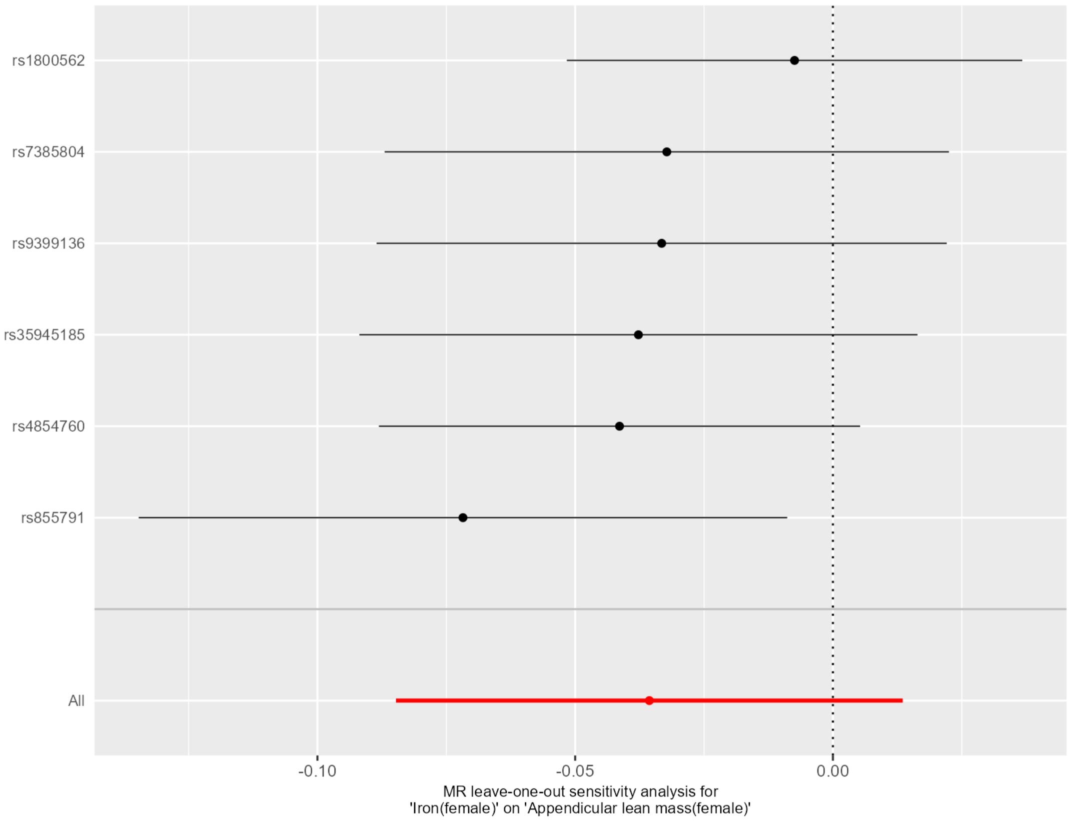

Supplementary Figure 43. Leave-one-out plot for sensitivity analysis of single SNP effect on “iron(female)”-to-“appendicular lean mass(female)” UVMR results.

Leave-one-out plot using IVW method by sequentially re-evaluating the causal estimate after discarding one SNP at a time, which helps determine whether the overall effect is driven by the specific genetic variant. The black point denotes the causal effect estimate of ferritin on appendicular lean mass after discarding a certain SNP, and the black line signifies the 95% CI of estimate. The red point symbolizes the causal effect estimate of ferritin on appendicular lean mass, and the red line indicates the 95% CI of the estimate. **Abbreviations:** SNP = number of single-nucleotide polymorphism; UVMR = univariate Mendelian randomization; CI = confidence interval.

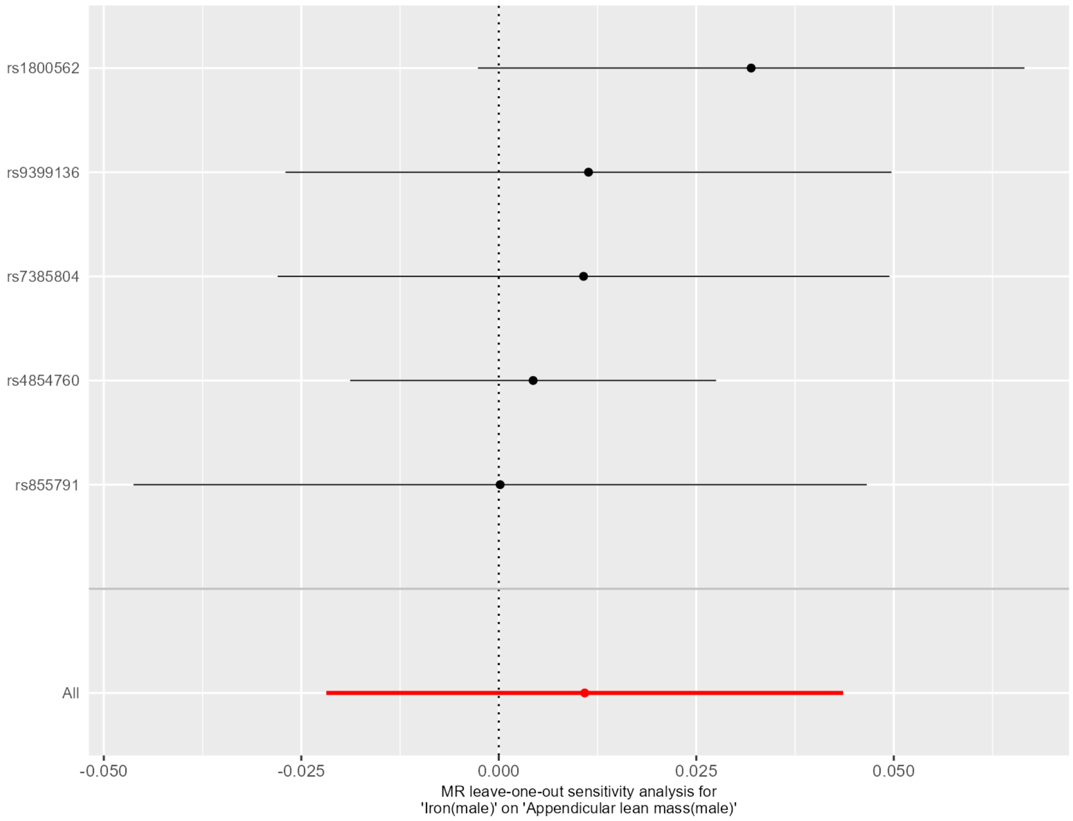

Supplementary Figure 44. Leave-one-out plot for sensitivity analysis of single SNP effect on "iron(male)"-to-"appendicular lean mass(male)" UVMR results.

Leave-one-out plot using IVW method by sequentially re-evaluating the causal estimate after discarding one SNP at a time, which helps determine whether the overall effect is driven by the specific genetic variant. The black point denotes the causal effect estimate of ferritin on appendicular lean mass after discarding a certain SNP, and the black line signifies the 95% CI of estimate. The red point symbolizes the causal effect estimate of ferritin on appendicular lean mass, and the red line indicates the 95% CI of the estimate. **Abbreviations:** SNP = number of single-nucleotide polymorphism; UVMR = univariate Mendelian randomization; CI = confidence interval.

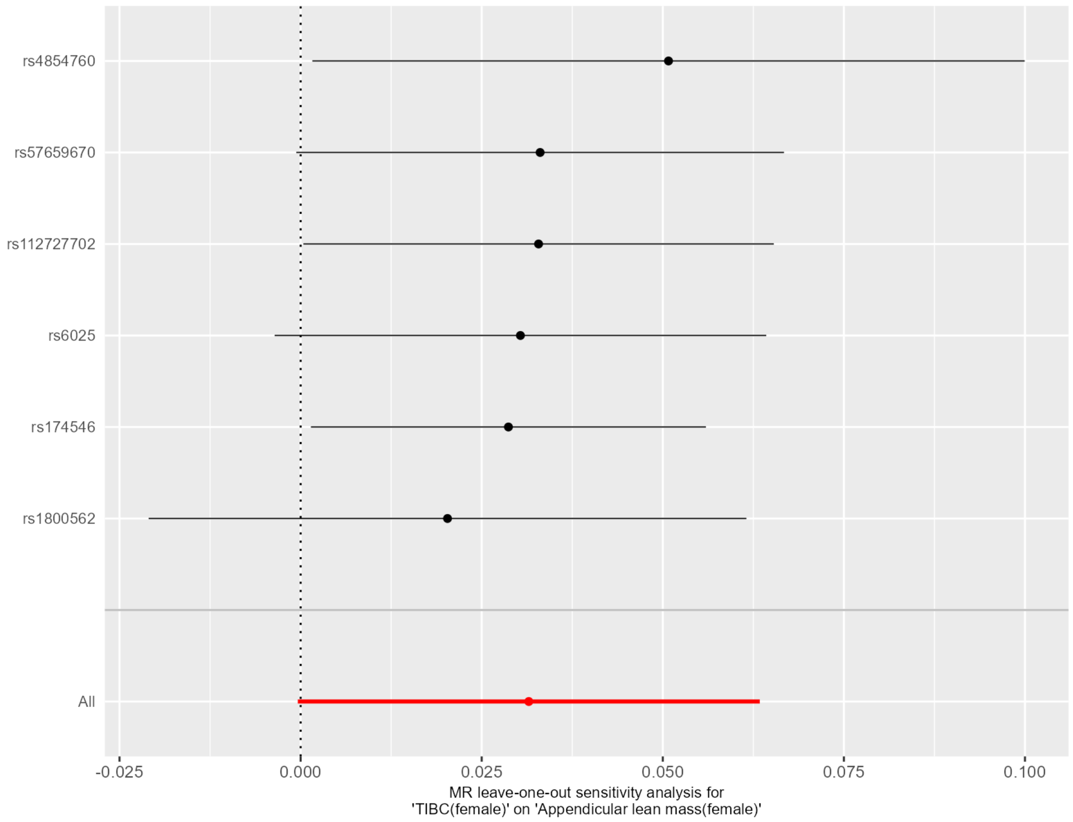

Supplementary Figure 45. Leave-one-out plot for sensitivity analysis of single SNP effect on "total iron binding capacity(female)"-to-"appendicular lean mass(female)" UVMR results.

Leave-one-out plot using IVW method by sequentially re-evaluating the causal estimate after discarding one SNP at a time, which helps determine whether the overall effect is driven by the specific genetic variant. The black point denotes the causal effect estimate of ferritin on appendicular lean mass after discarding a certain SNP, and the black line signifies the 95% CI of estimate. The red point symbolizes the causal effect estimate of ferritin on appendicular lean mass, and the red line indicates the 95% CI of the estimate. **Abbreviations:** SNP = number of single-nucleotide polymorphism; UVMR = univariate Mendelian randomization; CI = confidence interval.

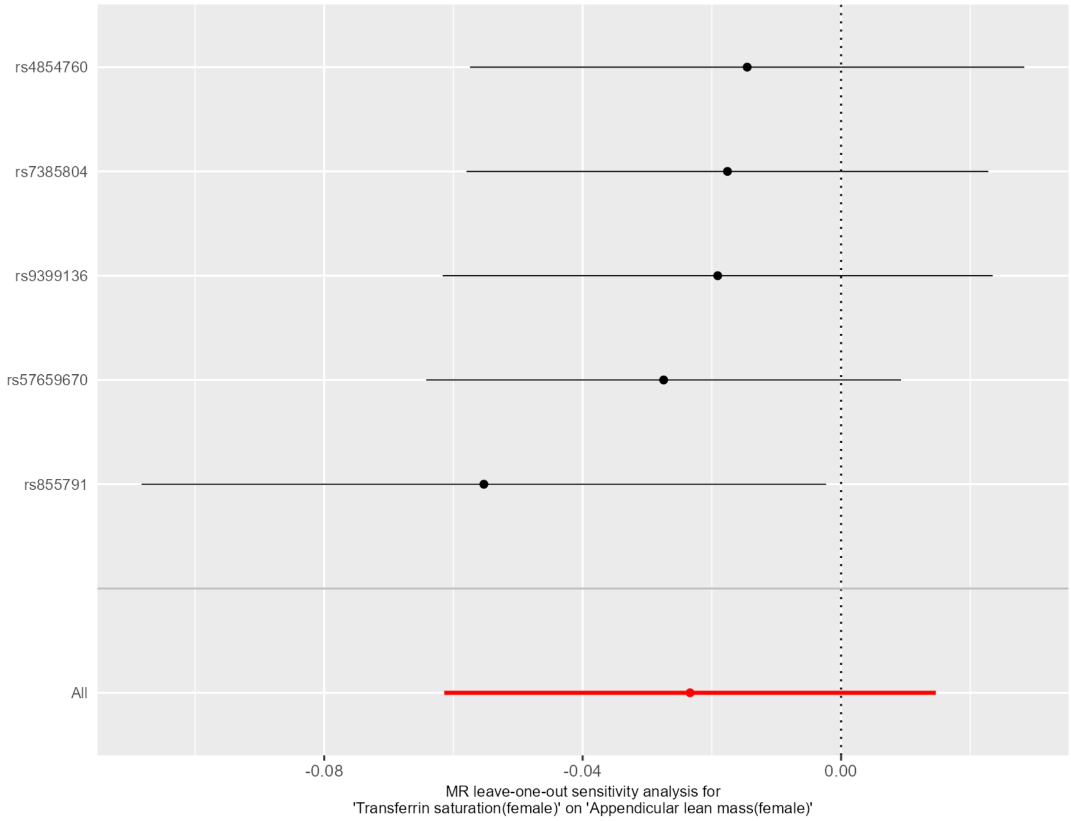

Supplementary Figure 46. Leave-one-out plot for sensitivity analysis of single SNP effect on "transferrin saturation(female)"-to-"appendicular lean mass(female)" UVMR results.

Leave-one-out plot using IVW method by sequentially re-evaluating the causal estimate after discarding one SNP at a time, which helps determine whether the overall effect is driven by the specific genetic variant. The black point denotes the causal effect estimate of ferritin on appendicular lean mass after discarding a certain SNP, and the black line signifies the 95% CI of estimate. The red point symbolizes the causal effect estimate of ferritin on appendicular lean mass, and the red line indicates the 95% CI of the estimate. **Abbreviations:** SNP = number of single-nucleotide polymorphism; UVMR = univariate Mendelian randomization; CI = confidence interval.

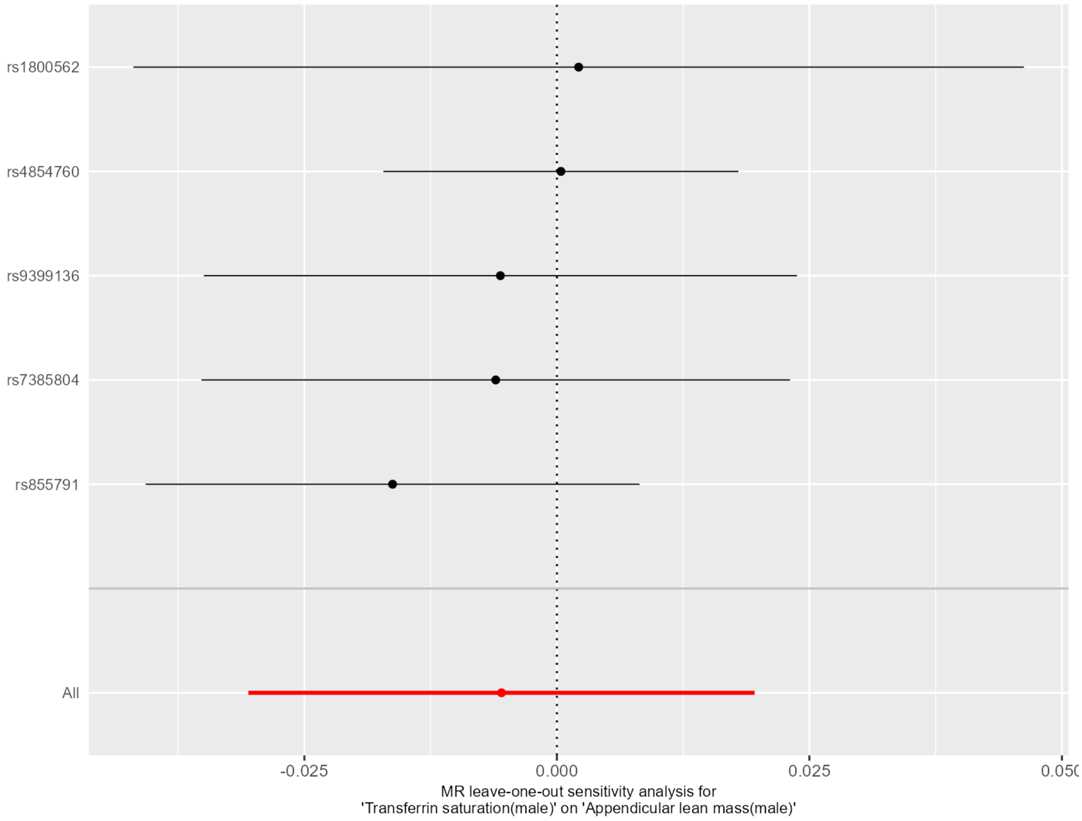

Supplementary Figure 47. Leave-one-out plot for sensitivity analysis of single SNP effect on "transferrin saturation(male)"-to-"appendicular lean mass(male)" UVMR results.

Leave-one-out plot using IVW method by sequentially re-evaluating the causal estimate after discarding one SNP at a time, which helps determine whether the overall effect is driven by the specific genetic variant. The black point denotes the causal effect estimate of ferritin on appendicular lean mass after discarding a certain SNP, and the black line signifies the 95% CI of estimate. The red point symbolizes the causal effect estimate of ferritin on appendicular lean mass, and the red line indicates the 95% CI of the estimate. **Abbreviations:** SNP = number of single-nucleotide polymorphism; UVMR = univariate Mendelian randomization; CI = confidence interval.
